# Identification of genetic markers for cortical areas using a Random Forest classification routine and the Allen Mouse Brain Atlas

**DOI:** 10.1371/journal.pone.0212898

**Published:** 2019-09-04

**Authors:** Natalie Weed, Trygve Bakken, Nile Graddis, Nathan Gouwens, Daniel Millman, Michael Hawrylycz, Jack Waters

**Affiliations:** Allen Institute for Brain Science, Seattle, Washington, United States of America; University of Pécs Medical School, HUNGARY

## Abstract

The mammalian neocortex is subdivided into a series of cortical areas that are functionally and anatomically distinct and are often distinguished in brain sections using histochemical stains and other markers of protein expression. We searched the Allen Mouse Brain Atlas, a database of gene expression, for novel markers of cortical areas. To screen for genes that change expression at area borders, we employed a random forest algorithm and binary region classification. Novel genetic markers were identified for 19 of 39 areas and provide code that quickly and efficiently searches the Allen Mouse Brain Atlas. Our results demonstrate the utility of the random forest algorithm for cortical area classification and we provide code that may be used to facilitate the identification of genetic markers of cortical and subcortical structures and perhaps changes in gene expression in disease states.

## Introduction

The mammalian neocortex is classified into a series of anatomically and functionally distinct regions or cortical areas [1,2]. Areas are often identified using histochemical stains and antibodies to visualize differences in protein expression across cortex. Examples include cytochrome oxidase histochemistry and antibodies against m2 muscarinic receptors [3]. Numerous differences in expression across cortical areas have been observed, including abrupt changes in expression at area borders, more graded changes between areas, gradients in expression across an area, and changes in cell-specific expression [4–11].

We reasoned that there may be genetic markers of cortical areas that have not been identified and that we might identify additional markers by screening the Allen Mouse Brain Atlas, a database containing in situ hybridization information for thousands of genes [12]. We developed numerical tools to screen the many thousands of images in the database, using a random forest algorithm to identify changes in gene expression at the boundaries of cortical areas defined in the Allen Mouse Brain Reference Atlas [13]. We searched for genes that exhibited an abrupt change in expression at an area border, rather than a difference in expression between two cortical areas. We found novel genetic markers for several areas. In addition, we provide code that searches the Allen Mouse Brain Atlas quickly and efficiently for differences in gene expression between cortical areas. With only minor modification, our code could be adapted to search for genes that mark other brain regions, including subcortical nuclei.

## Methods and results

Our aim was to identify genes with changes in expression at the borders of cortical areas in the mouse. From the Allen Mouse Brain Atlas, we took coronal in situ hybridization (ISH) data resampled to a canonical 3D reference space and overlaid the borders of cortical regions from the Allen Mouse Brain Reference Atlas, version 3. To identify genes with differential expression along these boundaries, we used a Random Forest algorithm, implemented in Python using the scikit-learn package.

### Top and flat-map projections

**Fig 1:**
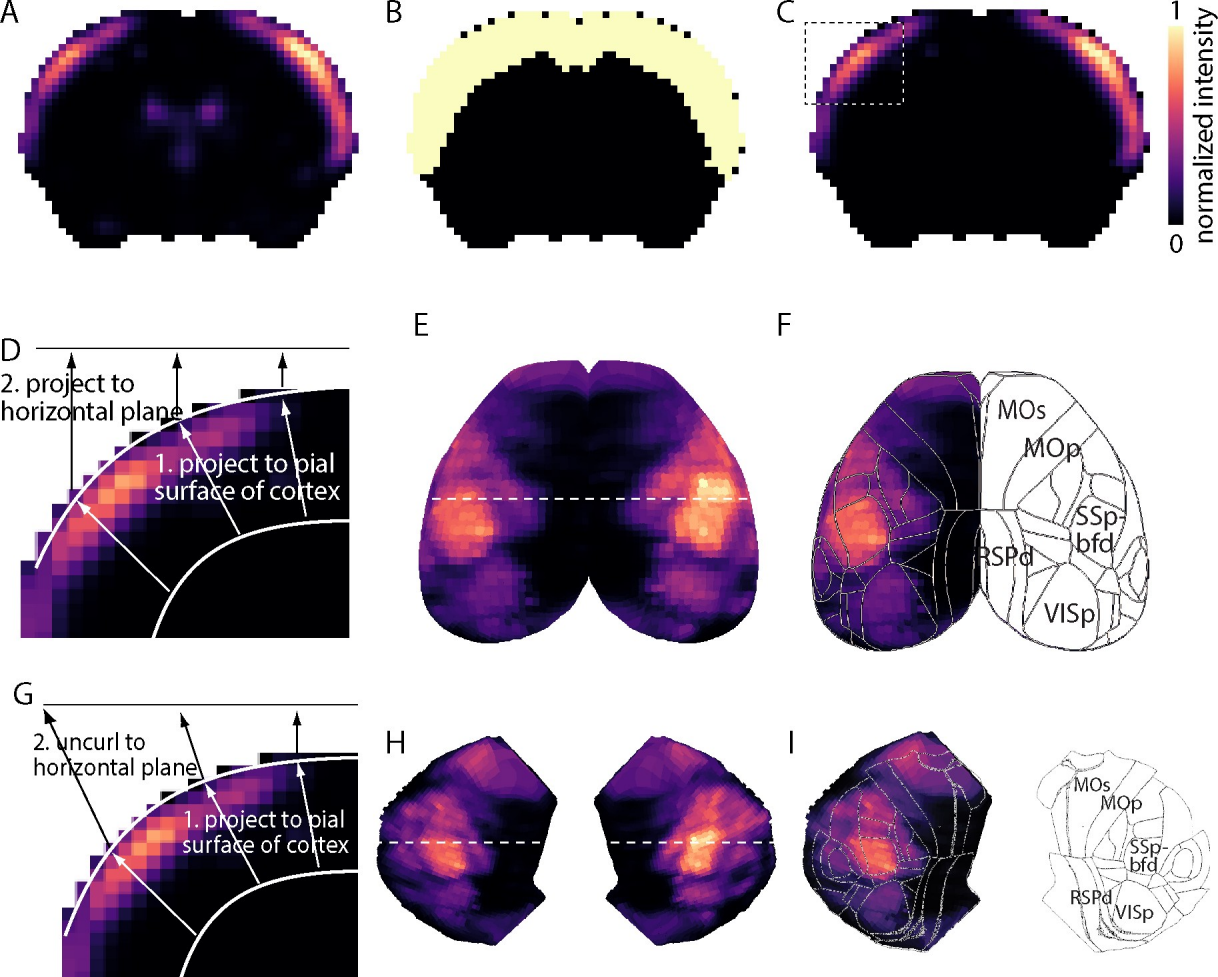
Creation of top projections from coronal images of gene expression. (A) Image of a coronal section from the Allen Mouse Brain Atlas. Gene: *Rorb*. ISH intensity is normalized to a range of 0 to 1. Color scale shown in panel C. (B) Binary cortex mask with value = 1 for cortical pixels and value = 0 for subcortical pixels. (C) Product of the images in A and B, resulting in ISH intensity values in cortical pixels and zero’s in subcortical locations. (D) Schematic illustration of the projection process used to generate top projections. (E) Top projection for *Rorb*. Dashed line indicates location of section in panel A. (F) Cortical boundaries from the Allen Mouse Brain Reference Atlas, overlaid onto the gene expression top view of panel E. (G) Schematic illustration of the projection process used to generate flat map top projections. (H) Flat map projection for *Rorb*. Dashed line indicates location of section in panel A. (I) Cortical boundaries from the Allen Mouse Brain Reference Atlas, overlaid onto the gene expression top view of panel H.

We obtained coronal ISH data for 4345 genes from the Allen Mouse Brain Atlas (http://mouse.brain-map.org/) at 200 μm resolution in male, 56-day-old C57BL/6J mice at 25-μm sections [12]. Code to download and analyze data sets is supplied as Supporting Information. Sagittal images were not explored in our study because many included only medial regions of the brain. The perspective that best captures many borders delineating cortical areas is the horizontal or ‘top’ projection. However, lateral cortical regions are severely underrepresented in top projections and we therefore generated a flat-map projection for each gene. Each projection was created in three steps, with the first two steps being common to both projections. Firstly, we isolated cortical fluorescence and eliminated fluorescence from subcortical structures by applying a mask derived from the Allen SDK (2015) structure_tree class (Fig 1B and 1C). Secondly, we created a maximum intensity surface projection: for each pixel on the cortical surface, we projected the fluorescence in the underlying tissue along a line perpendicular to the pial surface of cortex. One might think of this second step as creating a curved sheet of fluorescence intensity values at the surface of cortex. Finally, we projected these surface values to the horizontal plane, creating a top projection (Fig 1D) or we ‘unfurled’ the curved cortical sheet to create a flat map (Fig 1G).

All ISH data in the Allen Mouse Brain Atlas are spatially registered to the Allen Mouse Brain Reference Atlas (http://help.brain-map.org/display/mousebrain/Documentation), generated using 2D Nissl staining protocol, creating 132 coronal section references [12]. Hence all expression data utilized are inherently co-aligned with the Allen Mouse Brain Reference Atlas and the locations of brain areas can be readily superimposed on the ISH results. To locate cortical regions in the top projection and create a cortical area map, we extracted the corresponding cortical area masks using the structure_tree class and projected these masks to the horizontal plane, as described for ISH projections. Simplification of three-dimensional data into two dimensions allowed for fast quantitative analysis as well as easy visualization of expression patterns.

### Random forest algorithm

**Fig 2:**
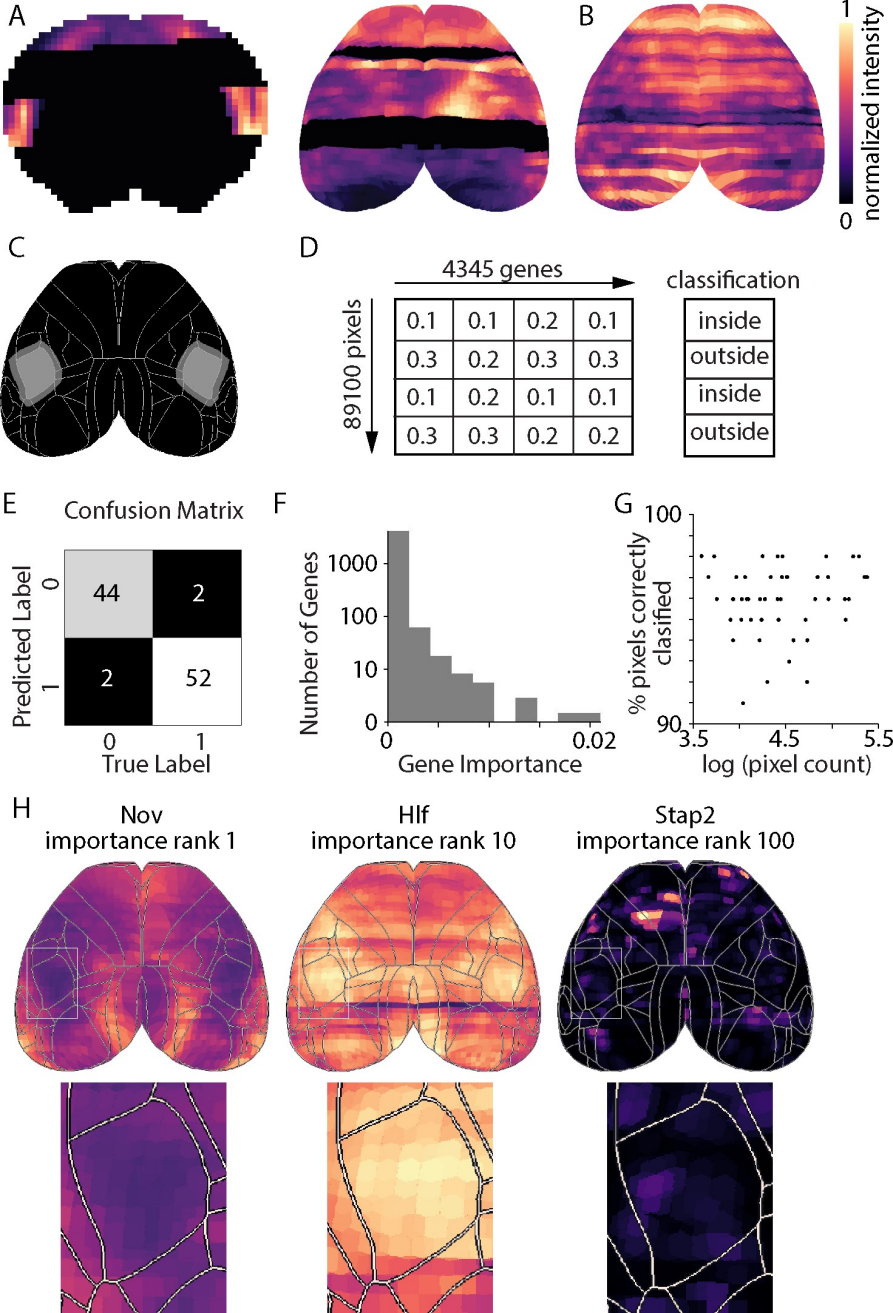
Random forest algorithm: Method and example results. (**A**) A coronal image (left) and the top projection (right) for gene *Nvl*. Note the missing data (black pixels). (**B**) Top view for gene *Adra1d*. Note the pronounced variation in density along the a-p axis. (**C**) Binary mask for primary somatosensory cortex barrel field (SSp-bfd). Light gray inside SSp-bfd; darker grey marks pixels in surrounding region. White lines: boundaries of cortical areas in Allen Mouse Brain Reference Atlas. (**D**) Schematic illustration of arrays input into Random Forest algorithm. Columns correspond to gene, rows to pixels in the top projection data set. Each value is an ISH luminance value. Classification of pixel is taken from the reference mask (panel C). (**E**) Confusion matrix output from Random Forest algorithm for SSp-bfd. 0 indicates point outside SSp-bfd, 1 indicates point inside SSp-bfd. (**F**) Gene importance histogram. Importance values approximate a logarithmic distribution. (**G**) Classification accuracy for all cortical areas (top and flat-map projections) shows no increase in accuracy with increasing area size. (**H**) Examples of genes that mark SSp-bfd, with overlaid Allen Mouse Brain Reference Atlas borders. *Nov* rank 1, importance 0.022. *Hlf* rank 10, importance value 0.0081. *Stap2* rank 100, importance 0.0018.

When examining the ISH results, two limitations became apparent. Firstly, there are gaps in some data sets, with missing data manifest as dark pixels in coronal images or dark medial-lateral bands in the top projections (Fig 2A). Secondly, there is pronounced section-to-section variability in mean fluorescence that appears as coronal banding or ‘stripes’ in top projections (Fig 2B). Coronal banding or top projection pixel z-score values did not follow patterns based on spatial organization. Together these two effects often result in variation in pixel values, independent of variation due to differential gene expression. These data properties complicate the comparison of fluorescence along the anterior-posterior axis and, thereby, the comparison of expression between cortical regions. Rather than attempt to mitigate these issues directly, we trained a Random Forest algorithm to classify pixels as either inside or outside each cortical region, essentially learning the variance in the data.

We examined 39 cortical regions from the Allen Mouse Brain Reference Atlas for potential gene markers. 45 cortical areas are described in the Reference Atlas. 6 cortical areas occupied <1000 pixels in projections and were deemed too small for our purposes, resulting in 39 regions that we analyzed. Areas explored are included in Table 1. Each search involved comparison of one cortical region to expression patterns of all genes, imputed as independent variables to the Random Forest algorithm. Random forest was implemented in Python using the scikit-learn package [14]. Nodes were determined by Gini Index criteria $\sum_{k=1}^{K}(p_{mk}(1‐p_{mk}))$. Each random forest consisted of 100 decision trees. Random state was initialized at 0. The importance of each variable was also determined by Gini Index criteria–reduction in Gini Index each time a split occurred was attributed to the variable, and that variable-associated reduction was divided by total reduction in Gini Index across the entire random forest to return the variable importance value. Total variable importance across all genes summed to one.

**Table 1:** List of potential genetic markers. Potential cortical boundary genetic markers, listed by cortical region, as identified by Random Forest variable importance classifier. All explored regions listed. Regions with no listed genes displayed no clear potential genetic marker. Each gene was identified independently.

Cortical Region	Potential Genetic Markers
Somatomotor	*Ube4b*, *Lin7a*, *Man1a*, *Wnt7b*
- Primary	
- Secondary	
Somatosensory	*Adam33*, *Vgf*, *Kcnip3*, *Rorb*, *Pcdh20*
- Primary	*Pvalb*
-- Primary, barrel	*Rspo1*
-- Primary, nose	
-- Primary, l. limb	
-- Primary, mouth	
-- Primary, u. limb	
-- Primary, trunk	*Trpc4*
-- Primary, unassigned	
Auditory*	*Tspan33*, *Coch
- Primary*	*Unc5d*, *Zmat4*, *Rspo1*, *Cdh24*, *Ptn, *Chn2
- Dorsal*	*Dgkb*
- Posterior*	
- Ventral*	
Visual	*Ptgs2*
- Primary	*Slc24a3*, *Thbs2*
- Lateral	
- Anterolateral	
- Anteromedial	*Stard7*
- Posterolateral	
- Posteromedial	
- Postrhinal	*Bmp3*, *Plxnd1*, *Spint2*
- Laterointermediate	
- Rostrolateral	
Anterior Cingulate	
- Dorsal	
Retrosplenal	*Cacnb3*, *Npsr1*, *Mas1*, *C1ql2*
- Lateral agranular	
- Dorsal*	*Tmem215*, *Npnt*
- Ventral*	*Adcy8*, *Scnn1a*, *Sm1399*, *Necab2*, *Thsd7a*, *Dpysl5
Temporal Association*	*Lifr
Ectorhinal*	*Kctd4
Medial Orbital*	*Dtx1
Visceral*	
Prelimbic*	
Frontal Pole*	*Serpinf1

Asterisks indicate regions explored and genes identified with flat map projections.

The Random Forest was trained to distinguish pixels within a cortical region from pixels in the surrounding area outside of the cortical region. The inputs to the Random Forest algorithm were the gene expression fluorescence intensity values of all 4345 genes for each pixel and the corresponding labels for each pixel as cortical region or surrounding region. Surrounding pixels were identified by dilating the region mask by 30 iterations using SciPy ndimage package in Python, translating to roughly 30 pixels in distance in each direction. Pixels were split into training and test sets, with 100 randomly selected pixels held out as a test set and the remaining pixels forming the training set. The test set was less than 1% of total pixels classified for each cortical area with even small regions such as frontal pole, consisting of 76,852 pixels in the flat-map projection. Data was divided using the scikit-learn model_selection package in Python. Hence the training array input into the Random Forest algorithm consisted of a 2D array of dimensions *N*−100 by 4345, where *N* is the number of pixels within the dilated mask and 4345 is the number of genes, and each cell in the array corresponding to a luminance value of the pixel. A second array of dimensions *N*−100 by 1 indicated the binary labels, inside or outside the cortical region (Fig 2D). After training, performance of the algorithm was tested on the held-out pixels (array dimensions 100 by 4345) for which the binary classification training was withheld. Withheld pixels were randomly selected, creating a test set that was representative of the cortical area: proportional inside and outside the cortical region and of random distance from the cortical area border. Hence the algorithm returned the cross-validated binary classification for 100 withheld pixels, which was compared to known classification and used to plot a confusion matrix (Fig 2E), summarizing performance of the Random Forest. For each cortical region, two outputs from the random forest were analyzed: a confusion matrix, indicating the success rate of the classification algorithm; and the list of all 4345 genes, ranked in decreasing order of variable importance, where importance is a pseudo-measure of the expression predictive power across the cortical border.

Results for primary somatosensory barrel field are illustrated in Fig 2E–2G. The model correctly classified 52 of 54 test pixels within the barrel field and 44 of 46 test pixels outside barrel field, resulting in a combined model accuracy of 96% (Fig 2E). Most genes exhibited low variable importance (Fig 2F). We ranked genes by their random forest variable importance values. The gene with rank 1 exhibited a distinct change along the border (Fig 2G). The gene with rank 10 exhibited a subtler change and the gene at rank 100 exhibited no obvious change along the border (Fig 2G). Hence the Random Forest algorithm accurately classified most pixels and, via a ranked list of genes, identified a short list of genes that might act as putative genetic markers of the cortical region.

### Genetic markers of cortical areas

For each cortical area, we manually inspected the projections for the 10 genes with the highest ranks. Occasionally, a potential marker gene differed in expression across a border in only one hemisphere or exhibited a gradient of expression across a cortical area, not just at the area border. Such genes were identified manually and excluded. We identified potential genetic markers for 19 cortical areas. 0–6 marked genes were identified per area, with only *Rspo1* identified as a marker for more than one region (barrel cortex and primary auditory cortex, Table 1).

Of the six markers identified by Hawrylycz *et al*. [5], three were identified in our results (*Man1a*, somatomotor; *Rorb*, somatosensory; *Scnn1a*, ventral retrosplenial). One gene was expressed in an area that was excluded from our analysis (*Smoc1*, gustatory cortex). Two genes were in areas that were marked by many other genes, which is perhaps why they failed to appear in the top 10 genes for these areas (*Hap1*, ectorhinal; *Rreb1*, retrosplenial).

**Fig 3:**
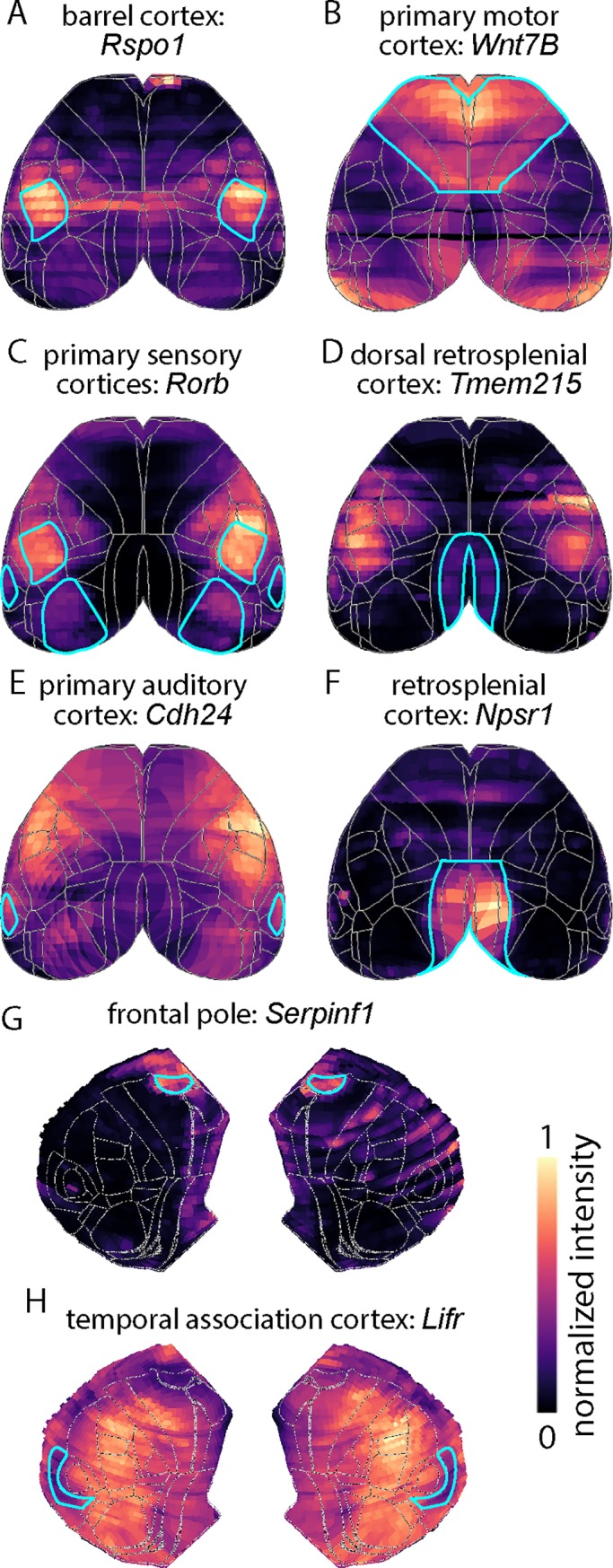
Examples of markers for cortical areas. (A) R-Spondin 1 labels primary somatosensory cortex barrel field. (B) Wnt Family Member 7B labels primary motor cortex. (C) Retinoid-Related Orphan Receptor, Beta labels primary sensory areas. (D) Transmembrane Protein 215 labels dorsal retrosplenial cortex. (E) Cadherin 24 labels primary auditory cortex. (F) Neuropeptide S Receptor 1 labels retrosplenial cortex. (G) Serpin Family F Member 1 labels frontal pole. (H) Leukemia Inhibitory Factor Receptor labels temporal association cortex. Panels A-F provide examples of genes identified in top projections, panels G and H of genes identified in flat maps. Cortical area of interest is marked with cyan border.

Examples of expression patterns are provided in Fig 3. For primary somatosensory cortex barrel field, we identified *Rspo1* as a strong candidate gene (Fig 3A). Expression of *Rspo1* is relatively high in the barrel field, moderate through somatosensory areas, and low in motor cortex. We identified four potential markers for somatomotor cortex, including *Wnt7b* (Fig 3B), but we found no markers for primary or secondary motor cortex. *Rorb* was also identified as a potential marker, specifically for primary sensory cortices (Fig 3C). This provided an additional positive control that our method was robust and effective, as *Rorb* is an established marker for primary sensory areas [5, 15]. *Cdh24* marked primary auditory cortex (Fig 3E). We found multiple genes that labeled all or subregions of retrosplenial cortex. For example, *Tmem215* marked dorsal retrosplenial cortex (and primary somatosensory cortex) and *Npsr1* marked all of retrosplenial cortex (Fig 3D and 3F). In flat maps, *Serpinf1* was identified as a marker of the frontal pole (Fig 3G) and temporal association cortex was marked by *Lifr* (Fig 3H). For cortical areas present in top and flat-map projections, importance rankings were generally similar.

Some genes exhibited mediolateral stripes in the top projection (e.g. *Rspo1*, *Wnt7B*, *Npsr1*, *Lifr)*, indicating that our method is tolerant to the missing data and expression-independent variability in signal and we conclude that genes with expression-independent variability were not excluded by our analysis.

### Cellular basis of potential genetic markers

**Fig 4:**
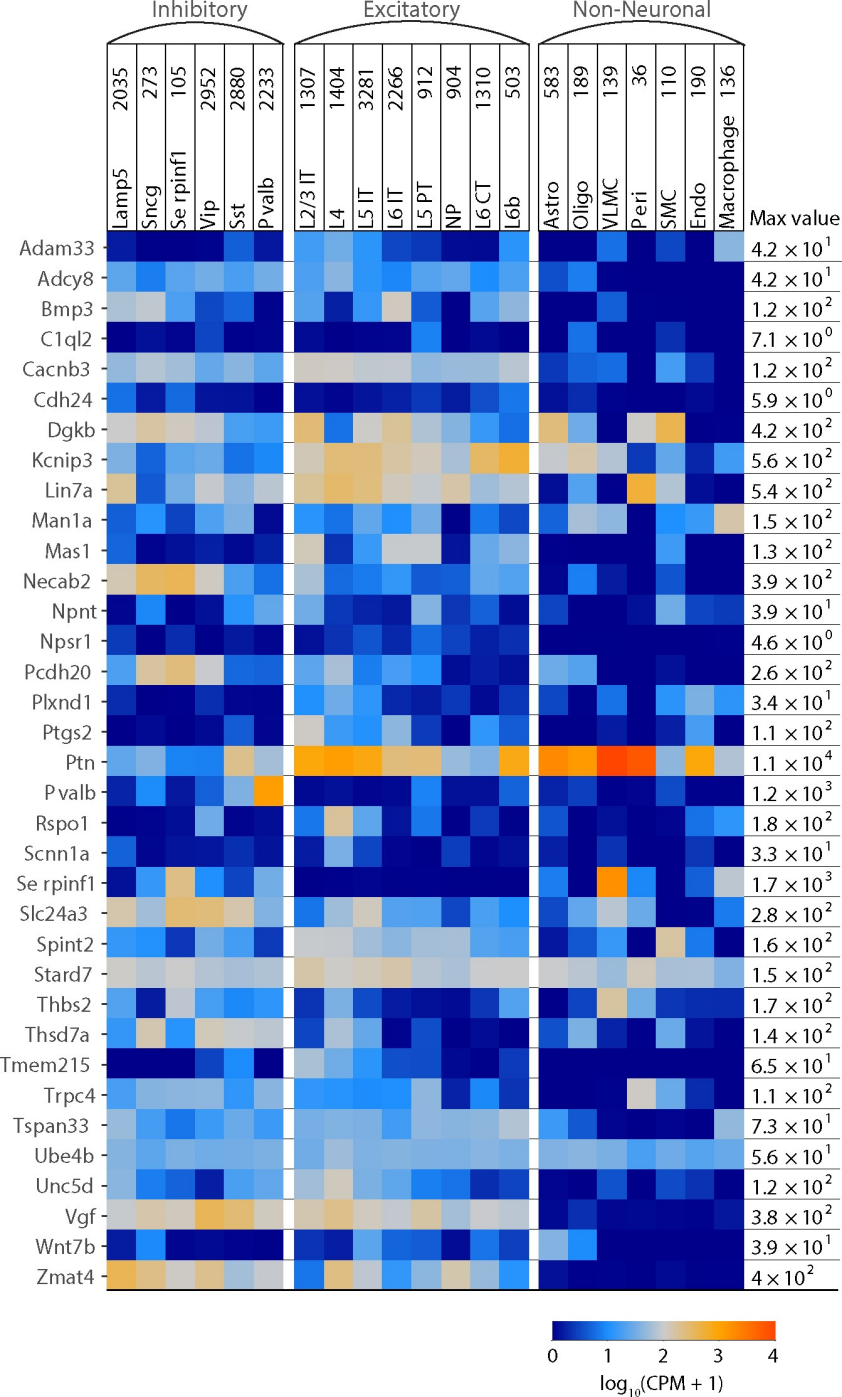
Single cell RNA-sequencing expression plot. Expression of top-projection identified potential areal marker genes in mouse cortical cells grouped into subtypes of three major cell classes: inhibitory neurons, excitatory neurons, and non-neuronal cells. Expression data was measured by RNA-sequencing of single cells isolated from Primary Visual Cortex (VISp) and Anterior Lateral Motor Cortex (ALM). Expression is taken from Smart-seq version 4, as log-transformed average counts per million (CPM) of intronic and exonic reads. Color corresponds to expression value, with warmer colors indicating high expression and cooler colors indicating low expression. Max value indicates the maximum expression per gene, measured in average CPM per cluster. CPM = counts per million reads.

Our Random Forest searches, applied to ISH data, identified genes that marked cortical area borders, but provided no insight into the cellular basis of the genetic markers. Does, for example, a change in gene expression result from an abrupt change in the density of a cell type with unique gene expression; or might the border result from a change in gene expression by a cell type that straddles the border? Gene expression in primary visual cortex and anterior lateral motor cortex has been studied using single-cell RNA sequencing [16]. From this transcriptomic data set and associated analysis tools (https://github.com/AllenInstitute/scrattch.vis), we examined cell types that express the marker genes identified in this study by top view projections (Fig 4). Markers were expressed in many different cell types. Several genes (*Adcy8*, *Bmp3*, *Cacnb3*, *Npsr1*, *Vgf*, *Zmat4*) were expressed mostly in neurons and not non-neuronal cells, suggesting that the border-related change in expression of these genes was neuronal. Some genes were expressed mostly in a cell sub-population, suggesting that there is likely a border-related change in the density of these cells or of their expression of one gene. For example, *Rspo1*, *Serpinf1* and *Man1a* are expressed in layer 4 excitatory neurons, vascular and leptomeningeal cells (VLMC) and macrophages, respectively. Our results are consistent with changes in gene expression marking cortical areas arising from changes in cell density in some instances (e.g. *Necab2* and *Rspo1* display preferential expression in specific cell types) and from changes in gene expression within a cell population in other instances (e.g. *Stard7 and Ube4b* display uniform expression across cell types). Importantly, both patterns of genetic expression were detected by our Random Forest analysis of ISH data.

## Discussion

We used a Random Forest algorithm to identify a short list of potential gene markers from thousands of candidate genes, applying this approach to 39 cortical regions in the mouse. Our results identified 44 putative markers, marking 19 of the explored regions.

The spatial resolution and number of genes in the database places limits on the conclusions we can draw. Firstly, the voxel size of the ISH quantification in the database is 200 μm. Once missing and variable data is considered, the maximum accuracy we can hope to achieve is on the order of hundreds of micrometers, resulting in an imperfect match between area borders and gene expression. Subsequent experiments such as immunostaining for the genes we have identified would be necessary to confirm our results and to assess the accuracy with which each gene marks borders. Furthermore, the database includes coronal ISH images for 4345 genes. It may be that genes not sampled here mark some of the 20 cortical regions for which we were unable to identify markers. Repeating our analysis on a larger or higher-resolution data set, should one become available, might identify further markers.

We elected to look for borders in 2-dimensional projections, derived from ISH data sets in 3D. Two-dimensional analysis collapsed one dimension, vastly reducing the size of the data set and decreasing computation time. In 2D, each projection data set was generated in ~3 days and each random forest calculation ~2 minutes. Performing the calculations in 3D would have increased the computational load prohibitively. Having not compared 2D and 3D results for each gene, we cannot definitively determine whether 2D and 3D analysis produce equivalent results, but there is no reason to think that 3D analysis would be advantageous for most cortical areas. For most areas, columns (and therefore borders) descend into cortex perpendicular to the tangent plane at the cortical surface. Our top and flat-map projection code calculates the maximum ISH intensities along paths perpendicular to the surface tangent. Hence the 3D to 2D dimension reduction occurs parallel to cortical columns, not across them, maintaining integrity of borders during the 3D to 2D conversion. (One way to check on this preservation is to generate projections of neighboring cortical areas. Neighboring cortical areas, as annotated in the Common Coordinate Framework, display very little overlap in top projections and flat-map projections.) 2D projection of cortical maps is common [17, 18], largely because borders are well preserved. The exception to this generalization that borders are respected during the 3D to 2D conversion would be for areas with borders that do not run perpendicular to the surface tangent.

Alternatives to our Random Forest approach might include simpler statistical tests (e.g. t-tests) to compare gene expression across a border. The Random Forest approach has advantages over simpler tests, such as increased robustness to non-monotonic expression patterns. ISH images commonly displayed pronounced variability, including regions of missing data. We do not know whether such variability could be eliminated, permitting use of simpler tests, but found that Random forest classification identified changes in gene expression at borders without the need for extensive pre-processing of the underlying data sets.

Variable importance may be inaccurately skewed towards higher sampled variables or continuous data types, and thus unusable; however, because our predictor variables exhibit identical scale of measurement and data type, importance rank can be taken as unbiased [19]. Random forest uses a bootstrapped subset of variables at each splitting node when building decision trees. By accumulating many splits on previously subdivided pixels, genes are evaluated at subregions of the cortical area. Given this property, we find that occasionally genes with relatively high variable importance display marking of a single border rather than the entire cortical area. However, if a gene exhibits clear marking of all cortical borders, it is shown with higher variable importance than an alternate gene expression pattern marking only a single border. Random forest is an accurate, computationally efficient, and easily interpretable method of classification. This was important as many of our data sets, especially for larger cortical areas like somatosensory areas, reached sizes of almost 250,000 pixels, evaluated at 4345 genes. Each output predicted took less than a minute to compile the data set, run computations, and produce outputs on a desktop computer. By maximizing concurrent computation across all available cores, the time required to run is minimized while not sacrificing predictive power of our model, as exhibited by the high accuracy of the Random Forest.

By dilating the cortical area mask a small amount instead of comparing the area of interest to the entire cortex, we allowed for differential expression of the gene in more distant parts of cortex. This is by design, as expression far from the desired cortical region does not impact the ability of the gene to mark the border. However, potential uniquely expressed genes are still a subset of those that can be identified with our method, and our method could be readily modified to solely identify uniquely expressed genes. Similarly, the method could be easily extended to investigate laminar differences or expression patterns in subcortical structures as masks for cortical layers and for subcortical structures are included in the Allen Mouse Brain Reference Atlas.

Through our method and results, we have found novel genetic markers for cortical areas. Our method can be extended to additional cortical or to sub-cortical regions via the supplied code, and to other species where appropriate data sets are available. Should higher-resolution ISH data become available, our method will likely allow the exploration of gene expression differences between smaller brain regions and the patterns of expression within individual brain areas.

## Supporting information

S1 Fig Top_Projection. Jupyter notebook code for generation and analysis of top projections.
(IPYNB)

S2 Fig Top_Projection. Jupyter notebook code for generation and analysis of top projections, html version.
(HTML)

S3 Fig Flatmap_Projection. Jupyter notebook code for generation and analysis of horizontal projections.
(IPYNB)

S4 Fig Flatmap_Projection. Jupyter notebook code for generation and analysis of horizontal projections, html version.
(HTML)
