# Supplementary material for: Identification of genetic markers for cortical areas using a Random Forest classification routine and the Allen Mouse Brain Atlas
Source: PLoS One. 2019 Sep 4;14(9):e0212898. doi: 10.1371/journal.pone.0212898 (PMC6726226; doi:10.1371/journal.pone.0212898)
Supplement: S2 Fig — Jupyter notebook code for generation and analysis of top projections, html version. (HTML) [file pone.0212898.s002.html]

Top\_Projection


## *Only modify "system specific modifications" and "find genes to mark specific region" box*¶

- Download attached files and point via system specific modifications to: CCF\_borders\_10um.tif, top\_view\_paths\_10.h5, gridAnnotation.mhd via "system specific modifications" cell
- Preallocate a folder for storing gene expression top view images, designated as searchpath

  - MAKE FOLDER PRIOR TO RUNNING CELL

In [1]:

```
import os
import numpy as np
import nrrd
import scipy.ndimage
from scipy.stats import mannwhitneyu as mw
import matplotlib.pyplot as plt
%matplotlib inline
from glob import glob
from sklearn.ensemble import RandomForestClassifier
from sklearn.model_selection import train_test_split
import tifffile as tiff 
from glob import glob
from sklearn.metrics import confusion_matrix
import seaborn as sns; sns.set()
import pandas as pd
import h5py
import SimpleITK as sitk 
import urllib, urllib2
import json
from IPython.html.widgets import interact
from math import log10
import zipfile

from allensdk.core.mouse_connectivity_cache import MouseConnectivityCache
mcc = MouseConnectivityCache(manifest_file='connectivity/manifest.json', resolution=10)
from allensdk.api.queries.ontologies_api import OntologiesApi
from allensdk.core.structure_tree import StructureTree
from allensdk.core.reference_space import ReferenceSpace
from allensdk.api.queries.mouse_connectivity_api import MouseConnectivityApi
from allensdk.config.manifest import Manifest
```

```
/Users/natalieweed/anaconda2/lib/python2.7/site-packages/sklearn/ensemble/weight_boosting.py:29: DeprecationWarning: numpy.core.umath_tests is an internal NumPy module and should not be imported. It will be removed in a future NumPy release.
  from numpy.core.umath_tests import inner1d
/Users/natalieweed/anaconda2/lib/python2.7/site-packages/IPython/html.py:14: ShimWarning: The `IPython.html` package has been deprecated since IPython 4.0. You should import from `notebook` instead. `IPython.html.widgets` has moved to `ipywidgets`.
  "`IPython.html.widgets` has moved to `ipywidgets`.", ShimWarning)
```

#### System specific modifications¶

In [2]:

```
# File Locations
## CCF_borders_10um.tif LOCATION: 
cortextif_path = r"\\allen\programs\braintv\workgroups\nc-ophys\Jack\Natalie_Weed\Natalie\files\CCF_borders_10um.tif"

## top_view_paths_10.h5 LOCATION:
view_file = r"\\allen\programs\braintv\workgroups\nc-ophys\Jack\Natalie_Weed\Natalie\files\top_view_paths_10.h5"

## gridAnnotation.mhd LOCATION:
annot_200_img = sitk.ReadImage(r"\\allen\programs\braintv\workgroups\nc-ophys\Jack\Natalie_Weed\Natalie\files\gridAnnotation.mhd")

# Where images will be saved to
## Make a folder for gene maps to be directed to prior to running this cell
searchpath = r'E:/genemaps/coronal/'
```

# Code Explanation, part 1¶

Mapping of gene expression data from the mouse brain atlas coronal data to common coordinate frame using projections along paths to edge of cortex, and subsequent projection to a horizontal plane as viewed from the top of the brain. This code will pull data from the Allen server and create tif images of each experiment, storing the images in the path that the notebook is stored in. If images are already generated (with proper nomenclature), they will not be recreated. Images are named as "genename\_experimentnumber.tif". No external variables are inputted. No variables are explicitly returned. Images are saved to searchpath. Names of mice are printed. Dash is printed after name of mouse if top view image already exists in folder.

Once images are generated, the specific mice identification can also be printed out using the view\_mouse function.

In [3]:

```
def get_paths_lut(view_file):
    # open view index file
    vi = h5py.File(view_file, 'r')
    
    # initialize
    lut = vi['view lookup'][:]
    paths = vi['paths'][:]
    vi.close()
    return {'lut': lut, 'paths': paths}

def create_top_view_projection(image, top_view):
    # initialize
    output = np.zeros(top_view['lut'].shape + (2,), image.dtype)
    output[:,:,1] = 0
    dims_10 = (1320, 800, 1140)
    dims_200 = (67, 41, 58)

    def max_along_path(pid):
        path = top_view['paths'][pid,:]
        path_200 = np.array(np.unravel_index(path, dims_10))
        path_20 = np.ravel_multi_index((path_200 / 20).astype(int), dims_200)
        arr = image.flat[path_20]
        idmax = np.argmax(arr)
        vmax = arr[idmax]
        indmax = path[idmax]

        return vmax, indmax

    ind = np.where(top_view['lut'] > -1)
    output[ind] = map(max_along_path, top_view['lut'][ind])

    return output[:,:,0], output[:,:,1].astype(np.int32)

def get_section_image(section_id):
    grid_download_template = "http://api.brain-map.org/grid_data/download/{:s}?include=density"

    grid_download_url = grid_download_template.format(str(section_id))
    urllib.urlretrieve(grid_download_url, "griddata.zip")

    with zipfile.ZipFile("griddata.zip", "r") as zip_ref:
        zip_ref.extractall("griddata")

    img = sitk.ReadImage("griddata/density.mhd")
    arr = sitk.GetArrayFromImage(img)
    arr = np.swapaxes(arr, 0, 2)
    return arr

def apply_mask(griddata, mask):
    griddata[mask == 0] = -1
    return griddata

def create_map(section_id, gene_name, mask, top_view):
    filename = os.path.normpath(searchpath + gene_name + "_" + str(section_id) + ".tif")
    if os.path.isfile(filename):
        print "_"
    else:
        data = get_section_image(section_id)
        data = apply_mask(data, mask) # to apply isocortex mask
        output, depth = create_top_view_projection(data, top_view)
        output = output.astype(float)
        tiff.imsave(filename, output)
        
def images_main():
    
    rma_url = ("http://api.brain-map.org/api/v2/data/query.json?criteria=" +
               "model::SectionDataSet,rma::criteria,[failed$eqFalse]," +
               "plane_of_section[name$eqcoronal],products[name$eq'Mouse%20Brain']," +
               "treatments[name$eqISH],rma::include,genes," +
               "rma::options[only$eq'genes.acronym,id'][num_rows$eqall]")
    
    response = urllib2.urlopen(rma_url).read()
    data = json.loads(response)
    sections = {v["id"]: v["genes"][0]["acronym"] for v in data["msg"]}

    for section_id in sections:
        print sections[section_id].replace('*', '_')
        create_map(section_id, sections[section_id].replace('*', '_'), isocortex_200_mask, top_view)
        
def create_top_view_projection_masks(image, top_view):
    # initialize
    output = np.zeros(top_view['lut'].shape + (2,), image.dtype)
    output[:,:,1] = 0
    dims_10 = (1320, 800, 1140)

    def max_along_path(pid):
        path = top_view['paths'][pid,:]
        path_200 = np.array(np.unravel_index(path, dims_10))
        path_20 = np.ravel_multi_index((path_200 ).astype(int), dims_10)
        arr = image.flat[path_20]
        idmax = np.argmax(arr)
        vmax = arr[idmax]
        indmax = path[idmax]

        return vmax, indmax

    ind = np.where(top_view['lut'] > -1)
    output[ind] = map(max_along_path, top_view['lut'][ind])

    return output[:,:,0], output[:,:,1].astype(np.int32)

def create_map_single(data, top_view, name):
    output, depth = create_top_view_projection_masks(data, top_view)
    output = output.astype(float)
    tiff.imsave(name,output)
        
def image_main_single(index,name):
    filename = os.path.normpath( name) 
    if os.path.isfile(filename):
        print "reference already created"
    else:
        data = rsp.make_structure_mask([index])
        create_map_single(data, top_view, name)
    
def view_mouse():
    
    rma_url = ("http://api.brain-map.org/api/v2/data/query.json?criteria=" +
               "model::SectionDataSet,rma::criteria,[failed$eqFalse]," +
               "plane_of_section[name$eqcoronal],products[name$eq'Mouse%20Brain']," +
               "treatments[name$eqISH],rma::include,genes," +
               "rma::options[only$eq'genes.acronym,id'][num_rows$eqall]")
    response = urllib2.urlopen(rma_url).read()
    data = json.loads(response)
    sections = {v["id"]: v["genes"][0]["acronym"] for v in data["msg"]}
    for section_id in sections:
        print section_id, sections[section_id]
```

##### Making the reference space, as taken from AllenSDK¶

In [4]:

```
# the annotation download writes a file, so we will need somwhere to put it
annotation_dir = 'annotation'
Manifest.safe_mkdir(annotation_dir)

annotation_path = os.path.join(annotation_dir, 'annotation.nrrd')

# this is a string which contains the name of the latest ccf version
annotation_version = MouseConnectivityApi.CCF_VERSION_DEFAULT

mcapi = MouseConnectivityApi()
mcapi.download_annotation_volume(annotation_version, 10, annotation_path)

annotation, meta = nrrd.read(annotation_path)

oapi = OntologiesApi()
structure_graph = oapi.get_structures_with_sets([1])  # 1 is the id of the adult mouse structure graph

# This removes some unused fields returned by the query
structure_graph = StructureTree.clean_structures(structure_graph)  

tree = StructureTree(structure_graph)

# build a reference space from a StructureTree and annotation volume, the third argument is 
# the resolution of the space in microns
rsp = ReferenceSpace(tree, annotation, [10, 10, 10])
```

```
2018-10-09 16:30:07,601 allensdk.api.api.retrieve_file_over_http INFO     Downloading URL: http://download.alleninstitute.org/informatics-archive/current-release/mouse_ccf/annotation/ccf_2017/annotation_10.nrrd
```

## Isocortex mask¶

Make Isocortex mask in 2 dims and 3 dims

In [8]:

```
# Global files
top_view = get_paths_lut(view_file)
cortextif = tiff.imread(cortextif_path)
```

In [5]:

```
cortex = tree.descendant_ids([315])[0]
annot_200 = sitk.GetArrayFromImage(annot_200_img)
annot_200 = np.swapaxes(annot_200, 0, 2)

isocortex_200_mask = np.zeros_like(annot_200, dtype=np.uint8)
for sid in cortex:
    isocortex_200_mask[annot_200 == sid] = 1
```

In [6]:

```
name = 'isocortexmask.tif'
image_main_single(315,name)
isocortexmask_2d = tiff.imread(name)
```

```
reference already created
```

## Generate Gene Expression Images (Horizontal view, top projections)¶

In [9]:

```
images_main()
```

```
Inpp5a
_
Snap47
_
Nvl
_
Gtf3c2
_
Acadl
_
Baiap3
_
S100a6
_
Clock
_
Mageh1
_
Sncg
_
Hnrnph2
_
Nnat
_
Cdca7
_
Prkag2
_
Uggt1
_
Dopey2
_
Anln
_
Hepacam
_
Setd5
_
Cnih3
_
Thap1
_
TC1541413
_
Htr1a
_
9130024F11Rik
_
Mansc1
_
Pmch
_
Prune2
_
Il1rap
_
Acta1
_
Pcdh9
_
Actc1
_
Actr10
_
Ctla4
_
Rell1
_
Actr2
_
Fut8
_
Il34
_
9130024F11Rik
_
Adcy2
_
Scarb2
_
Adcy3
_
Adcy4
_
Adcy5
_
Adcy6
_
Adcy9
_
Rps19
_
Agpat3
_
Agt
_
Mllt11
_
Akap8
_
Anp32a
_
Mt3
_
Capsl
_
Etnppl
_
Gata2
_
Cdc42ep1
_
Rab6a
_
App
_
Ifi205
_
Spag5
_
Runx2
_
Arf3
_
Arhgap5
_
Hgf
_
Arx
_
Recql5
_
Thbd
_
Cit
_
Tmeff2
_
Atp5j
_
Htr4
_
Atp6v0a1
_
Atp6v1b2
_
Nes
_
Tmem50b
_
Rbp4
_
Stat5b
_
Rbm19
_
Prrt3
_
Cyp26b1
_
Atp6v1e1
_
Fign
_
Bace2
_
Gnao1
_
Slc32a1
_
Stmn1
_
Fstl1
_
Usp48
_
Asb13
_
Kcng4
_
Gpr98
_
Bcan
_
Lhx5
_
Ptcd2
_
Cyhr1
_
Bsg
_
Btg3
_
Gpalpp1
_
Btbd3
_
Nrcam
_
Uqcrc2
_
Calm1
_
Setd7
_
Trh
_
Calm3
_
Mmgt1
_
Ldb3
_
Camk2g
_
Pex14
_
Iqsec1
_
Synj2
_
Ctnnb1
_
Cbx5
_
Gfra1
_
Pfkfb2
_
Oxt
_
Slc6a7
_
Ece2
_
Atp1a3
_
Car7
_
Ccnl2
_
Hdc
_
Ptgs2
_
Bcat1
_
Col5a2
_
Cd1d1
_
St8sia5
_
Gpc5
_
Insrr
_
Ankfn1
_
Plxna3
_
Pdzd8
_
Sorcs2
_
Kcnc3
_
Cdh11
_
Parvb
_
Clcn5
_
Mtss1l
_
Cdh2
_
Lamb1
_
Cdh6
_
Prnp
_
Bpgm
_
Rpn1
_
Cdk5
_
Cdk5r1
_
Stac
_
Cds2
_
Rtn4rl2
_
Itm2c
_
Nsdhl
_
Lhfpl2
_
Slc16a3
_
Vwa1
_
Kazn
_
Ttn
_
Gm12680
_
Astn2
_
Chrm4
_
Slc52a2
_
Chst2
_
Nup93
_
Svil
_
C4b
_
Dhcr24
_
Clu
_
Cnih1
_
Klk8
_
Serpine2
_
Dach1
_
Gnb4
_
Lypla1
_
Nptx2
_
Spint2
_
Fzd6
_
Itpr3
_
Epb4.1l1
_
Col6a1
_
Mgll
_
Crh
_
Crhr1
_
Ackr1
_
Asns
_
Gdpd2
_
Cry2
_
Gstk1
_
Cryzl1
_
Ngf
_
Cxcl12
_
Tsfm
_
Cnr1
_
Twf2
_
Tnrc6a
_
Camk1d
_
Srgap3
_
D16Ertd472e
_
Tomm70a
_
Cd59a
_
Ubxn11
_
Calb2
_
Dbh
_
Ccdc109b
_
Dcn
_
Il16
_
Ddc
_
Ddx3x
_
Zmiz1
_
Pcdh18
_
Stk26
_
Dgkg
_
Dkk3
_
Gpr133
_
Dlx1
_
Cdh2
_
Dlx3
_
Tspan6
_
Drd1
_
Pla2g7
_
Vps39
_
Tfap2c
_
Drd2
_
Clstn1
_
Tmem204
_
Khdrbs3
_
Cpne6
_
Lhfp
_
Timp3
_
Btbd11
_
Pnkd
_
Slc25a10
_
Kcnb1
_
Acsbg1
_
Apol8
_
Csf2rb2
_
Erdr1
_
Tmem14a
_
Efna3
_
Efna5
_
Magi3
_
Slc10a3
_
Lztr1
_
Enc1
_
Entpd1
_
Fam135b
_
Vegfb
_
Tango2
_
Epha7
_
Nxn
_
Eps8l3
_
Ewsr1
_
Cd34
_
Hspa12a
_
Fam213b
_
Cntn1
_
Adamts1
_
Nedd4l
_
Fam134b
_
Hsdl2
_
Sqrdl
_
Ap2b1
_
Zfp114
_
Setd3
_
Ifit3
_
P3h3
_
Npas2
_
Csrnp2
_
Fgf13
_
Glo1
_
Azin1
_
Glul
_
Tex261
_
Pacsin2
_
Podxl2
_
Ogfod1
_
Abhd6
_
Stx3
_
Sybu
_
Pacsin1
_
Ier3
_
Fgfr1
_
Fmo1
_
Gas6
_
Cgnl1
_
Fgfr2
_
Fgfr3
_
Zmym2
_
Tpm3
_
Kcnh8
_
Ociad2
_
Copz2
_
Sorl1
_
Fzd3
_
Dmrt3
_
Fzd8
_
Sema6c
_
Gabrb2
_
Gbx2
_
Gabrg2
_
Slc6a1
_
Gad1
_
Alkbh6
_
Gas5
_
Synj2bp
_
Gata2
_
Gdi2
_
Shoc2
_
Pias4
_
Ghrh
_
Vcam1
_
Glrb
_
Gls
_
Pdzd2
_
Glud1
_
Gnao1
_
Gnas
_
Edc3
_
Gnb2
_
Tmem126a
_
Rasd1
_
Plcd3
_
Grin1
_
Spag16
_
Asb8
_
Grb10
_
Zfand3
_
Ppp2r3d
_
Rspo1
_
Rnf32
_
Ppp1r1b
_
Mc3r
_
Grm1
_
Col15a1
_
Ctbp2
_
Arhgef6
_
Gnb5
_
Ceacam14
_
Hap1
_
Hap1
_
Hars
_
Fyn
_
Hes1
_
Pgm2l1
_
Hgf
_
Rrad
_
Hist1h2bc
_
Hlf
_
Aif1
_
Ybx2
_
Hnrnpdl
_
Ntm
_
Prmt2
_
Hspb7
_
Fam120c
_
Htr1b
_
Ngef
_
Ifnar1
_
9530085C10Rik_
_
Ctf1
_
Igsf5
_
Tsc22d4
_
Impact
_
Isl1
_
Itga3
_
Pde4d
_
Itih3
_
Tac2
_
Jam2
_
Jund
_
Hist2h2aa1
_
Kifc2
_
Kitl
_
Klc2
_
Arhgef5
_
L1cam
_
Flot2
_
Plcb4
_
Lsamp
_
Them6
_
Hmg20a
_
Exosc1
_
Gipc2
_
Lhx1
_
Lhx6
_
Lig1
_
Ptn
_
Mdfi
_
Limk1
_
Ano1
_
Slc17a6
_
B3gat1
_
Ogfrl1
_
Gmppa
_
A830018L16Rik
_
Eya1
_
Lpl
_
Cacng8
_
Slc24a3
_
Lynx1
_
Cst6
_
Panx2
_
Adarb2
_
Rassf6
_
Mcm3ap
_
Mef2c
_
Marcksl1
_
Cyp7b1
_
Mrpl39
_
ND3
_
Cpt1a
_
Mup2
_
Cs
_
Ncam2
_
Ncdn
_
Ncor1
_
Ndn
_
Ndrg3
_
Negr1
_
Neurod6
_
Dlx2
_
Ngfr
_
Rrp1
_
Npdc1
_
Npy
_
Nr2f1
_
Sema5a
_
Nr3c1
_
Nr3c2
_
Hcn2
_
Nr4a2
_
Nr5a1
_
Appl2
_
Nrgn
_
Nrip1
_
Nrtn
_
Cadps2
_
Ybx1
_
Ntrk2
_
Oaz2
_
Lifr
_
Tenm3
_
Tenm4
_
Sebox
_
Adamts17
_
Ogt
_
Olfm1
_
0610007P14Rik
_
Pax6
_
Pax7
_
Pcbp3
_
Pcdhga12
_
Pcp4
_
Ctu1
_
Pcsk1n
_
Isyna1
_
Ggt7
_
Lims2
_
Susd2
_
Pde9a
_
Psmd5
_
Zswim6
_
Pip4k2a
_
Mpnd
_
Rpp25
_
Fam105a
_
Ngfr
_
Per2
_
Pfkl
_
S100a16
_
Pgrmc1
_
Pip4k2c
_
Sdk2
_
LOC434236
_
Pkia
_
B4galt3
_
Pld3
_
A830073O21Rik
_
Dlx1
_
Hoxa5
_
Wnt9a
_
Pcsk1
_
Sp8
_
Plxnc1
_
Nhlh2
_
Pmp22
_
Pnck
_
Trim36
_
Ppap2a
_
Ppp1r7
_
Wwox
_
Prdx1
_
Prkca
_
Hpd
_
Cystm1
_
Thnsl1
_
Npepps
_
Psap
_
Gabra5
_
Ptgds
_
Islr2
_
Lrrc3
_
Ptpru
_
Ptprm
_
Crispld1
_
Ptprt
_
Chst8
_
Pvalb
_
Dnajc16
_
Acot7
_
Pon3
_
Os9
_
Scai
_
Rab1
_
Atp11c
_
Rad23b
_
P2rx1
_
Rbm4
_
Mesdc2
_
Reln
_
Plekhg5
_
Rfx1
_
Veph1
_
Rnf149
_
Rora
_
Rpn2
_
Rtn2
_
Vti1a
_
Rxrg
_
S100b
_
Samsn1
_
Satb1
_
Sbno1
_
Atxn1
_
Scg2
_
Sema3a
_
Exph5
_
Fam120a
_
Sema4a
_
Sema4d
_
Mtch2
_
Sema5a
_
Purb
_
Capzb
_
Sema7a
_
Igsf21
_
Irx2
_
Susd4
_
Sept7
_
Dscaml1
_
Synpo
_
D630014O11Rik
_
C530008M17Rik
_
Slc18a2
_
Tgfb2
_
Fam60a
_
Slc25a3
_
Tmem29
_
Rara
_
Cttn
_
Pcsk5
_
Slit2
_
Arid1b
_
Snap91
_
Snca
_
Slc9a1
_
Clic4
_
Sod1
_
Son
_
Sst
_
Stmn4
_
Hpca
_
Stxbp1
_
Slc35b4
_
Syt1
_
Blcap
_
Syt13
_
Rbbp6
_
Syt2
_
Rasl10a
_
Syt3
_
Syt5
_
Syt6
_
Syt7
_
Matn4
_
Spi1
_
Tbr1
_
Tcf4
_
Tff1
_
Tsc22d1
_
Th
_
Tiam1
_
Tle1
_
Trappc10
_
Tnni3
_
Tm7sf3
_
Tmie
_
Slc36a2
_
Gpx3
_
Trim28
_
Trim9
_
Gosr2
_
Trp53
_
Ttc3
_
Tubb4a
_
E2f4
_
Txnip
_
Scd2
_
Ubash3a
_
Tmprss5
_
Uchl1
_
Slc28a3
_
Ucn3
_
Vamp2
_
Calm2
_
Bche
_
Top1
_
Slc32a1
_
Tbc1d8
_
Dnajb1
_
Vdac3
_
Vipr2
_
Rspo2
_
Vsnl1
_
Pot1b
_
Ubr1
_
Fhdc1
_
Orai2
_
Usp22
_
Adamtsl3
_
Wnt7b
_
Agrn
_
Atp8a2
_
Lppr3
_
Prex1
_
Xbp1
_
Scrn1
_
Yars
_
Tinf2
_
Ywhag
_
Ywhah
_
Ywhaq
_
Rasal1
_
Ywhaz
_
Tmem64
_
Pdzrn3
_
Zfand5
_
Zbtb20
_
Ikzf1
_
Car8
_
Crls1
_
Spon1
_
Rassf3
_
Zfp536
_
Cib2
_
Pck2
_
Tmx2
_
Id2
_
Sbk1
_
Sostdc1
_
Pip5k1c
_
Acsl6
_
Tmem168
_
C130060K24Rik
_
Cbfb
_
Chrna4
_
Cnp
_
Col6a2
_
Lef1
_
Crabp1
_
Hspb8
_
Vwa5a
_
Ctgf
_
Lppr2
_
Ctps
_
Trp53bp1
_
Ctsl
_
Ppp2ca
_
Cyp4f15
_
Bid
_
Cyr61
_
Acat1
_
Mcam
_
Plekhb1
_
Soat2
_
Sh3d19
_
Adamts4
_
Arhgap12
_
E4f1
_
BC034076
_
Ier2
_
Sln
_
Fjx1
_
Tnfrsf19
_
Aox1
_
Gfpt2
_
Ghitm
_
Gng3
_
Gphn
_
Icosl
_
Ctsw
_
Cda
_
Tnip2
_
Gtdc1
_
Akr1c18
_
Echdc2
_
Chst1
_
Zdhhc9
_
Nmb
_
Jup
_
Sun1
_
Upp1
_
Ddit4l
_
Meis2
_
Rasa4
_
Amot
_
Nudt4
_
Tm6sf1
_
Chd7
_
Tnfrsf21
_
Dpy19l3
_
Nfia
_
Slc1a1
_
Ap2s1
_
Bckdhb
_
Asb11
_
Maged2
_
Nr1d2
_
Ifltd1
_
Map3k7cl
_
Glcci1
_
Afap1
_
Gabrr2
_
Stat2
_
Sorbs1
_
Npc2
_
Gjd2
_
Ehd3
_
Pde1b
_
Pde4d
_
Pfn2
_
Plxnb2
_
Plxnb3
_
Prlr
_
Cytip
_
Rasgrf1
_
Rnf11
_
Runx2
_
Slc19a1
_
Atp5a1
_
Sox5
_
Synj1
_
Sesn1
_
Ecel1
_
Cbln2
_
Cdh24
_
Slc20a2
_
Tacr1
_
Tacr3
_
Tmeff1
_
Top2a
_
Trhr
_
Trpc4
_
Aldh5a1
_
Vim
_
Morc3
_
9130024F11Rik
_
Shroom1
_
Zfp260
_
Arhgap26
_
Robo2
_
Calcr
_
Grin2a
_
4932438H23Rik
_
Trpm1
_
Ppp1r9b
_
Alg14
_
Vps18
_
Ccdc85a
_
Aire
_
Atm
_
Ace
_
Cbs
_
Cdh3
_
Copb2
_
Tph2
_
Dpysl4
_
Elf2
_
Esrra
_
Etv6
_
Aktip
_
Gfap
_
Gjb2
_
Tmem2
_
Gmps
_
Grp
_
Pla2g16
_
Itsn1
_
Krtap12-1
_
Gm1125
_
Camk2d
_
Lrig1
_
Lmo1
_
Cyfip1
_
Samd3
_
Msto1
_
Mki67
_
Mmp15
_
Mx1
_
Mpzl1
_
Syncrip
_
Ntf5
_
Jak1
_
Tsc22d3
_
Gpnmb
_
Gmpr
_
Otc
_
Ubash3b
_
LOC434002
_
Ccdc37
_
Akap13
_
Sez6l2
_
Sema3d
_
Egr3
_
Cnnm2
_
Rbm11
_
Etfa
_
Arhgap17
_
Znrf1
_
Chrna5
_
Chrna9
_
Shh
_
Slc6a9
_
1110007C09Rik
_
Sox9
_
Tcf7l1
_
Ovgp1
_
Pltp
_
Hpcal1
_
Net1
_
Sh3gl2
_
Tsc2
_
Ptchd4
_
Ucn
_
Park7
_
Vil1
_
Mettl24
_
Wnt4
_
Adamts8
_
Kcnj2
_
Pmfbp1
_
Akap2
_
Dlx5
_
Scg5
_
C1ra
_
Allc
_
Thrsp
_
Cyp39a1
_
LOC277860
_
Lrrc1
_
Ifngr2
_
Tm7sf3
_
Prune2
_
Rap2a
_
Zfp788
_
AI606473
_
Ostn
_
Palm2
_
Palmd
_
Fam84a
_
Cdkn2b
_
Pcdhb18
_
Ptgds
_
Pex5l
_
Plekhh2
_
Slc2a12
_
Slc8a2
_
Sh3rf1
_
Dmbx1
_
Dok4
_
E130016E03Rik
_
Ckb
_
Lamp5
_
Ephb3
_
Pqlc1
_
Etv3
_
Dusp14
_
Foxd3
_
Cyb5r1
_
Gpr35
_
Fzd5
_
Slc17a7
_
Prkcq
_
Hes6
_
Kif15
_
Dgki
_
Lmx1b
_
Aacs
_
Mtf2
_
Myb
_
Ufsp1
_
Ndel1
_
Shb
_
Pdgfa
_
Pla2g12a
_
Pqbp1
_
Cblb
_
Prrx2
_
Gdf1
_
Dennd5a
_
Ifit2
_
Dhrs7
_
Ddt
_
Slc17a7
_
Chd2
_
Setbp1
_
Tbc1d1
_
Sprr1a
_
Srprb
_
Suv39h2
_
Chrd
_
Tmem25
_
Ube2g2
_
Chrna3
_
Trappc1
_
Frat2
_
Nfib
_
1110037F02Rik
_
Plxdc2
_
Plxnb1
_
Pou2f1
_
Rgs16
_
Rgs4
_
Sh3bgr
_
Slc20a1
_
Txn2
_
Abhd11
_
Wif1
_
Adap1
_
Fgfr4
_
Gpr84
_
Grin1
_
Rwdd2b
_
Gm520
_
1200016E24Rik
_
Srpr
_
Serpinb1c
_
Slc36a4
_
Asb6
_
Bcl7b
_
Cdkn2d
_
Cx3cr1
_
Zfp423
_
Fmnl1
_
Foxb2
_
Gba
_
Slc27a4
_
Gcgr
_
Gli3
_
Gpc4
_
Gpr108
_
Hoxd9
_
Ptpn18
_
Kctd5
_
Cenpf
_
Sh2b3
_
Letmd1
_
Ufc1
_
Cd24a
_
Slc25a22
_
1700086L19Rik
_
Slc39a5
_
Sppl2a
_
Ube2b
_
Ostc
_
Cmbl
_
Srfbp1
_
Ppp2r2b
_
Gpt2
_
Abtb1
_
Aplnr
_
Tsc22d2
_
Mcl1
_
Vash2
_
Npffr2
_
Brd8
_
Spg21
_
Ankrd54
_
Cacnb4
_
Casp9
_
Cdkn2c
_
Chuk
_
Myt1
_
Copa
_
Cpe
_
Prdx4
_
Tomm20
_
Prr15l
_
Hdlbp
_
Nomo1
_
Cxcl13
_
Cnr1
_
Cwh43
_
Rpl23
_
Eef1g
_
Fn3krp
_
Aldh2
_
Ttc27
_
Parp8
_
Ndufs8
_
Nudt2
_
Erbb2
_
Fam210a
_
F2r
_
Wdr47
_
Cd4
_
Tmem47
_
Adm
_
Plcl1
_
Folr2
_
Casq1
_
Sh3rf2
_
Il13ra1
_
Dffa
_
Hs6st1
_
Col18a1
_
Sms
_
Gmfb
_
Gnaz
_
Eya4
_
Gng11
_
Gng7
_
Gpc6
_
Il17rd
_
Gpr85
_
Polr2m
_
Itgav
_
Dtd1
_
Tox3
_
Hnf4a
_
Palm3
_
Homer1
_
Ikbkb
_
Med26
_
Kcnab2
_
Xk
_
Kcnc4
_
Filip1l
_
Kcne2
_
Kcnip1
_
Kcnk13
_
Kcnv2
_
Myadm
_
Mon1b
_
Esr2
_
Lphn2
_
Mlc1
_
Mmaa
_
Bai3
_
Firre
_
Mx2
_
Nat6
_
Ncoa4
_
Ngfrap1
_
Slc22a13
_
Notch3
_
Agps
_
Gabbr2
_
Ppp2r5b
_
Lrrn2
_
Vezt
_
Grik2
_
Dach2
_
Wash
_
Setd4
_
P2ry6
_
Pcbd1
_
Pde1c
_
8030498B09Rik
_
Mxi1
_
Arl2
_
Dad1
_
Pigt
_
Rap1b
_
Plcd1
_
Pxn
_
Raf1
_
Ramp3
_
Htr5b
_
Rgs3
_
Zfp280c
_
Slc2a13
_
Extl2
_
Sfrp2
_
Ndufv3
_
Siglece
_
Stx12
_
Six1
_
Slc12a8
_
Slc29a1
_
Slc2a3
_
Slc34a1
_
Slc38a1
_
AI852640
_
Slc39a14
_
Ankrd6
_
Egr1
_
Slc6a6
_
Slc9a8
_
Snap25
_
Nat8l
_
Syap1
_
Clptm1l
_
Adk
_
Galm
_
Dbn1
_
Cast
_
Syt13
_
Zbtb16
_
Hmox2
_
Tbl2
_
Tcea3
_
Tcerg1
_
Hnf1b
_
Hdac7
_
Fam60a
_
Pllp
_
Crlf2
_
Ubl3
_
Usp16
_
Vdac2
_
Hspb8
_
Tmem176b
_
Zfp191
_
Phldb2
_
Fcrls
_
Fam89b
_
Car4
_
Wnt2b
_
Ptgfr
_
Pcdhb17
_
Dusp3
_
Pdgfd
_
Kcnj6
_
Igfbp6
_
Slc38a3
_
Tmc6
_
Tspan11
_
Rxrb
_
Scnn1g
_
Sema3e
_
Cap2
_
Slc4a10
_
Fam213a
_
Spp1
_
Hdac6
_
Rsrp1
_
Mrpl54
_
Efcab6
_
Nipal3
_
Chrnb3
_
Mlxipl
_
Tmem132a
_
Wbscr17
_
Alcam
_
Wnt7a
_
Cnp
_
Obfc1
_
Lrtm2
_
Ap2a2
_
Tsen34
_
Dio3
_
Micu2
_
Grina
_
Uba6
_
Tcf4
_
Ccdc184
_
Acvrl1
_
Smg9
_
Satb1
_
Pea15a
_
Rtn1
_
Mad1l1
_
Ccni
_
Tmem218
_
Dbnl
_
Sik3
_
Igsf5
_
Fam136a
_
Trap1
_
Snhg7
_
Rabep2
_
Fam26e
_
Smarca2
_
Igfbpl1
_
Enpep
_
Adcy7
_
Ccdc137
_
Osbpl3
_
Olfml2b
_
Rcor1
_
Klhl24
_
Mterf2
_
Prph
_
C2cd2
_
Ank2
_
Soga3
_
Aph1a
_
Strip2
_
Galnt18
_
Kcnj6
_
Pde4dip
_
Fam195a
_
Doc2b
_
Rfx7
_
Scnn1a
_
Dlx6os1
_
Pea15a
_
Arg2
_
Enox2
_
Cplx3
_
9830166N24Rik_
_
Ankrd33
_
Ldlrap1
_
Ppp1r3b
_
Abhd8
_
Atp5o
_
Adcy7
_
Kcna6
_
Adrm1
_
Agtr1a
_
Slit3
_
Clrn3
_
Samd11
_
Aldh1a2
_
Ankrd10
_
Anxa11
_
Apba2
_
Rcan2
_
Apbb1
_
Slc8a1
_
Aqp9
_
Gadd45b
_
Eps8
_
Ddx5
_
Arhgef2
_
Arntl
_
Asgr1
_
Anxa2
_
Ssbp2
_
Ciao1
_
Axin1
_
B4galt6
_
Barx2
_
Boc
_
Gpr146
_
Tacc3
_
Kcne4
_
Bmp7
_
Mpc1
_
Btd
_
Tspo
_
Sdccag3
_
Cbr3
_
Cct8
_
Dlst
_
Cdh4
_
Cdkn1a
_
Col6a3
_
Slc26a10
_
Cer1
_
Chga
_
Usp46
_
Chrm3
_
Chrnb2
_
Cnot7
_
Cmtm4
_
Mylk
_
Mocs1
_
Cldn12
_
Cnih2
_
Ssb
_
4833420G17Rik
_
Fam212b
_
Gpx2
_
Eps8l1
_
Crat
_
Atp1b2
_
Cr1l
_
Cryaa
_
Csrp1
_
Cstf2
_
Hrh2
_
Cx3cl1
_
Recql4
_
Dbr1
_
Eif4a3
_
Drd4
_
Gbas
_
Dip2a
_
Dkk1
_
Smu1
_
Nubp1
_
Slx1b
_
Aldh1l1
_
Clta
_
Dok2
_
Blnk
_
Donson
_
Fxyd5
_
Stmn2
_
Tgfbr2
_
Dph2
_
Pigp
_
Dtx1
_
E2f1
_
E2f6
_
Efemp2
_
Ei24
_
Eif2ak3
_
Eif5a
_
Elk1
_
Epha4
_
Ephb6
_
Mtmr7
_
Eps15l1
_
Dlgap1
_
Erf
_
Grid2
_
Pcp2
_
Neurl1a
_
Fbln1
_
Fbrs
_
Gpr162
_
Fbxo2
_
Golga7b
_
Fem1a
_
Tmem179
_
Zscan22
_
LOC381076
_
Fhl1
_
Fkbp10
_
Fkbp6
_
Flt1
_
Fnta
_
9330182L06Rik
_
Foxg1
_
Foxi1
_
A230065H16Rik
_
Frs3
_
Adra2a
_
Parm1
_
Fzd4
_
Cd63
_
Gabarap
_
Gabrb1
_
Cacng3
_
Anxa1
_
Ivns1abp
_
Gas1
_
Gja1
_
Gjb5
_
Spint1
_
Iqgap2
_
Gnai2
_
Gnat1
_
Gnpnat1
_
Meis1
_
Tfam
_
Gpr37
_
Nwd2
_
Akr1b3
_
Necab3
_
Kctd9
_
Parvg
_
Tppp3
_
Dusp10
_
Mafb
_
Tmem176a
_
Nudt21
_
Hdgf
_
Hes3
_
Hes7
_
Higd1a
_
Baiap2
_
Hip1
_
Hmga1
_
Hoxb13
_
Hax1
_
Hs3st1
_
Hunk
_
Zfp358
_
Atp6v1d
_
Igf2
_
Il11ra1
_
Il17rb
_
Il17rc
_
Impg1
_
Focad
_
Irak1
_
Irx1
_
Rxfp2
_
Hsf1
_
Kdm4b
_
Fbxo34
_
Jun
_
Junb
_
Kcne3
_
Kcnj15
_
Kcnj16
_
Kcnj8
_
Kdr
_
Stim2
_
Kif5b
_
Fgf15
_
Kpna4
_
Kalrn
_
Dhrs3
_
Aff4
_
Lat
_
Ldb3
_
Gjb6
_
P3h1
_
Leprotl1
_
Lhx2
_
Lhx4
_
Lhx8
_
Lmo2
_
Lmo4
_
Madcam1
_
Pam16
_
Map3k7
_
Tnfrsf1a
_
Mapk13
_
Cdh13
_
Mapk14
_
Mapre1
_
Mcm5
_
Ldlr
_
Mif
_
Mtf1
_
Fat3
_
Mtpn
_
Mtrr
_
Clvs2
_
Kat8
_
Gm953
_
Ndufs1
_
Nfix
_
Nfkbie
_
Nkiras2
_
Vim
_
Nr2e3
_
Calca
_
Nrf1
_
Eepd1
_
Bdnf
_
Nudc
_
Nudt9
_
Sod1
_
Olig2
_
Omg
_
Optn
_
Otp
_
Tubb2a
_
Pafah1b1
_
Park2
_
Pax3
_
Mtss1
_
Pax9
_
Btg3
_
Pcdhb20
_
Masp1
_
Szt2
_
Pde4b
_
Pdk3
_
Pdlim2
_
Pgrmc1
_
Ctnnbip1
_
Phox2b
_
Nr4a3
_
Pkig
_
Plg
_
Plk1
_
Gpc3
_
Pnrc2
_
Pgr15l
_
BC100451
_
Krt80
_
Pparg
_
Ppm1a
_
Ppm1g
_
Pnrc1
_
Psmd4
_
Ptn
_
Fabp3
_
Pvrl4
_
Vopp1
_
Pycrl
_
Ran
_
Rangap1
_
Ppp2r5d
_
Rela
_
Id3
_
Relb
_
Traf7
_
Rgs14
_
Sned1
_
Fbln7
_
Rhbdf1
_
Rbm38
_
Rora
_
Gpr146
_
Rorb
_
Ryk
_
Rasal2
_
Ttbk2
_
Nrn1
_
Igsf9b
_
Nos1
_
P3h4
_
Kcnk2
_
Kcnc2
_
Kcnd3
_
Scarb1
_
Col5a1
_
Cdk8
_
Gpr6
_
Frrs1
_
Serpinf1
_
Nr4a1
_
Trpv4
_
Satb2
_
Tmem178
_
Plekha7
_
B3gnt5
_
Endod1
_
Sla
_
Fnbp1l
_
Slc11a2
_
Cdr2
_
Strada
_
Cntn4
_
Fat3
_
Dlgap2
_
Slc16a14
_
Slc16a4
_
Ank1
_
Slc16a6
_
Gpr165
_
Slc18a1
_
Slc19a2
_
Slc25a1
_
Slc25a5
_
Npsr1
_
Bdh2
_
Slc26a7
_
Slc26a8
_
Slc2a4
_
St6galnac5
_
Slc31a1
_
Slc35a1
_
Slc35a2
_
Dgkb
_
Slc35a3
_
Slc35b2
_
Slc35c2
_
Slc38a4
_
F5
_
Slc5a1
_
Ank3
_
Slco4a1
_
Srp54a
_
Ssr1
_
Brs3
_
Rfxank
_
Rplp0
_
Stat3
_
Stc1
_
Smpd1
_
Clns1a
_
Gpr89
_
Aurka
_
Cacnb3
_
Cacng5
_
Cckbr
_
Tsg101
_
Syt11
_
Grm4
_
Sytl3
_
Taf13
_
Tat
_
Tcf19
_
Tfap2a
_
Tmbim6
_
Tspan32
_
Scg3
_
Necab2
_
Tgfb3
_
Ssrp1
_
Brinp3
_
Thpo
_
Med24
_
Tle3
_
Lrrc49
_
Neurod1
_
Tnfrsf12a
_
Nts
_
Lgi2
_
Traf2
_
Traf5
_
Prss23
_
Mob3b
_
Atf3
_
Myc
_
Otof
_
Otof
_
Nprl2
_
Tyr
_
Dusp18
_
Slc5a7
_
Unc13c
_
Cubn
_
Six3os1
_
Fdft1
_
Usp25
_
Ezr
_
Dcaf4
_
St8sia2
_
Esrrg
_
Hspa1l
_
Il10ra
_
Mgat5b
_
Lmo3
_
Wnt11
_
Wnt3
_
Insig1
_
Lrfn2
_
Wnt6
_
Ywhab
_
Ywhae
_
Zbtb18
_
Lrrc4
_
Lrrn3
_
Ltn1
_
Gltscr1l
_
Slc30a3
_
Bhlhe40
_
Ndst3
_
Slit1
_
Fndc1
_
Gas7
_
Grem2
_
Gucy2c
_
Hc
_
Slc36a1
_
Rasgrf2
_
Scml2
_
Slitrk6
_
Smug1
_
Opcml
_
Bsn
_
C230030N03Rik
_
Tmem72
_
Arf1
_
Clec11a
_
Mef2a
_
Tnfrsf11a
_
Cnnm1
_
Cntnap1
_
Col4a5
_
Cox4i1
_
Prune2
_
Tcerg1l
_
Tnfrsf25
_
Cyld
_
Slc44a1
_
Mroh1
_
Lppr4
_
Mylk3
_
Ablim3
_
Dcbld2
_
Dgkh
_
Snhg11
_
Eif5a2
_
Eml1
_
Kcnip4
_
Rgag1
_
Ryr1
_
Rapgef6
_
Tdo2
_
Csdc2
_
Rfx3
_
Socs6
_
Trpc5
_
Prkcb
_
E130012A19Rik
_
Lonrf3
_
Htr2c
_
Scand1
_
Dock6
_
Nipa2
_
C1qtnf3
_
Gtf2h4
_
Gtf2h2
_
Gnrh1
_
Ripply3
_
LOC433254
_
Cirh1a
_
Glra1
_
Kcnc1
_
Mex3d
_
Pyurf
_
Kcna1
_
Atat1
_
Plcd3
_
Ctnna2
_
Ptprj
_
Rufy4
_
A130014H13Rik
_
Dscaml1
_
Iyd
_
Fat4
_
Dock10
_
Hspb1
_
Qrfp
_
Trip4
_
Bag2
_
Pcgf6
_
Spock3
_
Hkdc1
_
Cyp27a1
_
Rcsd1
_
Eef1b2
_
Pcdh1
_
Rnasek
_
Pvrl3
_
Tppp
_
Cish
_
Ap2b1
_
Gm5607
_
3110035E14Rik
_
Med30
_
Hsd11b1
_
Thbs4
_
BC005537
_
Serpina3k
_
Myl4
_
Zbtb20
_
Tmem106b
_
Stk32a
_
Phactr1
_
Itgb5
_
Mybpc1
_
Rftn1
_
Kirrel3
_
Wscd1
_
Sertm1
_
Sertad4
_
Mpp3
_
Sobp
_
Fn1
_
Tanc1
_
Syngr3
_
Dkkl1
_
Fabp5
_
Parva
_
Synpr
_
Ublcp1
_
Gadd45g
_
Gpr125
_
Fam46a
_
Hapln1
_
Llgl1
_
Lgals1
_
Fam50a
_
Ccdc171
_
C1ra
_
S1pr5
_
Fgfr1
_
Depdc7
_
Dtnbp1
_
Aldob
_
Cdca2
_
Gpr137
_
Igsf3
_
Chgb
_
Cd97
_
Fgfr1op2
_
Ube3c
_
Bhlhb9
_
Gprasp1
_
Layn
_
Tmem131
_
Dbp
_
Tex40
_
Ina
_
Uap1
_
Calml3
_
Sc5d
_
Cdh13
_
Gng4
_
Hpcal4
_
Map3k15
_
Map3k15
_
Sparc
_
2010300C02Rik
_
Zfp189
_
Sostdc1
_
Bcar3
_
Flcn
_
Adrb1
_
LOC433228
_
Ubr3
_
Hspb3
_
3830406C13Rik
_
Cnppd1
_
Ndrg2
_
Dbh
_
Nod2
_
Glra3
_
Itga7
_
Kit
_
Apoe
_
Pfkp
_
Nfil3
_
Clu
_
Gpr139
_
Kcnj2
_
Kctd12
_
Cldn5
_
Camk2a
_
Odc1
_
Eid1
_
Prkar2b
_
Ppp1r2
_
Sstr4
_
Elmo1
_
Slc4a2
_
Grik1
_
Crhr2
_
Cxcl12
_
Bend5
_
Dyrk1a
_
Ndufs2
_
Vav2
_
Hist3h2a
_
Sirpa
_
Rxfp1
_
Lrsam1
_
Rhobtb3
_
Pik3r3
_
Adamts15
_
Bcl11a
_
Ttc39b
_
Arhgap33
_
Ccdc91
_
Adipor1
_
Scn4b
_
Zfhx4
_
Gm6740
_
Thbs4
_
LOC432748
_
Tiam2
_
Slc43a2
_
Cdo1
_
Foxo1
_
Foxp1
_
Plagl1
_
Ube2h
_
Gng2
_
Pou6f2
_
Rfx4
_
Thrb
_
Brk1
_
Glt8d2
_
Ttc9b
_
Megf10
_
Ube2ql1
_
Limch1
_
Cyp51
_
Capns1
_
Ank
_
Rps4l
_
Rap2b
_
Zfyve28
_
Rpl32
_
4921531P07Rik
_
Gm1441
_
Ramp1
_
Chrm5
_
Hba-a1
_
Rnf128
_
Gxylt2
_
Ptpn3
_
Npy1r
_
LOC433311
_
Arl5a
_
Rgs9
_
Pcdhga5
_
Gpr83
_
Casr
_
Sim1
_
Calcb
_
Rab3b
_
Clic5
_
Tekt1
_
Pard3
_
Ccdc3
_
Lpin1
_
Lamc1
_
Akr1e1
_
Chchd2
_
Map2
_
Draxin
_
Rrm2
_
Hist1h1b
_
Cxcl14
_
Cdhr3
_
Srgap1
_
Slc7a14
_
Ilf3
_
Stra6
_
4933427D14Rik
_
Tmem91
_
Best2
_
Stard5
_
Adarb1
_
Ntm
_
Sh3kbp1
_
Ighm
_
Klk10
_
Pknox1
_
Atp1a1
_
Zfpm2
_
Gap43
_
Wdfy2
_
Dync1li1
_
Adcy2
_
Adora2a
_
Cd81
_
Caml
_
Elovl6
_
Rnf13
_
Fam84b
_
Zbtb48
_
Gpd2
_
Dlg4
_
4930506M07Rik
_
Iffo2
_
Pde6g
_
Skiv2l2
_
Brinp1
_
Eva1c
_
Ace
_
Dpy19l1
_
Icmt
_
Timm10
_
Axin2
_
A230097P14Rik_
_
Fam46a
_
A530057D15Rik_
_
Cdkl5
_
Nrxn3
_
Abcc4
_
Rims3
_
Fam196a
_
Fam196a
_
B830028H17Rik_
_
Cacna2d1
_
Adra1a
_
Dok1
_
Myo5c
_
Gbe1
_
Hsp90b1
_
Acot13
_
Tomm34
_
Glg1
_
Rdh5
_
Lrrc16a
_
Elmod3
_
Wrap73
_
Nkd2
_
Tk2
_
Qdpr
_
Pdia5
_
Trp53i11
_
Cth
_
Pttg1ip
_
Acsl3
_
Arid5a
_
Nop10
_
Lcat
_
Scara3
_
Anxa5
_
Syne1
_
Syne2
_
Synpo
_
Epha1
_
Gucy2f
_
Mm.26272
_
Zfyve9
_
Rora
_
Bcap29
_
A830036E02Rik
_
B3gnt2
_
Slc13a3
_
Syt16
_
Col9a3
_
Fbxw2
_
Usp11
_
Vps35
_
3632451O06Rik
_
Pls3
_
Ncaph2
_
Fh1
_
Dag1
_
Chst2
_
Sulf1
_
Phyhipl
_
Serpinb1b
_
Phyhip
_
Limk2
_
5330421K23Rik_
_
Kcnab1
_
Lnx2
_
Nt5dc3
_
Avpr1b
_
Sqle
_
Tead1
_
Gal
_
Fgf1
_
Atp9b
_
Clic6
_
Snx31
_
Cdh11
_
Arpp21
_
Tceb3
_
Lypd1
_
Hcrtr1
_
P4hb
_
Nrxn3
_
Fam114a2
_
Cidea
_
Dpp10
_
Crym
_
Celf4
_
Cdh10
_
Igfbp5
_
Pthlh
_
C2cd2l
_
Dhx40
_
Fxyd6
_
Dgcr6
_
Fxyd7
_
Tmem65
_
Myo5b
_
Trpc2
_
Col4a2
_
Fez2
_
Gdf11
_
Nrp1
_
Ppfibp1
_
Impg2
_
Gsta4
_
Hectd2
_
Cct2
_
Rbm18
_
Serpinb6a
_
Mylip
_
Abcd1
_
Homer3
_
Pias2
_
Nxf1
_
Gaa
_
Nsf
_
Serinc5
_
Rec8
_
Rps5
_
Slc9a7
_
Cyp2j6
_
Slc25a25
_
Pdgfrb
_
Gca
_
Gpm6b
_
Mm.86217
_
Osbpl8
_
Stk33
_
Tmem63c
_
Gabrb2
_
Casq2
_
Ckm
_
Got2
_
Hadhb
_
Fam49a
_
Lix1
_
Ipo5
_
Ltbp3
_
Map1a
_
Nin
_
Tac2
_
Ntrk3
_
Psme1
_
Prdx5
_
Prosc
_
Rasgrp1
_
Rasgrp2
_
Slc9a6
_
H2-Eb1
_
Zfp330
_
Ar
_
Map7d2
_
Dnm3
_
Cap1
_
Adam19
_
Adamts18
_
Rassf8
_
Mical2
_
Ism1
_
Krt222
_
Lrp5
_
Ppp4r4
_
Dsel
_
Arsj
_
9530091C08Rik
_
9630033F20Rik
_
Ptchd1
_
Zmat4
_
Pop4
_
Eif3j1
_
Avp
_
Pde8b
_
Stt3b
_
Map4k3
_
Tdg
_
Stk17b
_
Fzd4
_
Rpl8
_
Nacc2
_
Mapre2
_
Cntnap4
_
Serinc1
_
Nfix
_
Gm5433
_
Iscu
_
Kcnd2
_
Opn3
_
Slc38a1
_
Pou4f1
_
Enpp2
_
Glce
_
Cox6c
_
Zfhx3
_
Hmx3
_
Kcnh3
_
Ldb2
_
Ak4
_
Gpr17
_
Rab27b
_
Atp6v1a
_
Avpr1a
_
Aars
_
Htr3b
_
Pcyt1b
_
Ctnnd1
_
Cdyl2
_
Gpr156
_
Ttpal
_
Celf3
_
Impa1
_
Cited1
_
Lyz1
_
Tmem33
_
Clk2
_
Phf1
_
Limk1
_
Dirc2
_
Hint1
_
Cmklr1
_
Mecp2
_
Tmc1
_
D11Wsu47e
_
Notch3
_
Mtif2
_
Tenm3
_
Tnnt3
_
Gnb1
_
Arhgap39
_
Zfp804a
_
Snca
_
Lpar1
_
Ogfr
_
Rorb
_
Tnc
_
Efnb3
_
Kif5a
_
Dkk3
_
Pitx2
_
Map2k5
_
Rer1
_
Galp
_
Eif2d
_
Atp5s
_
Ddc
_
Zak
_
Dynlrb1
_
Sdccag8
_
Cux1
_
Uqcrb
_
Dctn6
_
Reln
_
Cck
_
Arhgdig
_
Mc4r
_
Mvp
_
Syt12
_
Pi4k2a
_
Ctgf
_
Aldoc
_
Pus7
_
Thrsp
_
Cuedc2
_
Gja1
_
Cd44
_
Lrp5
_
Grm2
_
Col6a1
_
Sox10
_
Pvr
_
Entpd2
_
Isoc1
_
Frzb
_
Trim3
_
Wnt5a
_
Cd8b1
_
Fam81a
_
Notum
_
P4ha2
_
LOC545854
_
St3gal6
_
Tspo
_
Calb1
_
Uchl1
_
Rps12
_
AI450948
_
Snd1
_
Usp11
_
Ajap1
_
Ldlrad4
_
Nek7
_
Vps35
_
Pdxdc1
_
Trap1
_
Bmp4
_
Plp1
_
Gad1
_
Cables2
_
Htr2b
_
Tcf7l2
_
Sp3
_
Ngb
_
Sssca1
_
Hopx
_
Bhlhe22
_
Aqp4
_
Arhgef28
_
Rgs6
_
Scube1
_
Gria3
_
Gdap2
_
Pvalb
_
Thbs3
_
Slc22a8
_
Clptm1
_
Tecr
_
Chst14
_
Tspan5
_
Tll1
_
Sub1
_
Chst10
_
Wls
_
Cops7b
_
Coro2b
_
Schip1
_
Itpka
_
Grik1
_
Col27a1
_
Col5a3
_
Iqcj
_
Nenf
_
Igfbp3
_
Atic
_
Dgat2
_
Pfkfb3
_
Msh2
_
Papl
_
Fryl
_
Ephb1
_
Cartpt
_
Fam222a
_
N6amt1
_
Tcf3
_
Flot1
_
Tacr1
_
Kcnk1
_
Vtn
_
Mapt
_
Apln
_
Slc9a3r2
_
Ralb
_
Mtmr12
_
Fbln2
_
Apba1
_
Tmem175
_
Hapln4
_
Irs4
_
Kifc3
_
Mest
_
Pde3a
_
Pdyn
_
Arl10
_
Sox2ot
_
Cdh18
_
Sun2
_
B3gat2
_
Dnajc6
_
Rprm
_
Agap3
_
Cdh15
_
Gabra3
_
Mgp
_
Gpr161
_
Efna1
_
Atp13a5
_
Gpr155
_
Col14a1
_
Gpr26
_
Dnal4
_
Pstpip1
_
Rab3ip
_
Rasl11b
_
Col19a1
_
Zim2
_
Cox6a2
_
Sgcd
_
Tgfb1
_
Ccser1
_
6430548M08Rik
_
Dstn
_
E330009J07Rik
_
Megf9
_
Exosc8
_
Fscn1
_
Nxpe3
_
Stc2
_
Mela
_
Mmp14
_
Msn
_
Kiss1r
_
Mtmr6
_
Nt5m
_
Kifc5b
_
Me2
_
Gldc
_
A4galt
_
Plek
_
Edc3
_
Cmtm3
_
Pxylp1
_
Kcnh7
_
Plxna4
_
Fgf12
_
Irf9
_
Csnk1a1
_
Ccdc92
_
Stard9
_
Atp11a
_
Kcnq3
_
Fstl4
_
Trps1
_
Tnfaip3
_
S1pr1
_
Arl3
_
Tmem11
_
Cyb5a
_
Bag1
_
7420498E04Rik_
_
Wbp5
_
Trpc6
_
Rnf152
_
Srsf9
_
Cd33
_
Rbmx
_
Serpina3n
_
Shisa2
_
Rdh14
_
Prkacb
_
Rb1cc1
_
Chrna2
_
Sdhb
_
Pitrm1
_
Elovl5
_
Hexim1
_
Arc
_
Cnksr3
_
Dok5
_
Rarres1
_
Ankrd24
_
Acat2
_
Serpina1e
_
P2rx6
_
Prss12
_
Cyr61
_
Serpinb2
_
Slc35f1
_
Serinc1
_
Cep78
_
Cplx1
_
Matr3
_
Chrna1
_
Cacna2d3
_
Pomgnt2
_
Slc35f3
_
Ndufa10
_
Ipo4
_
Sptlc2
_
A530079E22Rik
_
Sowaha
_
Acvr1c
_
Styk1
_
Crtc1
_
Amigo2
_
Fbxl5
_
AI593442
_
Gabrb3
_
Suco
_
Aim1
_
Rph3a
_
Tnr
_
Gabrr1
_
Pdha1
_
Acyp2
_
Cpt1c
_
Lepr
_
Siae
_
Wnt1
_
Fras1
_
Lum
_
Gpc1
_
Grsf1
_
Ids
_
Il1rapl2
_
Qrfpr
_
Galnt16
_
Micu1
_
Exoc4
_
Pex13
_
St6gal1
_
Triap1
_
Bcl2l11
_
Eps8l2
_
Ssr2
_
Shisa9
_
Neil3
_
B230312C02Rik
_
Fam160a1
_
Ttll6
_
6820408C15Rik
_
Zfhx2os
_
Arl15
_
Foxred2
_
Irs2
_
LOC433727
_
Zdhhc23
_
Ccdc88c
_
Pth1r
_
Fam184a
_
Car10
_
Ltb
_
Pvrl1
_
Sall3
_
Ckap2
_
N4bp2
_
Lyst
_
Lzts1
_
Mal2
_
Map3k5
_
Mapk4
_
Atxn10
_
Rgs8
_
Yme1l1
_
Psmc2
_
Nxph3
_
Zfp790
_
Zfp810
_
Ptbp1
_
Gde1
_
Ap1b1
_
Ppp6r3
_
Prcp
_
Sgsm3
_
Camkk1
_
Rasgef1b
_
Gss
_
Sod3
_
Jdp2
_
Mlip
_
Itfg1
_
Fezf2
_
Cst3
_
Nrsn1
_
Ly6e
_
Wnt7b
_
Ola1
_
Ptprcap
_
Oprk1
_
Maob
_
Kcng3
_
Slc35f2
_
Pde1a
_
Ntsr1
_
Magi1
_
Fam96b
_
Ube2i
_
Dbndd2
_
Cacng7
_
Cdc42ep3
_
Ninj1
_
Man1a
_
Dmwd
_
Nov
_
Fam20a
_
Ccng1
_
1700037H04Rik
_
Dusp1
_
Pdcl
_
Ccdc136
_
Bzw1
_
Pdxk
_
Pelo
_
Kank2
_
Sart3
_
Slc5a3
_
Lyve1
_
Grin2c
_
Per1
_
Lxn
_
Ptpro
_
Lgals1
_
Spock1
_
Elavl2
_
1810041L15Rik
_
Lhpp
_
Chrnb1
_
Gabra1
_
Kcnj3
_
Neto2
_
Rreb1
_
Nqo2
_
Esyt1
_
Ergic1
_
Sipa1l2
_
Ero1lb
_
Neu2
_
Nhlrc1
_
Nid1
_
Ugp2
_
Nog
_
Nrk
_
Nxph1
_
Slco1a5
_
Prpf6
_
Gpr56
_
Tgif1
_
Tbp
_
Plxna2
_
Inhba
_
Epb4.1l2
_
Mtus2
_
Pcsk6
_
Kif26b
_
Dyrk1b
_
Pvr
_
Csk
_
Ap1b1
_
Pcdhb16
_
Kctd17
_
Pak6
_
Fam189a1
_
6330403A02Rik
_
Smoc1
_
Sar1a
_
Gm10635
_
Foxa1
_
Grik5
_
Hectd1
_
Chrna6
_
Atp6v0a2
_
Paip2
_
Grin3b
_
Ing4
_
Chrng
_
Gabra6
_
Cd47
_
Gabrg1
_
Coch
_
Cyc1
_
A830039N20Rik
_
P2rx2
_
P2rx4
_
P2rx5
_
Glra4
_
P2rx7
_
Grik4
_
Grin2d
_
Prkca
_
Lhcgr
_
Vstm2b
_
A930009E05Rik
_
2010111I01Rik
_
Prlr
_
Ptprt
_
Calb1
_
Amph
_
Avil
_
Nek2
_
AW046396
_
Syt4
_
Heg1
_
Acan
_
Arxes1
_
Tgfb2
_
Plxdc1
_
Nrm
_
Efr3a
_
Apba1
_
Usp14
_
Paip2
_
Pop4
_
Gaa
_
Cdh13
_
Inpp5j
_
Prkcq
_
Relt
_
B4galt5
_
Nr2e3
_
Sh3bgrl2
_
Baz1a
_
Chn2
_
Cers4
_
Fancd2
_
Pcp2
_
Pmaip1
_
Disp2
_
Tmem229b
_
Thbs2
_
Trp73
_
Rln3
_
Rragd
_
Sez6l
_
Sfmbt2
_
St8sia3
_
St8sia6
_
Slc16a2
_
Rcn1
_
Klf7
_
Fam131b
_
Slc35d3
_
Pola1
_
Slc6a15
_
Ets1
_
Celf6
_
Thsd7b
_
Smpd4
_
Plcxd2
_
LOC433093
_
Mfap1a
_
Polr1e
_
Tnni1
_
Sh3bp4
_
Prkcz
_
Eif1b
_
Arl6ip5
_
Man2b1
_
Fbxo9
_
Bmp3
_
Ptpdc1
_
Mapk8
_
Tnfaip8l3
_
Acan
_
Nrgn
_
Pde4a
_
Iars2
_
Chat
_
Lrrc38
_
Sulf2
_
Kctd16
_
Cacybp
_
L1cam
_
Actr1a
_
Fam43a
_
Ptprd
_
Col16a1
_
Sez6
_
Slc25a36
_
Vstm2a
_
Mcm6
_
Vsx2
_
Smtn
_
Rest
_
Pdgfra
_
Ccp110
_
Bend6
_
Mfsd2a
_
Arhgef10
_
Sox2
_
Olfml3
_
Arpc1a
_
Ugdh
_
Taok1
_
Mbp
_
Slc25a27
_
Sema6a
_
Far2
_
Cerk
_
Rcan1
_
Anxa4
_
Psd3
_
Nol4
_
Nfyc
_
Fzd1
_
Sytl4
_
Mycl
_
Ntf3
_
Kpna1
_
Cotl1
_
Lipm
_
9430028L06Rik
_
Snx25
_
Cpeb1
_
Syn2
_
Utrn
_
Zfp365
_
Cwc25
_
Scrt1
_
Tmeff1
_
Cthrc1
_
Gm10413
_
Rab43
_
Tdrp
_
Liph
_
Atpaf1
_
Sdc3
_
Ahcyl2
_
Olfm2
_
Arrdc3
_
Ablim2
_
Chn2
_
Nav1
_
Coro6
_
Adarb1
_
9630013P03Rik_
_
Gpr173
_
Adra2b
_
Pld5
_
Gng13
_
Laptm4b
_
Dner
_
Slc6a2
_
Mef2c
_
Atp2b1
_
Eya2
_
L3mbtl4
_
Oxct1
_
Crhbp
_
Tenm2
_
Dapk1
_
Satb1
_
Dcc
_
Nav2
_
Mef2d
_
Mxd4
_
Ppp3r2
_
Epha4
_
Syn1
_
Ppp1r14b
_
Pcdhb9
_
Pdxk
_
Pcdhac1
_
D930028M14Rik
_
TC1410973
_
Rasa2
_
Slc8a1
_
Gng12
_
Scn3b
_
Apbb2
_
Sirpa
_
Nefl
_
Psmc3
_
Sparc
_
Rnd2
_
Kcnj9
_
Necab1
_
Rgs17
_
Psmc6
_
Lats2
_
Fryl
_
Ppp2r2a
_
Agtr1b
_
Yipf1
_
Rap1gap2
_
Prkch
_
Chrm4
_
Miat
_
Cachd1
_
Kcnj9
_
Ackr2
_
Celsr3
_
Grm8
_
Kl
_
Ngef
_
Mras
_
Gpr151
_
Etv1
_
Itpr1
_
Gm13889
_
Cebpa
_
E330014M11Rik_
_
Ptger4
_
Hcn1
_
Ccr10
_
Arfgef2
_
Tyro3
_
Galr2
_
Mchr1
_
Gpr37l1
_
Tnnt1
_
B3galt5
_
Trim25
_
Ly6g6e
_
Gch1
_
Pantr1
_
Adra1d
_
Ephx2
_
Cacna1h
_
Necab3
_
Gtf2i
_
Cacna2d1
_
Cacna2d2
_
Cacnb2
_
Cacng4
_
Plch1
_
Clcn2
_
Kdelr3
_
Guk1
_
Kcnj5
_
Cck
_
Yeats2
_
Otx2
_
Prokr1
_
Glp1r
_
Chrna7
_
Ghsr
_
Psd
_
Grid1
_
Srm
_
Tpr
_
Stoml2
_
Prlhr
_
Egr2
_
Igfn1
_
Htr3a
_
Gpr3
_
Ache
_
Nrxn1
_
Rapgef5
_
Mgat4c
_
LOC436099
_
Scube1
_
Aldoc
_
Zdhhc7
_
Tns1
_
Rnf122
_
Prokr2
_
Aifm3
_
Atp6v1c2
_
Ccdc65
_
Kcnc2
_
Ppapdc2
_
Arhgap31
_
Sprn
_
Plch2
_
Adrbk2
_
Ltn1
_
Asap1
_
Entpd3
_
S100pbp
_
Gchfr
_
Gsto1
_
Idh1
_
Sv2b
_
Napb
_
Hsbp1
_
Rap1gds1
_
Kif5c
_
Inhbb
_
Nxf7
_
Adssl1
_
Hist1h2bg
_
Ndst4
_
Ntn1
_
Prss35
_
Nr2f6
_
Sox11
_
Extl3
_
Clstn2
_
Grm5
_
Gdap1l1
_
Rgs10
_
Ppp4r2
_
Esr1
_
Ptpn3
_
Wdr18
_
LOC434631
_
Slc6a4
_
Sema3c
_
Heatr5b
_
Ptgs1
_
Gsg1l
_
Daam2
_
Gpr176
_
Adcyap1
_
Rgs4
_
Wif1
_
Kcnh1
_
Rwdd2a
_
Atp11b
_
Krt9
_
Slc6a1
_
Prl2c2
_
Serpina3c
_
Ap3s1
_
Slc41a3
_
Penk
_
Auts2
_
Carhsp1
_
Lmna
_
Adcy4
_
Rbpms2
_
Tnfrsf25
_
Polk
_
Spns2
_
Stap2
_
Ssr2
_
Rtn4rl1
_
Kctd4
_
Bves
_
Dmp1
_
Manea
_
Alox12b
_
Nhlh1
_
Nicn1
_
Bnip3
_
Tmem196
_
Flt3l
_
Cnr2
_
Dpp6
_
Slc35g2
_
Pofut2
_
Sema5b
_
Hmbox1
_
Prkcd
_
Sgpp2
_
Rps6ka2
_
Ric8b
_
Clca3a2
_
Prrg2
_
Lzic
_
Pappa2
_
Rpl11
_
Ube2t
_
Per3
_
Map2k4
_
Pygb
_
Csrp3
_
Pcp4l1
_
Oprl1
_
LOC434368
_
Sipa1l1
_
Tmem145
_
Sept5
_
St6galnac3
_
Gprin1
_
C130021I20Rik
_
Cacna1i
_
Osbpl10
_
Supt5
_
Tiam1
_
Galr1
_
Lgr5
_
Hrh1
_
Kcnmb4
_
Lhcgr
_
Serpini1
_
Fezf1
_
Fam171a1
_
Pirt
_
Agrp
_
Anxa3
_
Fam180a
_
Btg2
_
Rims3
_
Cbln4
_
Cdh23
_
Chd5
_
Crim1
_
Crlf1
_
Rarres2
_
Eomes
_
Fst
_
Fstl5
_
Prkar1b
_
Cks2
_
Nefh
_
B230373P09Rik
_
Fam102b
_
Pwp2
_
Ttc39c
_
Leprot
_
Lgi3
_
Tuba4a
_
Rgs19
_
Alg1
_
Oprm1
_
Gria1
_
Slc6a3
_
Dnttip1
_
Cldn1
_
Clca3a1
_
Galns
_
Il4ra
_
Dhdds
_
Meis3
_
Ptp4a1
_
F11r
_
Opn1sw
_
Slc31a2
_
Spsb4
_
Rmdn1
_
Mri1
_
Ntpcr
_
Atp5sl
_
Ppp1r1a
_
Ppp3r1
_
Fam189a2
_
Mrpl12
_
Prdx3
_
Plekha2
_
Cpne2
_
Crlf3
_
Ctnnb1
_
Sbf1
_
Adamts16
_
Vat1l
_
Xlr3a
_
Sgk3
_
Tmem176a
_
Dusp6
_
Rnf219
_
Senp2
_
Csrp2bp
_
Asb8
_
Large
_
Camkv
_
Camk1g
_
Tmem41a
_
Plekha5
_
Nacc2
_
Gstm4
_
Sypl
_
Baalc
_
Bace1
_
Nab1
_
Pnpo
_
Gprin3
_
Myo16
_
Fam163b
_
Cd9
_
Saraf
_
Cdh7
_
Cpne8
_
Vcan
_
Dnah11
_
AF529169
_
Frmpd1
_
Sytl2
_
Prox1
_
Dpp6
_
Gpr126
_
Wdr5
_
Dlat
_
Acadvl
_
Hs6st3
_
Camkk2
_
Rnf115
_
Icam5
_
Il17ra
_
Fhl2
_
Itpkb
_
Tspan33
_
Zmynd11
_
Klhl1
_
Ccsap
_
Lin7a
_
Nptx1
_
Nrip3
_
Nxph4
_
Pak1
_
Pcdh11x
_
Pcdh7
_
Phactr2
_
Plxnd1
_
Pogk
_
Syt12
_
Gckr
_
Lrmp
_
Socs5
_
Cdc25b
_
Unc5b
_
Cds1
_
Aldh1a1
_
Ddr1
_
Diras1
_
Igsf11
_
Lhx9
_
Sh2b3
_
Cygb
_
Car2
_
Col4a6
_
Zfp57
_
Zic1
_
Sema3a
_
Nell2
_
Tbr1
_
Cd44
_
Dpysl5
_
Rgs2
_
Sccpdh
_
Stk24
_
Lypd6b
_
Kcnq4
_
4932418E24Rik
_
Hmgcs1
_
Cdkn1a
_
Tns3
_
Pdgfb
_
Rapgef4
_
Fam69b
_
Zfpm2
_
Filip1
_
Cckar
_
Mapk12
_
Fam65b
_
Trib2
_
Sidt1
_
Gpr55
_
Pacs2
_
Met
_
Tmem200a
_
Clip2
_
Cacna1g
_
Atp10b
_
Lypd6
_
Sgcg
_
Sgcz
_
Slc9a2
_
Prss39
_
Ano2
_
Vwa5b2
_
D11Ertd759e
_
Tnxb
_
Stard13
_
Ppap2b
_
H2-T23
_
Hspa4l
_
Sema4g
_
Aqp2
_
Tom1l2
_
Cul4a
_
Syt17
_
Trim23
_
Zfp61
_
Vwa3a
_
Gabrg3
_
Abcb6
_
Arsa
_
Gcg
_
Gabre
_
Grik3
_
Trim37
_
Dlk1
_
Kcnip3
_
Mturn
_
Neurod2
_
D830030K20Rik
_
Syn3
_
Sytl5
_
Ube4b
_
Lrrc74b
_
6430709H04Rik
_
Galntl6
_
Chd3os
_
Maoa
_
Sparcl1
_
Ednrb
_
Cntfr
_
Bcl6
_
Msmo1
_
Srp14
_
Col9a2
_
Epha10
_
Zeb2
_
6430573F11Rik
_
Map4
_
Sema6d
_
Gm5083
_
Tmem130
_
Cabp7
_
Emb
_
Oxct1
_
Asic2
_
Samd14
_
Ahsa2
_
C1ql2
_
Elfn1
_
Rxfp3
_
Hhip
_
Trerf1
_
Ube2e3
_
Mfsd6
_
Smdt1
_
Rprm
_
Chrne
_
Slc39a6
_
Grip2
_
Mlec
_
Tacc3
_
Spp1
_
Gabrp
_
Stoml1
_
P2rx3
_
Yjefn3
_
Epb4.1l1
_
Kcnf1
_
Cd200
_
Adck4
_
Coro1c
_
Chst12
_
Lrrc3b
_
Itm2a
_
Grb10
_
mCG1049722.1
_
Tmem108
_
Arnt2
_
Asb4
_
Dtnbp1
_
Kcnj14
_
Epdr1
_
Adamts19
_
Pid1
_
Lancl1
_
B630019K06Rik
_
Wfs1
_
C130096N06Rik
_
P2ry12
_
Chrna6
_
Cntn3
_
Gabrq
_
Magel2
_
Pln
_
Fndc5
_
Xlr3c
_
Sirt2
_
Alg5
_
Tmem206
_
Cysltr1
_
Slc27a1
_
Pir
_
Pole4
_
Cadm2
_
B3galt2
_
Vamp1
_
Sult1a1
_
Tmub2
_
Gria4
_
S100b
_
Tcf25
_
Ankrd34c
_
Pttg1
_
Ptcd2
_
Fam78b
_
C030034I22Rik
_
Fam19a4
_
Lmod2
_
B4galt1
_
C230009H10Rik
_
Tmem86b
_
Cdh9
_
Vwa5b2
_
Cort
_
Ddn
_
Gm261
_
Fam19a2
_
Hrh3
_
Cage1
_
Sox14
_
Nrep
_
Dnah9
_
Tnks
_
Mcu
_
D430041D05Rik
_
D830029A09Rik
_
Fam214a
_
Esyt3
_
Cdh4
_
Tcea1
_
Daf2
_
Fgf12
_
Hcrtr2
_
Dchs1
_
Mlx
_
Dusp4
_
Grp
_
Fam124a
_
Gpr101
_
Epha3
_
Epha6
_
Epha8
_
Erbb4
_
Ern2
_
Fbn2
_
Fgd3
_
Fibcd1
_
Slc6a11
_
Galnt13
_
Galnt14
_
Gfra2
_
Gnai1
_
Gnal
_
Gpc2
_
Kcnq5
_
Usp20
_
Ankrd34b
_
Cux2
_
Cks2
_
Fbxw7
_
Zdhhc2
_
Zfp385b
_
Vstm2b
_
Slc22a23
_
Ttll7
_
Gad2
_
Fam69c
_
Gfap
_
Fabp7
_
Rasd2
_
B4galnt3
_
Steap3
_
Col23a1
_
Col24a1
_
Clic5
_
Pcdh17
_
LOC433436
_
Vldlr
_
Pamr1
_
Stxbp6
_
Mas1
_
Osbpl6
_
Lppr5
_
Cemip
_
Raver2
_
Ube3a
_
Akap12
_
Pgf
_
Med23
_
Cables2
_
Tyr
_
Apbb2
_
Arhgap25
_
Atp10a
_
Slc18a2
_
App
_
Rfwd2
_
Itpk1
_
Lman2
_
Agpat4
_
Elmo3
_
Tex40
_
Defb1
_
BC031181
_
Chn1
_
Dnajc12
_
Adamtsl5
_
Utp23
_
Chl1
_
Ccr1
_
Grm1
_
Lrrtm1
_
Lhx1
_
Zbtb24
_
Tmem215
_
Fam184b
_
Syt13
_
Iqsec3
_
Tmtc1
_
Nceh1
_
Doc2g
_
Arntl
_
Dab1
_
Cd164
_
Bnc2
_
Dnaja4
_
Adamtsl2
_
Abcb10
_
Cab39l
_
Tmem74
_
Ogn
_
Il31ra
_
Gpr123
_
Crtac1
_
Alox8
_
Traip
_
Ano6
_
Vdr
_
Mme
_
Rtn4
_
Ebpl
_
Osbpl9
_
Psg16
_
Ndufv2
_
Sh3bgrl2
_
Nav1
_
Grik1
_
Asic4
_
Dab1
_
Apbb3
_
Lrp8
_
Hbb-b2
_
C1qa
_
Gemin4
_
Nnat
_
Tm2d3
_
Gjc1
_
Ano6
_
Slc6a20b
_
Tmc6
_
Atp5h
_
Tdrd3
_
Lingo2
_
Csdc2
_
Deptor
_
Gldc
_
Pip5k1b
_
Tmem35
_
Ctss
_
Postn
_
Aspa
_
Rmnd1
_
Ampd3
_
Whrn
_
Kcnn2
_
Kcns3
_
Ptcd1
_
Sstr2
_
Tmem255b
_
Alk
_
Grid2ip
_
Bcas1
_
Thsd7a
_
Scd1
_
Grn
_
Cnp
_
Abhd3
_
Kctd1
_
Stk32c
_
Vgf
_
Sdcbp
_
Klf10
_
Ccnd2
_
Tac1
_
Grk5
_
Hmgcr
_
Med8
_
Gng7
_
Zfp280c
_
Klhl36
_
Kcne3
_
Gm1399
_
Rora
_
Rfx1
_
Pptc7
_
Bok
_
Pcsk1
_
Slc35e2
_
Acacb
_
Pde10a
_
Apc
_
Nlk
_
Tpbg
_
Pbx3
_
Fdps
_
Vav3
_
Lzts3
_
Ltbp4
_
Sphkap
_
Phactr4
_
Ppm1l
_
Lancl3
_
Col9a1
_
Brwd1
_
Atg12
_
Sash1
_
Usp13
_
Scube2
_
Foxp2
_
Atp2a2
_
Slc24a2
_
Unc5d
_
Rfk
_
Vdac1
_
Scn3b
_
Oprd1
_
Loxl1
_
Atp6v0c
_
Atp8a1
_
Utp14b
_
Rnf10
_
Wnt8a
_
Ptprz1
_
Glipr1
_
Nkd1
_
Gpd1
_
Elmod1
_
Wrb
_
Dclk3
_
Fam167a
_
Abat
_
Cebpd
_
Mb21d2
_
B2m
_
Fam114a2
_
Col25a1
_
0610010F05Rik
_
Fbxo18
_
Anapc16
_
Rpf2
_
Cdc27
_
Rpl21
_
LOC66376
_
Paf1
_
Rad51
_
Stac2
_
Arf3
_
Ms4a6b
_
Vip
_
Slit2
_
Scrg1
_
Ddx19b
_
Pomc
_
Ppp1r9a
_
Klhl4
_
Sgtb
_
Frmd6
_
Tnmd
_
Cd109
_
Casc1
_
Mpp6
_
Clmp
_
Col11a1
_
Taf1d
_
Tal1
_
Creb3l1
_
Bhlhe41
_
Decr2
_
Rnf220
_
Rab3c
_
Rai14
_
Gkn3
_
Stard8
_
Tle4
_
Tmsb10
_
Tshz3
_
Npr3
_
Pdcd4
_
Rnf19a
_
Acaa2
_
Trim16
_
Flrt2
_
Ptrf
_
LOC235953
_
Robo1
_
Frmpd4
_
LOC380720
_
Slc1a2
_
LOC432928
_
Adamts2
_
Syndig1
_
Gata3
_
4833414E09Rik
_
Tcn2
_
Sigmar1
_
Phf6
_
Uqcrfs1
_
Fhod3
_
Gabra4
_
Inpp4b
_
Garnl3
_
Greb1
_
Gng12
_
Atrx
_
Arhgap27
_
Zfp932
_
Hpd
_
Stxbp2
_
Fam65a
_
Trhde
_
Ubl4
_
Tmem246
_
Ptk2
_
Mtfp1
_
Hspbp1
_
Kcnb2
_
Lgi1
_
Arhgdia
_
Loxl2
_
Lrp4
_
Clip4
_
LOC235580
_
Atpif1
_
Nucb2
_
Mmp16
_
Pomp
_
Nrbp2
_
Rps15
_
Neto1
_
Bcl2l12
_
Ralyl
_
Cntnap3
_
Dpf3
_
Spink8
_
Efcab3
_
Hs6st2
_
Itgb1
_
Nr2f2
_
Asxl3
_
Car9
_
Dicer1
_
Dnah12
_
Gm22
_
Alpk2
_
Sec61a2
_
Nap1l5
_
Ascl5
_
Bad
_
Mrap2
_
LOC270764
_
Ufm1
_
Myo1b
_
Ndnf
_
Plcz1
_
Tshz1
_
Lipa
_
LOC381355
_
LOC545810
_
Lrrn1
_
Tshz2
_
Oxr1
_
Eps15
_
Rtn4r
_
Prdx6b
_
Cdkn1b
_
Ntng1
_
LOC433088
_
Ascl2
_
Vill
_
Sdc2
_
Tmed3
_
Slc7a6
_
Prpsap2
_
C1qtnf5
_
Fndc4
_
Uck1
_
Bap1
_
Serpinb8
_
Rtl1
_
Slc8a3
_
Id4
_
Fgd5
_
Peg10
_
Kctd6
_
Trnp1
_
Btg1
_
Atp6v0b
_
Dexi
_
Pou6f1
_
Rps15a
_
2310003H01Rik
_
Moxd1
_
Slco3a1
_
Aqp3
_
Mum1l1
_
Zfand3
_
Nell1
_
Kcnj4
_
Ptk2b
_
Tpd52l1
_
Rgs5
_
Vat1
_
Tspan18
_
Mapk11
_
Cryab
_
Smoc2
_
Gdap10
_
Ndufaf2
_
Mrc1
_
Megf11
_
Pdp1
_
Teddm3
_
Sv2c
_
Epn3
_
Pkd2l1
_
Pgf
_
Mup5
_
Ngp
_
Osbpl5
_
Pam
_
Pitpnm2
_
Pkp2
_
Grb14
_
Rcn2
_
Spata13
_
Tspan12
_
R3hdm4
_
Lct
_
Svip
_
Arhgef26
_
Peak1
_
Chst11
_
Clmn
_
Creg1
_
D030063F01Rik
_
Rgs7bp
_
D8Ertd82e
_
Itgb8
_
Zfp804b
_
Dusp5
_
Gm626
_
Acsl5
_
LOC433258
_
Lrrtm3
_
Mgat5
_
Nova1
_
Pde5a
_
Pgbd5
_
Rab37
_
Slc22a3
_
Kcng1
_
Ppapdc1a
_
Actb
_
Chrm2
_
Kank4
_
Tbck
_
Arap2
_
Arpp21
_
Npnt
_
Npr1
_
Ntrk1
_
Pigq
_
Pald1
_
Pclo
_
Pde7b
_
Cdk14
_
Phospho1
_
Inpp5j
_
Plekhg1
_
Prkg2
_
Tox
_
Zim1
_
Sec14l1
_
Pvr
_
Arl4c
_
Slc2a6
_
Steap2
_
Itgbl1
_
Rnaset2b
_
Nkx2-1
_
Tmem109
_
Ttll4
_
Gm12429
_
Txnrd3
_
Smarca2
_
Unc5c
_
Vangl1
_
Zyx
_
Tmem163
_
Zfp618
_
2900026A02Rik
_
Xlr4c
_
Zfand2b
_
Gnal
_
LOC433740
_
Rasal2
_
Trav3-3
_
Ankrd50
_
Kctd8
_
Gpr88
_
Ttc6
_
Gm196
_
Drc1
_
Rps6ka3
_
Serpina9
_
Ehbp1l1
_
Slc9a9
_
Adam33
_
Tas2r144
_
Ust
_
Enpp2
_
Zfp462
_
Ctsz
_
Pcdh20
_
Midn
_
Lrp1b
_
Sntb1
_
Slc10a4
_
Evi5
_
LOC381557
_
Cyp46a1
_
Chst15
_
Brinp2
_
Psrc1
_
9530068E07Rik
_
Emp1
_
Copg2
_
Etv5
_
Rgs12
_
Atg3
_
Rnf144b
_
Slc17a8
_
Plxna2
_
Mast4
_
Tob2
_
Eif1ax
_
E2f4
_
Gm2a
_
Sfn
_
Psmb2
_
Nde1
_
Hook3
_
Tdrd3
_
Taldo1
_
Sfrp1
_
Dynlt3
_
Cyp39a1
_
Lysmd2
_
Pip5k1b
_
Ttc25
_
LOC381765
_
St3gal3
_
Paqr8
_
Gramd3
_
Tbl3
_
Rps21
_
Mgst3
_
Kcnj14
_
Gatm
_
Grm3
_
Lhfpl3
_
Kbtbd3
_
Mgat2
_
Numa1
_
Klk6
_
Atp1a4
_
Trhr
_
Tm2d3
_
Slc12a3
_
Rab12
_
Wwc1
_
Paqr4
_
Rassf4
_
Selplg
_
Cntn6
_
Terf2ip
_
Tbc1d14
_
Scube3
_
Sel1l3
_
Tspan15
_
Cops4
_
Adcyap1r1
_
Cacna1e
_
Calcrl
_
S100a10
_
Chrnd
_
Igfbp4
_
Gabra2
_
Creg2
_
Cyp4v3
_
Cyp26b1
_
Dnajb12
_
Lhx2
_
Gdnf
_
Cdh8
_
Efr3a
_
Mtap
_
Pcdh8
_
Grin2b
_
Ttyh3
_
Stk38l
_
Tmx4
_
Fasn
_
Cyb561
_
Crip2
_
Dpp4
_
Bsdc1
_
Fnip2
_
Snapc2
_
Ren2
_
En2
_
Nfic
_
Sf3b2
_
Sox8
_
Tcea2
_
Ubtf
_
Mdk
_
Slc25a39
_
Kansl3
_
Cep72
_
Plk5
_
Pcsk2
_
Cpne3
_
Txnrd1
_
Vegfa
_
E030007A22Rik
_
Grip1
_
Armc2
_
LOC214238
_
5730409E04Rik
_
Gm973
_
LOC380889
_
Lonrf2
_
LOC381742
_
Decr1
_
LOC545352
_
Fndc3b
_
Acyp1
_
BC005561
_
Klhl9
_
Gpd1l
_
Hist1h1a
_
Hs3st2
_
Lamc2
_
Gm1335
_
Glul
_
Dbi
_
Gprc5b
_
Ldhd
_
Nbr1
_
Ndrg2
_
Otud6b
_
Pak3
_
Ctxn1
_
Rtn3
_
Abcd2
_
Cnbd2
_
Copg1
_
Nr1d1
_
Ctsb
_
St8sia1
_
Sap30l
_
Gpr116
_
Chrm1
_
Fzd9
_
Grin3a
_
Pappa
_
Scn5a
_
Trpm6
_
Prpf38b
_
LOC620538
_
Ostf1
_
Prkcsh
_
Smpd1
_
Ptpn2
_
Vldlr
_
Stat5a
_
Clgn
_
Nfxl1
_
Tbl3
_
Slc39a5
_
Flrt3
_
Sema3f
_
P2ry14
_
Pcp2
_
Dtna
_
Reep5
_
Matn2
_
Galnt9
_
Ascl1
_
Zcchc12
_
Gpr12
_
Arl4d
_
Papola
_
Bmp5
_
2610028E06Rik
_
Capn2
_
Col12a1
_
Cpne7
_
Map2k6
_
Nefm
_
Ppp3ca
_
Ptprv
_
Rgs20
_
Rnasel
_
Syt9
_
Tnnc1
_
Spata2l
_
Arl4d
_
Slc39a3
_
Dlg2
_
Magix
_
Pcnt
_
Dkk4
_
Kcna2
_
Gpr115
_
Msrb2
_
Reps2
_
Fam107b
_
Zfp521
_
Elmo2
_
Mmel1
_
Eml3
_
Itgb1bp1
_
Rasgrp4
_
Gtf2f1
_
Gpr18
_
Resp18
_
Rnaseh2c
_
Chchd3
_
Cox7b
_
Homer2
_
Pknox2
_
1110008P14Rik
_
Pdia3
_
Trib1
_
Aco1
_
Matk
_
Gria2
_
Glra2
_
Tmem159
_
Doc2a
_
Deptor
_
Ddr2
_
Nr1h3
_
Pir
_
Ebf4
_
Gipc1
_
LOC544975
_
Nme1
_
Tspan2
_
F3
_
Uts2b
_
F630102L10Rik_
_
C130018E23Rik_
_
Sptb
_
Syt10
_
Uqcr11
_
Sssca1
_
A330043P19Rik_
_
Wnt8b
_
Stat1
_
Pitpnc1
_
A730096I18Rik_
_
Tspyl2
_
Plcb1
_
Mpv17l2
_
Abcb6
_
Synpo2
_
Dos
_
Myo10
_
Ndufb8
_
Nrg1
_
Pacrg
_
Pcdhb13
_
Snx33
_
Elovl1
_
C230071H18Rik
_
Pcdhb19
_
Pcdhb2
_
Plk2
_
Prkar2a
_
Psmd11
_
Ptpn4
_
P2ry2
_
Wnt9b
_
Gabpb1
_
Enpp6
_
Ptpn5
_
Stx1a
_
Ica1
_
F13a1
_
Ptprk
_
Efnb2
_
Usp21
_
St3gal1
_
Kcns2
_
Fa2h
_
Dvl2
_
Slc38a1
_
Galnt6
_
F2rl1
_
Mpped1
_
Timp2
_
Tmod1
_
Haghl
_
Mrps28
_
Fam3c
_
Hspb6
_
9430020K01Rik
_
Ntng2
_
Itga11
_
Etv1
_
Slc35a4
_
Ncald
_
Itga5
_
Gabarapl1
_
Dpysl3
_
Fam20c
_
Kcnj12
_
Pak7
_
Podn
_
Erp29
_
Thra
_
Dlx6os1
_
Mfn2
_
Map3k19
_
Nrsn2
_
Stub1
_
Fos
_
Chrm3
_
Mxra7
_
Ticam1
_
Ncs1
_
Nmbr
_
1190002N15Rik
_
Nsun7
_
Foxb1
_
Spsb1
_
Hecw1
_
Siglech
_
Vwc2
_
Gucy1a3
_
Ddx26b
_
LOC434300
_
Anxa6
_
B4gat1
_
Pgrmc2
_
Ppp2r2c
_
Cdhr1
_
Rock2
_
Dgkb
_
Nrxn1
_
Tagln3
_
Sorcs3
_
Rasl11a
_
Prkdc
_
Psmd13
_
Cpne5
_
Kiss1
_
Pcp4
_
D10Bwg1379e
_
Adcy8
_
Pfdn4
_
Cadps
_
Cntnap2
_
Ly6h
_
Tbc1d9
_
Ltk
_
Egr4
_
Pnoc
_
BC005624
_
Alg2
_
Tnfaip8
_
Ptk7
_
Ubap1
_
Cspg5
_
Itm2c
_
Ak1
_
Drd3
_
Ptprr
_
Trem2
_
Inpp1
_
Ptprs
_
Purg
_
Zfp189
_
Rarb
_
St18
_
Arf1
_
Rfc5
_
Sorcs1
_
Uaca
_
Oxtr
_
Npvf
_
Tspyl4
_
Rpl15
_
Wdr31
_
Psme2
_
Amotl1
_
Camk4
_
Gprc5a
_
Pfkm
_
Mlx
_
Rin1
_
Slc29a4
_
Gng2
_
Selk
_
Ptdss1
_
R74862
_
Pmm1
_
Hbb-b1
_
Ugt8a
_
Slc50a1
_
Prkcsh
_
Sacm1l
_
Pcdha10
_
Phyh
_
Nqo1
_
Sc5d
_
Cbfa2t3
_
Pde11a
_
Rnh1
_
Tesc
_
Il17rd
_
Hiatl1
_
Galnt10
_
Soga1
_
Fbxo21
_
B230362M20Rik
_
BC053994
_
Hbb
_
Hexb
_
Hpse
_
Gm1088
_
Trps1
_
Kpnb1
_
Stard7
_
C1qc
_
Egln1
_
Tmem150c
_
Mapk3
_
Plxnd1
_
Tulp4
_
3100002H09Rik
_
Kcnab3
_
Gm6753
_
Pim2
_
Gem
_
Slc7a3
_
Gyg
_
Cd6
_
Chd6
_
Arl16
_
Eef1a1
_
Ube2k
_
Ncoa7
_
Pitx1
_
Fbxo44
_
Krt73
_
Lrrc55
_
Uqcrh
_
Nfs1
_
Slc8a1
_
Kcnn4
_
Kdelr1
_
Rarg
_
Scd3
_
Sf3a2
_
Glrx
_
Ier5
_
Slc12a2
_
Scn1a
_
Zkscan16
_
Lats2
_
Nos1ap
_
Aqp4
_
Slc2a1
_
Slitrk1
_
Slitrk4
_
Cstb
_
Tecta
_
Rufy3
_
Nt5dc2
_
Ssx2ip
_
Exoc6
_
Ssbp4
_
Ppp1r17
_
Prkcg
_
Tm4sf1
_
Hap1
_
Manba
_
Stam
_
Gpr171
_
Hebp1
_
St6galnac6
_
Prkg1
_
Cldn11
_
Gm4887
_
Usmg5
_
Dact2
_
Gpr50
_
Zdbf2
_
LOC329302
_
Sgsm1
_
Gpr4
_
Atp11c
_
Clic1
_
Slc18a3
_
Tro
_
Gm17746
_
Strn
_
Grb7
_
LOC433402
_
Psg16
_
Eef2
_
Msi2
_
Scml4
_
Pcbp4
_
Egflam
_
Chml
_
Rpgr
_
Gda
_
Il11ra2
_
Wnt10b
_
Slc6a3
_
Btrc
_
Gsn
_
Sptssa
_
Nkain4
_
Eno2
_
Car12
_
```

In [10]:

```
view_mouse()
```

```
73994713 Inpp5a
3 Snap47
5 Nvl
7 Gtf3c2
72081416 Acadl
75081206 Baiap3
77278967 S100a6
273 Clock
17 Mageh1
72081426 Sncg
112649906 Hnrnph2
655497 Nnat
26 Cdca7
29 Prkag2
74957909 Uggt1
31 Dopey2
33 Anln
35 Hepacam
37 Setd5
39 Cnih3
44 Thap1
74641209 TC1541413
79556616 Htr1a
51 9130024F11Rik
53 Mansc1
55 Pmch
57 Prune2
1462 Il1rap
60 Acta1
10 Pcdh9
62 Actc1
64 Actr10
75079745 Ctla4
72007746 Rell1
67 Actr2
72007749 Fut8
72007750 Il34
71 9130024F11Rik
73 Adcy2
72081482 Scarb2
75 Adcy3
77 Adcy4
79 Adcy5
81 Adcy6
83 Adcy9
72081492 Rps19
85 Agpat3
87 Agt
89 Mllt11
91 Akap8
94 Anp32a
75079775 Mt3
75079776 Capsl
75079777 Etnppl
100 Gata2
70814345 Cdc42ep1
74047909 Rab6a
106 App
75079787 Ifi205
72081516 Spag5
79556626 Runx2
110 Arf3
113 Arhgap5
77867710 Hgf
118 Arx
75079799 Recql5
75079800 Thbd
75079801 Cit
71015807 Tmeff2
124 Atp5j
72119658 Htr4
126 Atp6v0a1
129 Atp6v1b2
1387 Nes
133 Tmem50b
71016583 Rbp4
71016584 Stat5b
71016585 Rbm19
72081547 Prrt3
72081548 Cyp26b1
655501 Atp6v1e1
72081550 Fign
143 Bace2
72081553 Gnao1
72081554 Slc32a1
71016595 Stmn1
71016596 Fstl1
149 Usp48
72081558 Asb13
72081560 Kcng4
72081561 Gpr98
154 Bcan
1369 Lhx5
77414563 Ptcd2
77414565 Cyhr1
166 Bsg
168 Btg3
77414569 Gpalpp1
71016618 Btbd3
172 Nrcam
655502 Uqcrc2
179 Calm1
71016630 Setd7
71016631 Trh
184 Calm3
77414585 Mmgt1
77869087 Ldb3
189 Camk2g
1397 Pex14
77332673 Iqsec1
71064267 Synj2
196 Ctnnb1
198 Cbx5
71064217 Gfra1
1399 Pfkfb2
112648396 Oxt
71016653 Slc6a7
77332686 Ece2
74881263 Atp1a3
74511736 Car7
211 Ccnl2
71016663 Hdc
71016664 Ptgs2
71016665 Bcat1
71016666 Col5a2
219 Cd1d1
72081445 St8sia5
74047712 Gpc5
71670480 Insrr
74047714 Ankfn1
1403 Plxna3
74047716 Pdzd8
74047717 Sorcs2
71670481 Kcnc3
232 Cdh11
77332713 Parvb
75749741 Clcn5
77332715 Mtss1l
236 Cdh2
71670482 Lamb1
238 Cdh6
1405 Prnp
77414641 Bpgm
75774675 Rpn1
244 Cdk5
247 Cdk5r1
73788116 Stac
250 Cds2
74581375 Rtn4rl2
77414652 Itm2c
72007933 Nsdhl
72007934 Lhfpl2
72080085 Slc16a3
72007936 Vwa1
72007937 Kazn
74047746 Ttn
74047747 Gm12680
74047748 Astn2
261 Chrm4
71016711 Slc52a2
266 Chst2
75774679 Nup93
77332748 Svil
74047758 C4b
71016721 Dhcr24
275 Clu
278 Cnih1
72129241 Klk8
74047768 Serpine2
74047769 Dach1
74047771 Gnb4
74047772 Lypla1
74047773 Nptx2
74047774 Spint2
74047775 Fzd6
74047776 Itpr3
74047777 Epb4.1l1
290 Col6a1
72129243 Mgll
292 Crh
297 Crhr1
74581383 Ackr1
74047788 Asns
74047789 Gdpd2
302 Cry2
72129245 Gstk1
304 Cryzl1
74581384 Ngf
308 Cxcl12
72129246 Tsfm
79591675 Cnr1
73732148 Twf2
314 Tnrc6a
73992927 Camk1d
316 Srgap3
318 D16Ertd472e
320 Tomm70a
73992928 Cd59a
322 Ubxn11
79556662 Calb2
326 Dbh
72129249 Ccdc109b
329 Dcn
71064290 Il16
334 Ddc
337 Ddx3x
74988259 Zmiz1
71358554 Pcdh18
74047829 Stk26
343 Dgkg
346 Dkk3
74511759 Gpr133
348 Dlx1
79556666 Cdh2
350 Dlx3
74047839 Tspan6
352 Drd1
74047841 Pla2g7
74047842 Vps39
74047843 Tfap2c
357 Drd2
73769319 Clstn1
73769320 Tmem204
73769321 Khdrbs3
73769322 Cpne6
73769323 Lhfp
73769324 Timp3
73769325 Btbd11
73769326 Pnkd
74047855 Slc25a10
74047856 Kcnb1
369 Acsbg1
74581422 Apol8
73992937 Csf2rb2
378 Erdr1
77414600 Tmem14a
381 Efna3
383 Efna5
75041515 Magi3
74047876 Slc10a3
74047877 Lztr1
390 Enc1
393 Entpd1
74581399 Fam135b
75079746 Vegfb
74047888 Tango2
402 Epha7
72007747 Nxn
404 Eps8l3
408 Ewsr1
74047898 Cd34
74047899 Hspa12a
74047901 Fam213b
414 Cntn1
69 Adamts1
74047904 Nedd4l
70634224 Fam134b
74047906 Hsdl2
74047907 Sqrdl
112648612 Ap2b1
74957922 Zfp114
74047910 Setd3
74047911 Ifit3
74047912 P3h3
74724764 Npas2
74047914 Csrnp2
427 Fgf13
74047916 Glo1
74047917 Azin1
74047918 Glul
74047919 Tex261
74047920 Pacsin2
74047921 Podxl2
74047922 Ogfod1
74047923 Abhd6
74047924 Stx3
70301086 Sybu
74047926 Pacsin1
72008119 Ier3
440 Fgfr1
72008121 Fmo1
72008122 Gas6
72008123 Cgnl1
445 Fgfr2
448 Fgfr3
73925714 Zmym2
275873 Tpm3
70562113 Kcnh8
75041527 Ociad2
275874 Copz2
74511924 Sorl1
466 Fzd3
74819342 Dmrt3
470 Fzd8
75080151 Sema6c
472 Gabrb2
77868786 Gbx2
474 Gabrg2
476 Slc6a1
479 Gad1
77414882 Alkbh6
483 Gas5
77414884 Synj2bp
485 Gata2
491 Gdi2
73519850 Shoc2
77414895 Pias4
496 Ghrh
77414897 Vcam1
498 Glrb
500 Gls
74988286 Pdzd2
503 Glud1
506 Gnao1
509 Gnas
77414911 Edc3
512 Gnb2
77414913 Tmem126a
2521 Rasd1
77414915 Plcd3
79556694 Grin1
75080199 Spag16
77414921 Asb8
522 Grb10
77414923 Zfand3
77414925 Ppp2r3d
73636101 Rspo1
75080209 Rnf32
73732146 Ppp1r1b
74511790 Mc3r
537 Grm1
74511791 Col15a1
70615583 Ctbp2
74881547 Arhgef6
1456 Gnb5
75080228 Ceacam14
549 Hap1
552 Hap1
554 Hars
70615595 Fyn
556 Hes1
70615597 Pgm2l1
558 Hgf
75080239 Rrad
560 Hist1h2bc
565 Hlf
75080249 Aif1
75080250 Ybx2
571 Hnrnpdl
573 Ntm
577 Prmt2
579 Hspb7
74581430 Fam120c
584 Htr1b
72129292 Ngef
588 Ifnar1
75080269 9530085C10Rik*
1695 Ctf1
592 Igsf5
1464 Tsc22d4
594 Impact
596 Isl1
598 Itga3
75080279 Pde4d
600 Itih3
72339556 Tac2
602 Jam2
606 Jund
72129296 Hist2h2aa1
614 Kifc2
616 Kitl
618 Klc2
74881537 Arhgef5
620 L1cam
74511805 Flot2
72008304 Plcb4
625 Lsamp
72008306 Them6
357093 Hmg20a
2203 Exosc1
74658141 Gipc2
633 Lhx1
635 Lhx6
637 Lig1
77866882 Ptn
112648831 Mdfi
640 Limk1
1472 Ano1
73818754 Slc17a6
73818755 B3gat1
73818756 Ogfrl1
73818757 Gmppa
73818758 A830018L16Rik
73818759 Eya1
649 Lpl
72108823 Cacng8
70927810 Slc24a3
655 Lynx1
75041560 Cst6
74581443 Panx2
73925721 Adarb2
75041561 Rassf6
665 Mcm3ap
667 Mef2c
670 Marcksl1
70927813 Cyp7b1
673 Mrpl39
675 ND3
1478 Cpt1a
680 Mup2
74658130 Cs
682 Ncam2
684 Ncdn
686 Ncor1
688 Ndn
690 Ndrg3
692 Negr1
698 Neurod6
1482 Dlx2
705 Ngfr
713 Rrp1
715 Npdc1
717 Npy
720 Nr2f1
80516967 Sema5a
727 Nr3c1
730 Nr3c2
73512185 Hcn2
732 Nr4a2
734 Nr5a1
72080165 Appl2
736 Nrgn
739 Nrip1
741 Nrtn
73817429 Cadps2
743 Ybx1
746 Ntrk2
748 Oaz2
71016573 Lifr
752 Tenm3
754 Tenm4
756 Sebox
71924431 Adamts17
758 Ogt
760 Olfm1
70927828 0610007P14Rik
764 Pax6
766 Pax7
768 Pcbp3
770 Pcdhga12
772 Pcp4
70927830 Ctu1
777 Pcsk1n
75145997 Isyna1
75145998 Ggt7
75145999 Lims2
75146000 Susd2
785 Pde9a
75146002 Psmd5
75146003 Zswim6
75146004 Pip4k2a
75146005 Mpnd
75146006 Rpp25
74819326 Fam105a
79591691 Ngfr
793 Per2
795 Pfkl
77371787 S100a16
797 Pgrmc1
799 Pip4k2c
77332746 Sdk2
73615572 LOC434236
805 Pkia
357099 B4galt3
808 Pld3
74819036 A830073O21Rik
72008490 Dlx1
544903 Hoxa5
72008493 Wnt9a
72008494 Pcsk1
74272904 Sp8
820 Plxnc1
74657929 Nhlh2
825 Pmp22
827 Pnck
74657930 Trim36
830 Ppap2a
832 Ppp1r7
72077451 Wwox
836 Prdx1
840 Prkca
356492 Hpd
75079788 Cystm1
74988343 Thnsl1
844 Npepps
846 Psap
72077453 Gabra5
851 Ptgds
142 Islr2
112649047 Lrrc3
858 Ptpru
864 Ptprm
72077456 Crispld1
866 Ptprt
72080187 Chst8
868 Pvalb
870 Dnajc16
145 Acot7
74357564 Pon3
876 Os9
74581420 Scai
878 Rab1
74750077 Atp11c
881 Rad23b
75890835 P2rx1
888 Rbm4
72081556 Mesdc2
890 Reln
74881855 Plekhg5
895 Rfx1
74988352 Veph1
898 Rnf149
900 Rora
906 Rpn2
909 Rtn2
74641238 Vti1a
917 Rxrg
923 S100b
925 Samsn1
927 Satb1
930 Sbno1
932 Atxn1
934 Scg2
937 Sema3a
75080619 Exph5
75080620 Fam120a
942 Sema4a
945 Sema4d
72080084 Mtch2
950 Sema5a
75080632 Purb
75080633 Capzb
955 Sema7a
74357578 Igsf21
1752 Irx2
75774678 Susd4
961 Sept7
75080643 Dscaml1
75080644 Synpo
75080645 D630014O11Rik
75080646 C530008M17Rik
967 Slc18a2
70927304 Tgfb2
75081207 Fam60a
972 Slc25a3
75080656 Tmem29
1528 Rara
70616018 Cttn
70616019 Pcsk5
980 Slit2
982 Arid1b
984 Snap91
986 Snca
72080207 Slc9a1
73769125 Clic4
994 Sod1
996 Son
1001 Sst
1006 Stmn4
72129291 Hpca
1009 Stxbp1
75774107 Slc35b4
1014 Syt1
71016617 Blcap
1020 Syt13
170 Rbbp6
1023 Syt2
73636038 Rasl10a
1026 Syt3
1029 Syt5
1032 Syt6
1035 Syt7
77371795 Matn4
227535 Spi1
1041 Tbr1
1044 Tcf4
1049 Tff1
1051 Tsc22d1
1056 Th
1059 Tiam1
1061 Tle1
1063 Trappc10
1065 Tnni3
1543 Tm7sf3
74957868 Tmie
1069 Slc36a2
75080750 Gpx3
1071 Trim28
1074 Trim9
67855413 Gosr2
67855414 Trp53
1079 Ttc3
1081 Tubb4a
67855419 E2f4
1084 Txnip
74819250 Scd2
1086 Ubash3a
74957887 Tmprss5
1088 Uchl1
70927425 Slc28a3
1091 Ucn3
1092 Vamp2
182 Calm2
74881889 Bche
74957897 Top1
1098 Slc32a1
71836747 Tbc1d8
71836748 Dnajb1
1549 Vdac3
1104 Vipr2
71016632 Rspo2
1106 Vsnl1
73925715 Pot1b
74957908 Ubr1
73925717 Fhdc1
73925718 Orai2
74957911 Usp22
73925720 Adamtsl3
1113 Wnt7b
73925722 Agrn
73925723 Atp8a2
73925724 Lppr3
73925725 Prex1
1119 Xbp1
74357551 Scrn1
1122 Yars
77414587 Tinf2
1124 Ywhag
1127 Ywhah
1130 Ywhaq
75774111 Rasal1
1133 Ywhaz
77414589 Tmem64
71836787 Pdzrn3
1140 Zfand5
1143 Zbtb20
1144 Ikzf1
191 Car8
1148 Crls1
71836797 Spon1
71836799 Rassf3
71836801 Zfp536
71836803 Cib2
71836804 Pck2
71836805 Tmx2
71836806 Id2
71836807 Sbk1
71836808 Sostdc1
71836809 Pip5k1c
71836810 Acsl6
75774112 Tmem168
1164 C130060K24Rik
1168 Cbfb
1173 Chrna4
1175 Cnp
1177 Col6a2
77866900 Lef1
1179 Crabp1
1181 Hspb8
71836830 Vwa5a
1183 Ctgf
71836832 Lppr2
1185 Ctps
71836834 Trp53bp1
1187 Ctsl
71836836 Ppp2ca
1189 Cyp4f15
71836838 Bid
1191 Cyr61
71836840 Acat1
71836841 Mcam
71836842 Plekhb1
74425515 Soat2
74425516 Sh3d19
71836845 Adamts4
71836846 Arhgap12
1199 E4f1
71836848 BC034076
71836849 Ier2
74425522 Sln
1203 Fjx1
74425524 Tnfrsf19
74425525 Aox1
74425526 Gfpt2
1207 Ghitm
1209 Gng3
1211 Gphn
1217 Icosl
75041420 Ctsw
74425539 Cda
74425540 Tnip2
74425541 Gtdc1
74425542 Akr1c18
71836871 Echdc2
71836872 Chst1
74425545 Zdhhc9
71836874 Nmb
74425547 Jup
74425548 Sun1
71836877 Upp1
71836878 Ddit4l
1231 Meis2
74425552 Rasa4
74425553 Amot
71836882 Nudt4
71836883 Tm6sf1
71836884 Chd7
71836885 Tnfrsf21
71836886 Dpy19l3
1239 Nfia
71836888 Slc1a1
71836889 Ap2s1
71836890 Bckdhb
71836891 Asb11
71836892 Maged2
1245 Nr1d2
74425566 Ifltd1
1247 Map3k7cl
74425568 Glcci1
74425569 Afap1
79589524 Gabrr2
74425571 Stat2
74425572 Sorbs1
75774115 Npc2
71836902 Gjd2
71836903 Ehd3
1257 Pde1b
1259 Pde4d
1261 Pfn2
1263 Plxnb2
1265 Plxnb3
1268 Prlr
1270 Cytip
1274 Rasgrf1
1276 Rnf11
1278 Runx2
1282 Slc19a1
1579 Atp5a1
1284 Sox5
1287 Synj1
70231304 Sesn1
70231305 Ecel1
70231306 Cbln2
70231307 Cdh24
70231308 Slc20a2
1295 Tacr1
1297 Tacr3
1299 Tmeff1
1301 Top2a
1303 Trhr
1305 Trpc4
77925007 Aldh5a1
1309 Vim
1311 Morc3
1313 9130024F11Rik
1315 Shroom1
75080996 Zfp260
75080997 Arhgap26
75080998 Robo2
75080999 Calcr
75081000 Grin2a
1321 4932438H23Rik
75081002 Trpm1
74271623 Ppp1r9b
75081004 Alg14
1325 Vps18
75081006 Ccdc85a
1327 Aire
1329 Atm
73512499 Ace
1335 Cbs
1337 Cdh3
1341 Copb2
74511925 Tph2
1345 Dpysl4
1349 Elf2
1351 Esrra
1353 Etv6
1355 Aktip
1357 Gfap
1359 Gjb2
1409 Tmem2
1361 Gmps
1363 Grp
112650769 Pla2g16
1365 Itsn1
1367 Krtap12-1
71717570 Gm1125
71016676 Camk2d
1371 Lrig1
1373 Lmo1
74819653 Cyfip1
1375 Samd3
1379 Msto1
1381 Mki67
1383 Mmp15
1385 Mx1
77332711 Mpzl1
1391 Syncrip
1393 Ntf5
74581365 Jak1
74581366 Tsc22d3
74581367 Gpnmb
74581368 Gmpr
74581369 Otc
74581370 Ubash3b
74581371 LOC434002
74581372 Ccdc37
74581373 Akap13
1407 Sez6l2
74581376 Sema3d
74581377 Egr3
74581378 Cnnm2
1411 Rbm11
74581380 Etfa
1413 Arhgap17
74581382 Znrf1
75826567 Chrna5
75826568 Chrna9
1418 Shh
1420 Slc6a9
74357561 1110007C09Rik
1422 Sox9
1424 Tcf7l1
71670680 Ovgp1
74581394 Pltp
74581395 Hpcal1
74581396 Net1
74581397 Sh3gl2
1431 Tsc2
74581400 Ptchd4
1435 Ucn
112194293 Park7
1440 Vil1
74581410 Mettl24
1444 Wnt4
73925719 Adamts8
77874598 Kcnj2
71717105 Pmfbp1
1448 Akap2
1450 Dlx5
75773511 Scg5
77874604 C1ra
74581421 Allc
77874606 Thrsp
77874608 Cyp39a1
74581425 LOC277860
74581426 Lrrc1
1460 Ifngr2
77874614 Tm7sf3
77874616 Prune2
71016692 Rap2a
1466 Zfp788
1468 AI606473
74581440 Ostn
74581441 Palm2
74581442 Palmd
70927811 Fam84a
1476 Cdkn2b
74581445 Pcdhb18
79567709 Ptgds
74581447 Pex5l
74581448 Plekhh2
74581449 Slc2a12
74581450 Slc8a2
74581451 Sh3rf1
1484 Dmbx1
1486 Dok4
1488 E130016E03Rik
70927826 Ckb
70927827 Lamp5
1492 Ephb3
70927829 Pqlc1
1494 Etv3
70927831 Dusp14
1496 Foxd3
74988452 Cyb5r1
112649690 Gpr35
1500 Fzd5
70436317 Slc17a7
71670693 Prkcq
1504 Hes6
1506 Kif15
74658043 Dgki
1508 Lmx1b
74988454 Aacs
1510 Mtf2
1512 Myb
72007932 Ufsp1
1514 Ndel1
71670695 Shb
1516 Pdgfa
1518 Pla2g12a
1520 Pqbp1
74988451 Cblb
1522 Prrx2
75081205 Gdf1
1526 Dennd5a
74988457 Ifit2
75081208 Dhrs7
75081209 Ddt
75081210 Slc17a7
255 Chd2
1532 Setbp1
71670698 Tbc1d1
1534 Sprr1a
1536 Srprb
1538 Suv39h2
257 Chrd
71670700 Tmem25
1547 Ube2g2
72119655 Chrna3
71670701 Trappc1
1553 Frat2
1555 Nfib
74988462 1110037F02Rik
74425563 Plxdc2
1561 Plxnb1
1563 Pou2f1
1567 Rgs16
1569 Rgs4
1416 Sh3bgr
1571 Slc20a1
71670705 Txn2
1577 Abhd11
1111 Wif1
74658055 Adap1
1581 Fgfr4
1583 Gpr84
1585 Grin1
1587 Rwdd2b
74511966 Gm520
1591 1200016E24Rik
1593 Srpr
74511967 Serpinb1c
1597 Slc36a4
1599 Asb6
1601 Bcl7b
1603 Cdkn2d
1605 Cx3cr1
1607 Zfp423
75041433 Fmnl1
1609 Foxb2
1611 Gba
1920 Slc27a4
1613 Gcgr
1615 Gli3
1617 Gpc4
1619 Gpr108
1623 Hoxd9
2509 Ptpn18
1629 Kctd5
1631 Cenpf
1633 Sh2b3
1635 Letmd1
1637 Ufc1
79591541 Cd24a
1639 Slc25a22
1643 1700086L19Rik
1645 Slc39a5
1647 Sppl2a
74750009 Ube2b
1649 Ostc
1651 Cmbl
1653 Srfbp1
1655 Ppp2r2b
1657 Gpt2
1659 Abtb1
1661 Aplnr
74958463 Tsc22d2
112647104 Mcl1
1667 Vash2
70562070 Npffr2
1673 Brd8
71717143 Spg21
1677 Ankrd54
1679 Cacnb4
1681 Casp9
1683 Cdkn2c
1685 Chuk
71924077 Myt1
1689 Copa
1691 Cpe
74819229 Prdx4
74819230 Tomm20
74819231 Prr15l
74819232 Hdlbp
1697 Nomo1
74819234 Cxcl13
283 Cnr1
74819236 Cwh43
74819237 Rpl23
74819238 Eef1g
74819239 Fn3krp
74819240 Aldh2
74819241 Ttc27
74819242 Parp8
74819243 Ndufs8
77414917 Nudt2
1709 Erbb2
74819246 Fam210a
1711 F2r
74819248 Wdr47
74819249 Cd4
75081394 Tmem47
74819251 Adm
75081396 Plcl1
1717 Folr2
74819254 Casq1
71670729 Sh3rf2
74819256 Il13ra1
74819257 Dffa
74819258 Hs6st1
287 Col18a1
74819260 Sms
1726 Gmfb
1730 Gnaz
77280436 Eya4
1732 Gng11
1734 Gng7
1736 Gpc6
74958537 Il17rd
1738 Gpr85
1740 Polr2m
74658082 Itgav
1742 Dtd1
75077172 Tox3
1746 Hnf4a
74958547 Palm3
1748 Homer1
1750 Ikbkb
73992934 Med26
1754 Kcnab2
71670735 Xk
1756 Kcnc4
74363347 Filip1l
1758 Kcne2
1760 Kcnip1
1762 Kcnk13
1764 Kcnv2
74658086 Myadm
74958566 Mon1b
71670737 Esr2
1768 Lphn2
1770 Mlc1
1772 Mmaa
71247597 Bai3
74958576 Firre
1778 Mx2
1780 Nat6
1782 Ncoa4
1786 Ngfrap1
1663 Slc22a13
1788 Notch3
74819325 Agps
71247614 Gabbr2
74819327 Ppp2r5b
74819328 Lrrn2
74819329 Vezt
71247618 Grik2
74819331 Dach2
1796 Wash
1798 Setd4
1800 P2ry6
1802 Pcbd1
1804 Pde1c
74958606 8030498B09Rik
74819343 Mxi1
74819344 Arl2
74819345 Dad1
1810 Pigt
74819347 Rap1b
1812 Plcd1
1814 Pxn
1816 Raf1
1818 Ramp3
71247644 Htr5b
1822 Rgs3
1669 Zfp280c
74958626 Slc2a13
70613979 Extl2
1830 Sfrp2
12 Ndufv3
1832 Siglece
70928169 Stx12
1834 Six1
1836 Slc12a8
1838 Slc29a1
1840 Slc2a3
1842 Slc34a1
1844 Slc38a1
74958645 AI852640
1846 Slc39a14
71247671 Ankrd6
71247672 Egr1
1850 Slc6a6
1852 Slc9a8
1854 Snap25
74958656 Nat8l
1858 Syap1
1675 Clptm1l
70928196 Adk
2249 Galm
70928198 Dbn1
70928199 Cast
1864 Syt13
71717125 Zbtb16
75774681 Hmox2
1868 Tbl2
1870 Tcea3
1872 Tcerg1
1874 Hnf1b
75077262 Hdac7
1878 Fam60a
1880 Pllp
1882 Crlf2
1884 Ubl3
1886 Usp16
1888 Vdac2
77866850 Hspb8
77866852 Tmem176b
1894 Zfp191
74512017 Phldb2
1896 Fcrls
1898 Fam89b
70928238 Car4
1589 Wnt2b
72283795 Ptgfr
1908 Pcdhb17
73512497 Dusp3
1910 Pdgfd
609 Kcnj6
70928248 Igfbp6
70436729 Slc38a3
70928250 Tmc6
70436731 Tspan11
1916 Rxrb
1918 Scnn1g
70436736 Sema3e
70436737 Cap2
1922 Slc4a10
70436739 Fam213a
70436740 Spp1
74819462 Hdac6
74819463 Rsrp1
74819464 Mrpl54
74819465 Efcab6
70436746 Nipal3
70436747 Chrnb3
1932 Mlxipl
70436749 Tmem132a
1934 Wbscr17
70928271 Alcam
1936 Wnt7a
79488913 Cnp
1938 Obfc1
74819475 Lrtm2
74819476 Ap2a2
1944 Tsen34
71717188 Dio3
1946 Micu2
1948 Grina
74819486 Uba6
79488927 Tcf4
71250313 Ccdc184
79488929 Acvrl1
1954 Smg9
79488931 Satb1
70928292 Pea15a
70928293 Rtn1
70928294 Mad1l1
74988529 Ccni
1960 Tmem218
74819497 Dbnl
74881867 Sik3
79488939 Igsf5
1964 Fam136a
1966 Trap1
1968 Snhg7
1970 Rabep2
70928307 Fam26e
1972 Smarca2
1974 Igfbpl1
73769289 Enpep
79488953 Adcy7
1978 Ccdc137
70231995 Osbpl3
1980 Olfml2b
74819517 Rcor1
1982 Klhl24
70231999 Mterf2
70232000 Prph
1986 C2cd2
71924087 Ank2
1988 Soga3
1990 Aph1a
72283809 Strip2
70928328 Galnt18
80343372 Kcnj6
1994 Pde4dip
1996 Fam195a
72283810 Doc2b
1998 Rfx7
70562125 Scnn1a
2000 Dlx6os1
77414654 Pea15a
70928338 Arg2
70928339 Enox2
70928340 Cplx3
75043150 9830166N24Rik*
2006 Ankrd33
2010 Ldlrap1
75084111 Ppp1r3b
2012 Abhd8
1158 Atp5o
2017 Adcy7
74819554 Kcna6
2019 Adrm1
2021 Agtr1a
73929858 Slit3
2023 Clrn3
70928360 Samd11
2025 Aldh1a2
2027 Ankrd10
2029 Anxa11
2031 Apba2
71064278 Rcan2
2033 Apbb1
73635975 Slc8a1
2037 Aqp9
70928374 Gadd45b
1705 Eps8
340 Ddx5
2043 Arhgef2
2045 Arntl
2047 Asgr1
70928384 Anxa2
70928385 Ssbp2
2051 Ciao1
2053 Axin1
2055 B4galt6
2057 Barx2
74819594 Boc
2059 Gpr146
70928396 Tacc3
74819245 Kcne4
2065 Bmp7
2067 Mpc1
2069 Btd
2071 Tspo
2075 Sdccag3
2077 Cbr3
2079 Cct8
321200 Dlst
2083 Cdh4
2085 Cdkn1a
75081393 Col6a3
74273116 Slc26a10
2091 Cer1
2093 Chga
70805955 Usp46
2095 Chrm3
2097 Chrnb2
321203 Cnot7
2101 Cmtm4
74819252 Mylk
323590 Mocs1
2107 Cldn12
2109 Cnih2
75081397 Ssb
74047840 4833420G17Rik
74819651 Fam212b
74819652 Gpx2
1707 Eps8l1
2119 Crat
74819255 Atp1b2
2125 Cr1l
2127 Cryaa
2129 Csrp1
2131 Cstf2
74724750 Hrh2
2135 Cx3cl1
75084132 Recql4
2141 Dbr1
2143 Eif4a3
112650336 Drd4
74819681 Gbas
2147 Dip2a
2149 Dkk1
74819686 Smu1
74819687 Nubp1
74819688 Slx1b
1724 Aldh1l1
74819690 Clta
2155 Dok2
74819692 Blnk
2157 Donson
74819694 Fxyd5
74819695 Stmn2
74819696 Tgfbr2
2161 Dph2
2165 Pigp
2167 Dtx1
2171 E2f1
2173 E2f6
2177 Efemp2
2179 Ei24
2181 Eif2ak3
2183 Eif5a
2185 Elk1
2187 Epha4
2191 Ephb6
79567753 Mtmr7
2193 Eps15l1
71924386 Dlgap1
2195 Erf
71247617 Grid2
79490073 Pcp2
73769327 Neurl1a
2205 Fbln1
2207 Fbrs
74658160 Gpr162
2210 Fbxo2
76097699 Golga7b
2212 Fem1a
76097701 Tmem179
247974 Zscan22
72338724 LOC381076
2218 Fhl1
2220 Fkbp10
2222 Fkbp6
2224 Flt1
2226 Fnta
75081005 9330182L06Rik
2230 Foxg1
2232 Foxi1
71250292 A230065H16Rik
2234 Frs3
73615550 Adra2a
75749783 Parm1
2241 Fzd4
73992715 Cd63
2245 Gabarap
2247 Gabrb1
73615560 Cacng3
70813897 Anxa1
70813898 Ivns1abp
2251 Gas1
2255 Gja1
2259 Gjb5
70813908 Spint1
73615573 Iqgap2
2263 Gnai2
2265 Gnat1
2267 Gnpnat1
74047866 Meis1
67936016 Tfam
2271 Gpr37
73771233 Nwd2
70813922 Akr1b3
73771235 Necab3
70813924 Kctd9
70813925 Parvg
70813926 Tppp3
73771239 Dusp10
73771240 Mafb
70813929 Tmem176a
2115 Nudt21
2285 Hdgf
2287 Hes3
2289 Hes7
2291 Higd1a
73520966 Baiap2
2293 Hip1
2295 Hmga1
2299 Hoxb13
2303 Hax1
2305 Hs3st1
2307 Hunk
2743 Zfp358
74047915 Atp6v1d
2313 Igf2
2315 Il11ra1
2317 Il17rb
2319 Il17rc
2321 Impg1
74881879 Focad
2323 Irak1
2325 Irx1
73907500 Rxfp2
67810538 Hsf1
2331 Kdm4b
74882585 Fbxo34
2333 Jun
2335 Junb
2337 Kcne3
2339 Kcnj15
2341 Kcnj16
2343 Kcnj8
2347 Kdr
73929869 Stim2
2353 Kif5b
1715 Fgf15
2359 Kpna4
73930821 Kalrn
112650553 Dhrs3
2363 Aff4
2367 Lat
2369 Ldb3
2261 Gjb6
2371 P3h1
2373 Leprotl1
2375 Lhx2
2377 Lhx4
2379 Lhx8
2383 Lmo2
2385 Lmo4
2389 Madcam1
2391 Pam16
2393 Map3k7
2689 Tnfrsf1a
2397 Mapk13
234 Cdh13
2399 Mapk14
2401 Mapre1
2403 Mcm5
1766 Ldlr
2407 Mif
2409 Mtf1
74047713 Fat3
2411 Mtpn
2413 Mtrr
71924374 Clvs2
2417 Kat8
2419 Gm953
2421 Ndufs1
2423 Nfix
2425 Nfkbie
2429 Nkiras2
79907904 Vim
2434 Nr2e3
79587715 Calca
2438 Nrf1
70805895 Eepd1
79587720 Bdnf
2442 Nudc
2444 Nudt9
79587726 Sod1
2448 Olig2
2450 Omg
2452 Optn
2454 Otp
74819689 Tubb2a
2460 Pafah1b1
2462 Park2
2464 Pax3
1776 Mtss1
2466 Pax9
72008120 Btg3
2468 Pcdhb20
73615782 Masp1
1446 Szt2
2472 Pde4b
2474 Pdk3
2476 Pdlim2
2478 Pgrmc1
70805935 Ctnnbip1
2480 Phox2b
73615793 Nr4a3
2483 Pkig
2485 Plg
2487 Plk1
71020431 Gpc3
2491 Pnrc2
73615804 Pgr15l
73615805 BC100451
74724762 Krt80
2495 Pparg
2497 Ppm1a
2499 Ppm1g
2501 Pnrc1
2503 Psmd4
2507 Ptn
418 Fabp3
2511 Pvrl4
70928373 Vopp1
2515 Pycrl
2517 Ran
2519 Rangap1
74047908 Ppp2r5d
2523 Rela
74724763 Id3
2525 Relb
2527 Traf7
2529 Rgs14
74958587 Sned1
73929875 Fbln7
2533 Rhbdf1
2535 Rbm38
2537 Rora
77371845 Gpr146
2539 Rorb
2541 Ryk
75147758 Rasal2
75147759 Ttbk2
75147760 Nrn1
75147761 Igsf9b
75147762 Nos1
2547 P3h4
75147764 Kcnk2
75147765 Kcnc2
75147766 Kcnd3
2551 Scarb1
75147768 Col5a1
75147769 Cdk8
71247615 Gpr6
2557 Frrs1
2559 Serpinf1
1792 Nr4a1
73992707 Trpv4
73992708 Satb2
73992709 Tmem178
73992710 Plekha7
73992711 B3gnt5
71015195 Endod1
2569 Sla
70560266 Fnbp1l
2571 Slc11a2
73992716 Cdr2
74819330 Strada
73992718 Cntn4
73992719 Fat3
73992720 Dlgap2
2577 Slc16a14
2579 Slc16a4
70560276 Ank1
2581 Slc16a6
70560278 Gpr165
2583 Slc18a1
2585 Slc19a2
2587 Slc25a1
2591 Slc25a5
70560288 Npsr1
70806049 Bdh2
2595 Slc26a7
2597 Slc26a8
2599 Slc2a4
73787946 St6galnac5
2603 Slc31a1
2605 Slc35a1
2607 Slc35a2
73787952 Dgkb
2609 Slc35a3
2613 Slc35b2
2615 Slc35c2
2617 Slc38a4
73992763 F5
2621 Slc5a1
74047925 Ank3
2625 Slco4a1
2629 Srp54a
2631 Ssr1
70560328 Brs3
67881545 Rfxank
74047927 Rplp0
2637 Stat3
2639 Stc1
67881552 Smpd1
67881553 Clns1a
67881554 Gpr89
2643 Aurka
70560340 Cacnb3
70560341 Cacng5
70560342 Cckbr
74047878 Tsg101
2649 Syt11
71247631 Grm4
2653 Sytl3
2655 Taf13
2657 Tat
2659 Tcf19
2661 Tfap2a
2663 Tmbim6
72129242 Tspan32
73718057 Scg3
73788010 Necab2
2667 Tgfb3
74819346 Ssrp1
70927812 Brinp3
2671 Thpo
2675 Med24
2681 Tle3
70634108 Lrrc49
75650865 Neurod1
2687 Tnfrsf12a
73788032 Nts
71670485 Lgi2
2691 Traf2
2693 Traf5
70634118 Prss23
70814343 Mob3b
70814344 Atf3
112194721 Myc
73788043 Otof
73788044 Otof
2701 Nprl2
2703 Tyr
70634129 Dusp18
73929608 Slc5a7
2707 Unc13c
75041430 Cubn
75041431 Six3os1
75041432 Fdft1
2713 Usp25
2715 Ezr
2717 Dcaf4
77925023 St8sia2
73616033 Esrrg
75041442 Hspa1l
75041443 Il10ra
73616036 Mgat5b
73616037 Lmo3
2727 Wnt11
2731 Wnt3
75041453 Insig1
75041454 Lrfn2
2735 Wnt6
2737 Ywhab
2739 Ywhae
2741 Zbtb18
75041463 Lrrc4
75041464 Lrrn3
2747 Ltn1
77867708 Gltscr1l
73788094 Slc30a3
356488 Bhlhe40
75041473 Ndst3
73788105 Slit1
73992908 Fndc1
73992909 Gas7
73992910 Grem2
73992911 Gucy2c
73992912 Hc
75041491 Slc36a1
73992916 Rasgrf2
73992917 Scml2
73992918 Slitrk6
73992919 Smug1
73992920 Opcml
73992923 Bsn
73992924 C230030N03Rik
73992925 Tmem72
199390 Arf1
112195815 Clec11a
199392 Mef2a
199393 Tnfrsf11a
73992930 Cnnm1
73992931 Cntnap1
73992932 Col4a5
73992933 Cox4i1
74363335 Prune2
75041511 Tcerg1l
77925097 Tnfrsf25
73992938 Cyld
77925099 Slc44a1
73992940 Mroh1
73992941 Lppr4
73992942 Mylk3
73992943 Ablim3
73992944 Dcbld2
73992945 Dgkh
73992946 Snhg11
73992947 Eif5a2
73992948 Eml1
71924387 Kcnip4
75041526 Rgag1
71247657 Ryr1
75041528 Rapgef6
71717618 Tdo2
70634234 Csdc2
70634235 Rfx3
70634236 Socs6
71247658 Trpc5
72129247 Prkcb
74363349 E130012A19Rik
75041538 Lonrf3
73636098 Htr2c
2549 Scand1
75041548 Dock6
75041549 Nipa2
75041550 C1qtnf3
1458 Gtf2h4
70634256 Gtf2h2
75080152 Gnrh1
367 Ripply3
72108822 LOC433254
77925143 Cirh1a
72108824 Glra1
72108825 Kcnc1
75041562 Mex3d
276271 Pyurf
73994714 Kcna1
74425517 Atat1
276272 Plcd3
74363355 Ctnna2
73521803 Ptprj
75041573 Rufy4
75041574 A130014H13Rik
75041575 Dscaml1
71015205 Iyd
74363356 Fat4
74047443 Dock10
74363357 Hspb1
75041585 Qrfp
1924 Trip4
70634296 Bag2
70634297 Pcgf6
70634298 Spock3
70634299 Hkdc1
70634300 Cyp27a1
75934525 Rcsd1
75934526 Eef1b2
75934527 Pcdh1
75934529 Rnasek
73521804 Pvrl3
74724775 Tppp
70634310 Cish
96 Ap2b1
72109337 Gm5607
73817427 3110035E14Rik
248329 Med30
73520987 Hsd11b1
77874596 Thbs4
321201 BC005537
72081514 Serpina3k
72129251 Myl4
79568020 Zbtb20
71920509 Tmem106b
71920510 Stk32a
71920511 Phactr1
71920512 Itgb5
71920513 Mybpc1
71920515 Rftn1
71920516 Kirrel3
71920517 Wscd1
71920518 Sertm1
71920519 Sertad4
71920520 Mpp3
71920521 Sobp
72119593 Fn1
72081515 Tanc1
1860 Syngr3
70634395 Dkkl1
70634396 Fabp5
75077213 Parva
1862 Synpr
70634406 Ublcp1
70634407 Gadd45g
74988700 Gpr125
73520967 Fam46a
77332706 Hapln1
645 Llgl1
70634417 Lgals1
74882206 Fam50a
75080180 Ccdc171
70813896 C1ra
112651200 S1pr5
79588290 Fgfr1
70743883 Depdc7
79588292 Dtnbp1
79588294 Aldob
73520972 Cdca2
75651149 Gpr137
72081517 Igsf3
70743886 Chgb
112646460 Cd97
70743887 Fgfr1op2
71670707 Ube3c
74274689 Bhlhb9
70743888 Gprasp1
73520977 Layn
74882216 Tmem131
1193 Dbp
75233110 Tex40
71836833 Ina
70743895 Uap1
79490064 Calml3
227160 Sc5d
79490066 Cdh13
515 Gng4
73520985 Hpcal4
73788441 Map3k15
73788442 Map3k15
73788443 Sparc
70743898 2010300C02Rik
79490078 Zfp189
77874602 Sostdc1
77371769 Bcar3
227163 Flcn
77340494 Adrb1
73788454 LOC433228
74988721 Ubr3
73520988 Hspb3
77414919 3830406C13Rik
227165 Cnppd1
79568051 Ndrg2
79907893 Dbh
75038483 Nod2
73788474 Glra3
73520991 Itga7
73520994 Kit
79490110 Apoe
79490112 Pfkp
70743904 Nfil3
79490114 Clu
79490116 Gpr139
79490118 Kcnj2
73520993 Kctd12
79490120 Cldn5
79490122 Camk2a
74583117 Odc1
74583118 Eid1
74583119 Prkar2b
73521813 Ppp1r2
73636037 Sstr4
70743907 Elmo1
74583129 Slc4a2
527 Grik1
79907934 Crhr2
79907936 Cxcl12
74583139 Bend5
79907941 Dyrk1a
74818622 Ndufs2
74583149 Vav2
74583150 Hist3h2a
74583151 Sirpa
70562124 Rxfp1
74583161 Lrsam1
74583162 Rhobtb3
74583163 Pik3r3
77332098 Adamts15
71836837 Bcl11a
70634139 Ttc39b
74583174 Arhgap33
74818623 Ccdc91
61210 Adipor1
73636111 Scn4b
74988739 Zfhx4
1902 Gm6740
112651416 Thbs4
71924502 LOC432748
75041513 Tiam2
74425544 Slc43a2
74583197 Cdo1
71249055 Foxo1
71249056 Foxp1
71249057 Plagl1
74583203 Ube2h
77414160 Gng2
71249067 Pou6f2
71249068 Rfx4
71249069 Thrb
74988744 Brk1
71249079 Glt8d2
71249081 Ttc9b
71249082 Megf10
71249083 Ube2ql1
71249084 Limch1
75934914 Cyp51
75934915 Capns1
75934916 Ank
75934917 Rps4l
75934918 Rap2b
75934919 Zfyve28
75934920 Rpl32
71670715 4921531P07Rik
72109262 Gm1441
1912 Ramp1
75826557 Chrm5
77332708 Hba-a1
1914 Rnf128
73931405 Gxylt2
72109295 Ptpn3
72103807 Npy1r
74988757 LOC433311
74274688 Arl5a
73521819 Rgs9
72338695 Pcdhga5
72338696 Gpr83
72338697 Casr
72338698 Sim1
72338699 Calcb
70436738 Rab3b
77874610 Clic5
75080238 Tekt1
73992915 Pard3
75651160 Ccdc3
74819460 Lpin1
77868319 Lamc1
356093 Akr1e1
74272036 Chchd2
74272037 Map2
74272038 Draxin
74272039 Rrm2
74272040 Hist1h1b
74272041 Cxcl14
74272042 Cdhr3
74047745 Srgap1
70436744 Slc7a14
74425549 Ilf3
75041492 Stra6
74272053 4933427D14Rik
75651161 Tmem91
1930 Best2
70813909 Stard5
72109379 Adarb1
77866868 Ntm
74272906 Sh3kbp1
72109389 Ighm
70928269 Klk10
1401 Pknox1
71836844 Atp1a1
75651162 Zfpm2
70928270 Gap43
72109399 Wdfy2
75774684 Dync1li1
79567501 Adcy2
72109410 Adora2a
226 Cd81
79568047 Caml
74425551 Elovl6
72109423 Rnf13
75081395 Fam84b
1940 Zbtb48
70615598 Gpd2
72109445 Dlg4
322956 4930506M07Rik
322962 Iffo2
72104173 Pde6g
322964 Skiv2l2
72109467 Brinp1
112645384 Eva1c
72109489 Ace
75042226 Dpy19l1
585 Icmt
73521825 Timm10
74819487 Axin2
75042236 A230097P14Rik*
75042237 Fam46a
75042238 A530057D15Rik*
75042239 Cdkl5
75042240 Nrxn3
75042241 Abcc4
75042242 Rims3
75042243 Fam196a
75042244 Fam196a
75042245 B830028H17Rik*
75042246 Cacna2d1
75042247 Adra1a
75042248 Dok1
75042249 Myo5c
75042250 Gbe1
75042251 Hsp90b1
75042252 Acot13
75042253 Tomm34
75042254 Glg1
75042255 Rdh5
75042256 Lrrc16a
75042257 Elmod3
75042258 Wrap73
75042259 Nkd2
75042260 Tk2
75042261 Qdpr
75042262 Pdia5
73521826 Trp53i11
75042264 Cth
1956 Pttg1ip
75042266 Acsl3
75042267 Arid5a
75042268 Nop10
75042269 Lcat
75042270 Scara3
79488933 Anxa5
74640879 Syne1
74640880 Syne2
74640881 Synpo
74640882 Epha1
74640883 Gucy2f
74640884 Mm.26272
73521827 Zfyve9
79488937 Rora
104826 Bcap29
74512028 A830036E02Rik
75988600 B3gnt2
2575 Slc13a3
71358663 Syt16
70928304 Col9a3
355882 Fbxw2
355883 Usp11
355884 Vps35
70928306 3632451O06Rik
355886 Pls3
355887 Ncaph2
355890 Fh1
355892 Dag1
72109633 Chst2
75041501 Sulf1
544326 Phyhipl
544327 Serpinb1b
544328 Phyhip
61002 Limk2
75043138 5330421K23Rik*
70301087 Kcnab1
75041516 Lnx2
73992926 Nt5dc3
79488955 Avpr1b
70231996 Sqle
75043139 Tead1
70231997 Gal
79556599 Fgf1
74272466 Atp9b
1984 Clic6
75081003 Snx31
74881285 Cdh11
71587781 Arpp21
75043141 Tceb3
72008305 Lypd1
77868713 Hcrtr1
74658245 P4hb
71587783 Nrxn3
1992 Fam114a2
73992929 Cidea
72104183 Dpp10
73592526 Crym
73592527 Celf4
73592529 Cdh10
73592530 Igfbp5
73592531 Pthlh
73592532 C2cd2l
73592533 Dhx40
73592534 Fxyd6
73592535 Dgcr6
73592536 Fxyd7
73592537 Tmem65
73592538 Myo5b
73592539 Trpc2
73592540 Col4a2
74272477 Fez2
74272478 Gdf11
74272479 Nrp1
74272480 Ppfibp1
74819536 Impg2
77278951 Gsta4
2002 Hectd2
356082 Cct2
356083 Rbm18
74425564 Serpinb6a
77278965 Mylip
356086 Abcd1
356087 Homer3
356088 Pias2
356089 Nxf1
356090 Gaa
356092 Nsf
77278973 Serinc5
77278975 Rec8
77868800 Rps5
77278977 Slc9a7
77278979 Cyp2j6
544516 Slc25a25
77278981 Pdgfrb
74641159 Gca
544520 Gpm6b
74641161 Mm.86217
74641162 Osbpl8
74641163 Stk33
76115724 Tmem63c
76115725 Gabrb2
76115726 Casq2
76115727 Ckm
76115728 Got2
76115729 Hadhb
74425565 Fam49a
643 Lix1
76115732 Ipo5
76115733 Ltbp3
76115734 Map1a
76115736 Nin
77279001 Tac2
76115738 Ntrk3
76115739 Psme1
76115740 Prdx5
76115741 Prosc
76115742 Rasgrp1
76115743 Rasgrp2
76115744 Slc9a6
73520983 H2-Eb1
75081398 Zfp330
2039 Ar
74272052 Map7d2
74641219 Dnm3
323396 Cap1
74272054 Adam19
75651128 Adamts18
71249739 Rassf8
71249740 Mical2
71249741 Ism1
71249742 Krt222
653 Lrp5
71249744 Ppp4r4
71249745 Dsel
71249746 Arsj
71249747 9530091C08Rik
71249748 9630033F20Rik
71249749 Ptchd1
71249750 Zmat4
357096 Pop4
321196 Eif3j1
131 Avp
71020389 Pde8b
71020390 Stt3b
657 Map4k3
71020392 Tdg
79488957 Stk17b
77925095 Fzd4
74749928 Rpl8
74641266 Nacc2
659 Mapre2
70928361 Cntnap4
77413696 Serinc1
77413698 Nfix
74641294 Gm5433
74819565 Iscu
71020432 Kcnd2
71020433 Opn3
75043149 Slc38a1
74641304 Pou4f1
77413700 Enpp2
74641306 Glce
74641307 Cox6c
74641308 Zfhx3
74641309 Hmx3
74641310 Kcnh3
74641311 Ldb2
74641312 Ak4
74641313 Gpr17
74641314 Rab27b
74641315 Atp6v1a
74641316 Avpr1a
74641317 Aars
74641318 Htr3b
74641319 Pcyt1b
74641320 Ctnnd1
74641321 Cdyl2
74641322 Gpr156
74641323 Ttpal
74641324 Celf3
74641325 Impa1
74641326 Cited1
74819693 Lyz1
356284 Tmem33
356286 Clk2
356287 Phf1
79908802 Limk1
72339397 Dirc2
73520984 Hint1
72340129 Cmklr1
79908808 Mecp2
75041514 Tmc1
74819575 D11Wsu47e
79908817 Notch3
677 Mtif2
79908833 Tenm3
61411 Tnnt3
61413 Gnb1
73992939 Arhgap39
73817426 Zfp804a
79908848 Snca
79556595 Lpar1
67809268 Ogfr
79556597 Rorb
71717115 Tnc
79556601 Efnb3
79556603 Kif5a
79556605 Dkk3
79556607 Pitx2
323585 Map2k5
323586 Rer1
79556611 Galp
323588 Eif2d
323589 Atp5s
79556614 Ddc
2049 Zak
323592 Dynlrb1
323593 Sdccag8
79556618 Cux1
323595 Uqcrb
323597 Dctn6
79556624 Reln
77869074 Cck
79556628 Arhgdig
79556630 Mc4r
321202 Mvp
79556632 Syt12
74047913 Pi4k2a
79556634 Ctgf
79556636 Aldoc
79556638 Pus7
71587845 Thrsp
79556640 Cuedc2
79556642 Gja1
79556644 Cd44
79556646 Lrp5
79591611 Grm2
77869097 Col6a1
79556652 Sox10
79556654 Pvr
73732144 Entpd2
73732145 Isoc1
79556658 Frzb
73732147 Trim3
79556660 Wnt5a
73732149 Cd8b1
73732150 Fam81a
73732151 Notum
73732152 P4ha2
73732153 LOC545854
73732154 St3gal6
79556668 Tspo
79556672 Calb1
79556674 Uchl1
70928395 Rps12
71250311 AI450948
77869128 Snd1
77869130 Usp11
71717560 Ajap1
73817443 Ldlrad4
72339542 Nek7
77869144 Vps35
77869146 Pdxdc1
79556700 Trap1
79556702 Bmp4
79556704 Plp1
79556706 Gad1
72339555 Cables2
79556708 Htr2b
72339557 Tcf7l2
79556710 Sp3
79556712 Ngb
79556714 Sssca1
79556716 Hopx
1162 Bhlhe22
79556722 Aqp4
74272892 Arhgef28
74272893 Rgs6
74272894 Scube1
71247616 Gria3
79556736 Gdap2
79556738 Pvalb
74657923 Thbs3
74657924 Slc22a8
544901 Clptm1
544902 Tecr
74657927 Chst14
74657928 Tspan5
74272905 Tll1
356490 Sub1
74657931 Chst10
72077452 Wls
356493 Cops7b
72077454 Coro2b
72077455 Schip1
79589520 Itpka
79589522 Grik1
74272916 Col27a1
74272917 Col5a3
74272918 Iqcj
74272919 Nenf
74272920 Igfbp3
72077466 Atic
72077467 Dgat2
74657950 Pfkfb3
74657951 Msh2
74272930 Papl
70431430 Fryl
72077478 Ephb1
72077479 Cartpt
72077480 Fam222a
1233 N6amt1
67850951 Tcf3
67752111 Flot1
79591721 Tacr1
67850952 Kcnk1
67752116 Vtn
79556696 Mapt
67850953 Apln
74657980 Slc9a3r2
74657981 Ralb
70431435 Mtmr12
321208 Fbln2
74658000 Apba1
112644308 Tmem175
71717078 Hapln4
71717079 Irs4
71717081 Kifc3
71717082 Mest
71717083 Pde3a
71717084 Pdyn
74658019 Arl10
74658020 Sox2ot
74658022 Cdh18
74658032 Sun2
74658033 B3gat2
71358627 Dnajc6
71836875 Rprm
2089 Agap3
77869780 Cdh15
70562043 Gabra3
1229 Mgp
70562045 Gpr161
308056322 Efna1
74658053 Atp13a5
70562055 Gpr155
74658056 Col14a1
70562057 Gpr26
74047778 Dnal4
71717135 Pstpip1
71717136 Rab3ip
71717137 Rasl11b
74658066 Col19a1
76135811 Zim2
71836876 Cox6a2
71717142 Sgcd
80343319 Tgfb1
71717144 Ccser1
72008118 6430548M08Rik
74658076 Dstn
74658077 E330009J07Rik
74658078 Megf9
74658079 Exosc8
74658080 Fscn1
74658081 Nxpe3
80343330 Stc2
74658083 Mela
74658084 Mmp14
74658085 Msn
80343334 Kiss1r
74658087 Mtmr6
74658088 Nt5m
74658089 Kifc5b
74658090 Me2
74658091 Gldc
71717165 A4galt
75084080 Plek
80343346 Edc3
2099 Cmtm3
80343348 Pxylp1
70562103 Kcnh7
75084090 Plxna4
79567499 Fgf12
2329 Irf9
74658110 Csnk1a1
74658111 Ccdc92
75043136 Stard9
75043137 Atp11a
70562114 Kcnq3
71717187 Fstl4
75043140 Trps1
75084101 Tnfaip3
75043142 S1pr1
75043143 Arl3
75043144 Tmem11
75043146 Cyb5a
75043147 Bag1
75043148 7420498E04Rik*
71717602 Wbp5
70562126 Trpc6
75043151 Rnf152
75043152 Srsf9
75043153 Cd33
75043154 Rbmx
75043155 Serpina3n
75043156 Shisa2
75043157 Rdh14
75043158 Prkacb
75043159 Rb1cc1
75551460 Chrna2
74658140 Sdhb
74273117 Pitrm1
74273118 Elovl5
74273119 Hexim1
74273120 Arc
74273121 Cnksr3
80343394 Dok5
75084131 Rarres1
80343396 Ankrd24
77869798 Acat2
75084134 Serpina1e
75551463 P2rx6
71836879 Prss12
80343407 Cyr61
75084144 Serpinb2
75084145 Slc35f1
67752306 Serinc1
322963 Cep78
67752308 Cplx1
67752309 Matr3
75551465 Chrna1
79591395 Cacna2d3
74658171 Pomgnt2
75084156 Slc35f3
74658173 Ndufa10
74658174 Ipo4
74658175 Sptlc2
71250305 A530079E22Rik
71250306 Sowaha
71250307 Acvr1c
71250308 Styk1
71250309 Crtc1
71250310 Amigo2
422 Fbxl5
71250312 AI593442
75551468 Gabrb3
71250314 Suco
71250315 Aim1
71587899 Rph3a
73930819 Tnr
75551470 Gabrr1
323397 Pdha1
73930820 Acyp2
2117 Cpt1c
73930822 Lepr
324008 Siae
112644522 Wnt1
73994667 Fras1
324012 Lum
73994669 Gpc1
73994670 Grsf1
73994671 Ids
73994672 Il1rapl2
71717619 Qrfpr
71717305 Galnt16
74658238 Micu1
74658239 Exoc4
74658240 Pex13
74658241 St6gal1
74658242 Triap1
74658243 Bcl2l11
74658244 Eps8l2
77869814 Ssr2
74363334 Shisa9
74658247 Neil3
74363336 B230312C02Rik
74363337 Fam160a1
74363338 Ttll6
74363339 6820408C15Rik
74363340 Zfhx2os
74363341 Arl15
74363342 Foxred2
74363343 Irs2
74363344 LOC433727
74363345 Zdhhc23
74363346 Ccdc88c
112202195 Pth1r
74363348 Fam184a
71587832 Car10
74363350 Ltb
74363351 Pvrl1
74363352 Sall3
74363353 Ckap2
74363354 N4bp2
73994715 Lyst
73994716 Lzts1
73994717 Mal2
73994720 Map3k5
73994721 Mapk4
544899 Atxn10
71020445 Rgs8
356849 Yme1l1
356850 Psmc2
356852 Nxph3
356855 Zfp790
356856 Zfp810
356857 Ptbp1
356858 Gde1
356859 Ap1b1
356861 Ppp6r3
74819671 Prcp
67870081 Sgsm3
73930840 Camkk1
71358553 Rasgef1b
74273307 Gss
74273308 Sod3
73930842 Jdp2
73931408 Mlip
112194079 Itfg1
75651163 Fezf2
69120555 Cst3
71358557 Nrsn1
79587728 Ly6e
79567815 Wnt7b
293038 Ola1
121091 Ptprcap
80517217 Oprk1
71670489 Maob
71717451 Kcng3
74819683 Slc35f2
782 Pde1a
73519704 Ntsr1
73519705 Magi1
74819685 Fam96b
45666 Ube2i
45668 Dbndd2
74750054 Cacng7
75146001 Cdc42ep3
71670490 Ninj1
69120618 Man1a
2151 Dmwd
69120620 Nov
70744517 Fam20a
324210 Ccng1
324211 1700037H04Rik
324212 Dusp1
324213 Pdcl
324214 Ccdc136
324215 Bzw1
788 Pdxk
789 Pelo
112644736 Kank2
74819691 Sart3
976 Slc5a3
71015813 Lyve1
75888748 Grin2c
791 Per1
72340108 Lxn
72340109 Ptpro
75651181 Lgals1
72340122 Spock1
72340123 Elavl2
72340125 1810041L15Rik
72340126 Lhpp
75888752 Chrnb1
72340131 Gabra1
72340132 Kcnj3
75084453 Neto2
72340134 Rreb1
74819244 Nqo2
112202410 Esyt1
1952 Ergic1
72118960 Sipa1l2
72340145 Ero1lb
75084472 Neu2
75084473 Nhlrc1
75084474 Nid1
2705 Ugp2
75084476 Nog
75084477 Nrk
75084479 Nxph1
67850945 Slco1a5
67850946 Prpf6
71015815 Gpr56
67850949 Tgif1
67850950 Tbp
70431431 Plxna2
70431432 Inhba
70431433 Epb4.1l2
70431434 Mtus2
75084491 Pcsk6
71717582 Kif26b
71717583 Dyrk1b
119504 Pvr
75651192 Csk
77869778 Ap1b1
72340179 Pcdhb16
72340180 Kctd17
75084501 Pak6
72340182 Fam189a1
72340183 6330403A02Rik
71717592 Smoc1
321204 Sar1a
75144655 Gm10635
77869794 Foxa1
75551459 Grik5
357092 Hectd1
75551461 Chrna6
357094 Atp6v0a2
357095 Paip2
77869800 Grin3b
357097 Ing4
75551466 Chrng
75551467 Gabra6
357100 Cd47
75551469 Gabrg1
71717614 Coch
357103 Cyc1
72340208 A830039N20Rik
75551473 P2rx2
75551474 P2rx4
75551475 P2rx5
75551476 Glra4
75551477 P2rx7
75551478 Grik4
75551479 Grin2d
77869816 Prkca
77869818 Lhcgr
77869820 Vstm2b
72340221 A930009E05Rik
71717630 2010111I01Rik
72340223 Prlr
72008491 Ptprt
71717640 Calb1
72340233 Amph
72340234 Avil
75042263 Nek2
72340236 AW046396
2651 Syt4
74958616 Heg1
77869840 Acan
45841 Arxes1
77869842 Tgfb2
814 Plxdc1
75042265 Nrm
75749772 Efr3a
77869854 Apba1
74881157 Usp14
77869857 Paip2
77869859 Pop4
77869861 Gaa
77869863 Cdh13
77869865 Inpp5j
77869867 Prkcq
77869869 Relt
73635976 B4galt5
2433 Nr2e3
71717686 Sh3bgrl2
73635977 Baz1a
74881162 Chn2
176962 Cers4
73635979 Fancd2
655115 Pcp2
823 Pmaip1
74511801 Disp2
72128773 Tmem229b
73929578 Thbs2
73929579 Trp73
73929581 Rln3
73929582 Rragd
73929583 Sez6l
73929593 Sfmbt2
73929595 St8sia3
73929596 St8sia6
73929597 Slc16a2
79591573 Rcn1
112202624 Klf7
72128774 Fam131b
73929607 Slc35d3
75084100 Pola1
73929609 Slc6a15
2199 Ets1
71358616 Celf6
71358617 Thsd7b
75651226 Smpd4
79591579 Plcxd2
71924514 LOC433093
74988551 Mfap1a
75774108 Polr1e
75774109 Tnni1
75774110 Sh3bp4
841 Prkcz
75651231 Eif1b
75651232 Arl6ip5
75774113 Man2b1
74800954 Fbxo9
72472749 Bmp3
72472738 Ptpdc1
71063715 Mapk8
76097700 Tnfaip8l3
72128777 Acan
69153759 Nrgn
1806 Pde4a
74958491 Iars2
252 Chat
72340184 Lrrc38
72007935 Sulf2
73636009 Kctd16
75144703 Cacybp
79591595 L1cam
321206 Actr1a
72472748 Fam43a
855 Ptprd
74273122 Col16a1
71063725 Sez6
67881555 Slc25a36
71836843 Vstm2a
71358638 Mcm6
1687 Vsx2
74819233 Smtn
112645169 Rest
77280309 Pdgfra
77280311 Ccp110
77280313 Bend6
77280315 Mfsd2a
77280329 Arhgef10
77280331 Sox2
77280333 Olfml3
77280335 Arpc1a
77280339 Ugdh
74819235 Taok1
112202838 Mbp
74988334 Slc25a27
71358651 Sema6a
71358652 Far2
72472765 Cerk
362 Rcan1
70436730 Anxa4
73636030 Psd3
76115737 Nol4
112197140 Nfyc
2239 Fzd1
75651223 Sytl4
74881158 Mycl
2440 Ntf3
2357 Kpna1
72472769 Cotl1
74272035 Lipm
71717154 9430028L06Rik
73929868 Snx25
72472770 Cpeb1
73929870 Syn2
73929872 Utrn
73929873 Zfp365
73929874 Cwc25
73636035 Scrt1
77395095 Tmeff1
72472772 Cthrc1
73929885 Gm10413
73929886 Rab43
74724511 Tdrp
77280417 Liph
77280419 Atpaf1
71358662 Sdc3
73929896 Ahcyl2
71717155 Olfm2
77280426 Arrdc3
73636039 Ablim2
77280428 Chn2
77280430 Nav1
77280432 Coro6
73636040 Adarb1
77280434 9630013P03Rik*
74724531 Gpr173
74724532 Adra2b
77280438 Pld5
73615561 Gng13
75651225 Laptm4b
1699 Dner
73615562 Slc6a2
79567505 Mef2c
77280449 Atp2b1
70743234 Eya2
72472779 L3mbtl4
655499 Oxct1
80516310 Crhbp
79591631 Tenm2
72472784 Dapk1
2545 Satb1
72472785 Dcc
74641113 Nav2
67810540 Mef2d
67810541 Mxd4
75084112 Ppp3r2
79591635 Epha4
227540 Syn1
73512186 Ppp1r14b
73512187 Pcdhb9
73512188 Pdxk
73512189 Pcdhac1
73512190 D930028M14Rik
73512191 TC1410973
73512192 Rasa2
73512193 Slc8a1
73512194 Gng12
73512195 Scn3b
73512196 Apbb2
73512197 Sirpa
73512198 Nefl
73512199 Psmc3
73512200 Sparc
73512201 Rnd2
73512202 Kcnj9
72472791 Necab1
73512204 Rgs17
73512205 Psmc6
73512206 Lats2
73512207 Fryl
73512208 Ppp2r2a
74724627 Agtr1b
74881163 Yipf1
74882606 Rap1gap2
73520406 Prkch
79591641 Chrm4
72340194 Miat
72119579 Cachd1
72119580 Kcnj9
74724637 Ackr2
74724639 Celsr3
73771227 Grm8
72119590 Kl
72119591 Ngef
72119592 Mras
74724649 Gpr151
72119595 Etv1
72119596 Itpr1
77280557 Gm13889
80343361 Cebpa
77280559 E330014M11Rik*
112203056 Ptger4
77280561 Hcn1
74724659 Ccr10
79912574 Arfgef2
72119607 Tyro3
80517343 Galr2
74724669 Mchr1
74724670 Gpr37l1
77280576 Tnnt1
77280578 B3galt5
1545 Trim25
77280582 Ly6g6e
70813921 Gch1
77280584 Pantr1
72119628 Adra1d
70813923 Ephx2
72119638 Cacna1h
73771236 Necab3
2277 Gtf2i
72119649 Cacna2d1
72119650 Cacna2d2
72119651 Cacnb2
72119652 Cacng4
71717604 Plch1
72119656 Clcn2
112203270 Kdelr3
2279 Guk1
72119660 Kcnj5
200 Cck
74988249 Yeats2
112194935 Otx2
74724728 Prokr1
74511737 Glp1r
74511738 Chrna7
74511739 Ghsr
72128746 Psd
74724739 Grid1
75041563 Srm
72104172 Tpr
74581379 Stoml2
74511758 Prlhr
72128749 Egr2
75988547 Igfn1
74724760 Htr3a
74511769 Gpr3
70301082 Ache
70301083 Nrxn1
70301084 Rapgef5
70301085 Mgat4c
73512350 LOC436099
73512351 Scube1
73512352 Aldoc
74881264 Zdhhc7
73512354 Tns1
275875 Rnf122
74511780 Prokr2
74724774 Aifm3
74881265 Atp6v1c2
74724776 Ccdc65
73512364 Kcnc2
73512365 Ppapdc2
73512366 Arhgap31
73512367 Sprn
74724786 Plch2
74724787 Adrbk2
79591577 Ltn1
73512377 Asap1
74511804 Entpd3
74819247 S100pbp
74511806 Gchfr
74511807 Gsto1
74511808 Idh1
72104171 Sv2b
75651234 Napb
74581381 Hsbp1
654924 Rap1gds1
74511818 Kif5c
73636087 Inhbb
75988557 Nxf7
72104185 Adssl1
73769308 Hist1h2bg
74511837 Ndst4
74511838 Ntn1
74511839 Prss35
112645600 Nr2f6
79912528 Sox11
411 Extl3
73636091 Clstn2
73512423 Grm5
75774676 Gdap1l1
74511849 Rgs10
74511850 Ppp4r2
79591677 Esr1
74511860 Ptpn3
1551 Wdr18
73512348 LOC434631
79591679 Slc6a4
939 Sema3c
70560265 Heatr5b
74800973 Ptgs1
73636097 Gsg1l
74511880 Daam2
74511881 Gpr176
74511882 Adcyap1
74511884 Rgs4
74511885 Wif1
73636099 Kcnh1
74511895 Rwdd2a
70744324 Atp11b
71670723 Krt9
79591685 Slc6a1
75077152 Prl2c2
74511905 Serpina3c
73512483 Ap3s1
73512484 Slc41a3
74881286 Penk
79591687 Auts2
73512493 Carhsp1
73512494 Lmna
73512495 Adcy4
73512496 Rbpms2
121096 Tnfrsf25
75077171 Polk
73512500 Spns2
73512501 Stap2
121097 Ssr2
1826 Rtn4rl1
71063818 Kctd4
74511935 Bves
74511936 Dmp1
74882656 Manea
72128779 Alox12b
75077191 Nhlh1
75773512 Nicn1
75077193 Bnip3
74511946 Tmem196
79912546 Flt3l
2111 Cnr2
75077203 Dpp6
74511956 Slc35g2
147 Pofut2
953 Sema5b
70301273 Hmbox1
70301274 Prkcd
70301275 Sgpp2
70301276 Rps6ka2
70301277 Ric8b
70301278 Clca3a2
276063 Prrg2
276064 Lzic
276066 Pappa2
276067 Rpl11
276069 Ube2t
75084530 Per3
276073 Map2k4
276075 Pygb
77332671 Csrp3
75084520 Pcp4l1
75077232 Oprl1
74819253 LOC434368
74511986 Sipa1l1
74511987 Tmem145
958 Sept5
75077242 St6galnac3
75077243 Gprin1
74511997 C130021I20Rik
74512007 Cacna1i
71015387 Osbpl10
2647 Supt5
72283789 Tiam1
72283790 Galr1
72283791 Lgr5
72283792 Hrh1
72283793 Kcnmb4
72283794 Lhcgr
963 Serpini1
72283796 Fezf1
72283797 Fam171a1
72283798 Pirt
72283799 Agrp
72283800 Anxa3
72283801 Fam180a
72283802 Btg2
74512027 Rims3
72283804 Cbln4
72283805 Cdh23
72283806 Chd5
72283807 Crim1
72283808 Crlf1
75077281 Rarres2
80516770 Eomes
72283811 Fst
72283812 Fstl5
70616006 Prkar1b
74512038 Cks2
74512048 Nefh
73520818 B230373P09Rik
73520819 Fam102b
112645814 Pwp2
70743885 Ttc39c
75773649 Leprot
73520996 Lgi3
70616005 Tuba4a
112203487 Rgs19
70616016 Alg1
79912572 Oprm1
79591719 Gria1
978 Slc6a3
73718056 Dnttip1
70612723 Cldn1
2105 Clca3a1
70612726 Galns
77413692 Il4ra
70612728 Dhdds
74047845 Meis3
75079765 Ptp4a1
75773698 F11r
75773699 Opn1sw
75773700 Slc31a2
75773701 Spsb4
75773702 Rmdn1
75773703 Mri1
75773704 Ntpcr
75773705 Atp5sl
75773706 Ppp1r1a
32386 Ppp3r1
73512353 Fam189a2
70743841 Mrpl12
70743842 Prdx3
70743843 Plekha2
70743844 Cpne2
112195365 Crlf3
79591729 Ctnnb1
276264 Sbf1
276267 Adamts16
75650860 Vat1l
75650861 Xlr3a
75650862 Sgk3
75650863 Tmem176a
75650864 Dusp6
276273 Rnf219
276274 Senp2
276275 Csrp2bp
276276 Asb8
71670483 Large
70743867 Camkv
70743868 Camk1g
70743869 Tmem41a
70743870 Plekha5
70743871 Nacc2
70743872 Gstm4
75233089 Sypl
73520964 Baalc
73520965 Bace1
70743878 Nab1
70743879 Pnpo
73520968 Gprin3
73520969 Myo16
73520970 Fam163b
73520971 Cd9
70743884 Saraf
73520973 Cdh7
73520974 Cpne8
73520975 Vcan
73520976 Dnah11
73636152 AF529169
73520978 Frmpd1
73520979 Sytl2
73520980 Prox1
73520981 Dpp6
73520982 Gpr126
227159 Wdr5
70743896 Dlat
227161 Acadvl
73520986 Hs6st3
70743899 Camkk2
70743900 Rnf115
73520989 Icam5
73520990 Il17ra
70743903 Fhl2
73520992 Itpkb
70743905 Tspan33
70743906 Zmynd11
73520995 Klhl1
70743908 Ccsap
73520997 Lin7a
73520998 Nptx1
73520999 Nrip3
73521000 Nxph4
73521001 Pak1
73521002 Pcdh11x
73521003 Pcdh7
73521004 Phactr2
73521005 Plxnd1
71064277 Pogk
1291 Syt12
74274683 Gckr
74274684 Lrmp
74274685 Socs5
74274686 Cdc25b
74274687 Unc5b
72103808 Cds1
72103809 Aldh1a1
72472787 Ddr1
72103819 Diras1
72103820 Igsf11
112646029 Lhx9
80517009 Sh2b3
72103830 Cygb
72103831 Car2
72103841 Col4a6
72103842 Zfp57
72103843 Zic1
79591337 Sema3a
72103854 Nell2
79591351 Tbr1
224 Cd44
73635770 Dpysl5
73635771 Rgs2
73635772 Sccpdh
73635773 Stk24
73635774 Lypd6b
73635775 Kcnq4
73635776 4932418E24Rik
80517057 Hmgcs1
80343383 Cdkn1a
75080694 Tns3
71587782 Pdgfb
74749895 Rapgef4
73635786 Fam69b
71358673 Zfpm2
73635796 Filip1
203 Cckar
75988492 Mapk12
73635806 Fam65b
71587811 Trib2
71587812 Sidt1
79591399 Gpr55
73635816 Pacs2
79591401 Met
79591403 Tmem200a
2137 Clip2
71587822 Cacna1g
73635826 Atp10b
77332684 Lypd6
74749941 Sgcg
74749942 Sgcz
74749943 Slc9a2
74749944 Prss39
71587834 Ano2
71587835 Vwa5b2
74881365 D11Ertd759e
74749957 Tnxb
71587846 Stard13
75749384 Ppap2b
75749388 H2-T23
75749390 Hspa4l
71587856 Sema4g
112195601 Aqp2
74749970 Tom1l2
75749399 Cul4a
73616035 Syt17
74749983 Trim23
75749409 Zfp61
72472789 Vwa3a
71587875 Gabrg3
75749413 Abcb6
75749415 Arsa
75749416 Gcg
75749417 Gabre
75749418 Grik3
74749996 Trim37
71587885 Dlk1
71587887 Kcnip3
71587888 Mturn
75651126 Neurod2
73930807 D830030K20Rik
73930808 Syn3
73930809 Sytl5
74750010 Ube4b
74750011 Lrrc74b
74750012 6430709H04Rik
74750013 Galntl6
74750014 Chd3os
74750015 Maoa
74750016 Sparcl1
74750017 Ednrb
75651138 Cntfr
75651139 Bcl6
74750020 Msmo1
74750021 Srp14
74750022 Col9a2
73930823 Epha10
71924066 Zeb2
75651150 6430573F11Rik
71587919 Map4
74750032 Sema6d
73930833 Gm5083
73930834 Tmem130
73930835 Cabp7
73930836 Emb
77869796 Oxct1
73930838 Asic2
73930839 Samd14
71358552 Ahsa2
71587929 C1ql2
71587930 Elfn1
71358555 Rxfp3
71358556 Hhip
75651165 Trerf1
75651166 Ube2e3
75651167 Mfsd6
75651168 Smdt1
75651169 Rprm
75888738 Chrne
73930852 Slc39a6
112646245 Grip2
73930854 Mlec
75651179 Tacc3
75651180 Spp1
75888749 Gabrp
74881134 Stoml1
75888751 P2rx3
74750064 Yjefn3
74750065 Epb4.1l1
71358578 Kcnf1
71358579 Cd200
70743880 Adck4
75651191 Coro1c
74881144 Chst12
74881145 Lrrc3b
74881146 Itm2a
74750075 Grb10
74750076 mCG1049722.1
71358589 Tmem108
71358590 Arnt2
71358591 Asb4
75651202 Dtnbp1
75651203 Kcnj14
74881156 Epdr1
73635973 Adamts19
73635974 Pid1
74881159 Lancl1
74881160 B630019K06Rik
74881161 Wfs1
73635978 C130096N06Rik
112203915 P2ry12
75651213 Chrna6
79591567 Cntn3
79591569 Gabrq
79591571 Magel2
74357563 Pln
73635989 Fndc5
79591575 Xlr3c
75651224 Sirt2
75774105 Alg5
75774106 Tmem206
75651227 Cysltr1
75651228 Slc27a1
75651229 Pir
75651230 Pole4
73635999 Cadm2
71064081 B3galt2
75651233 Vamp1
75774114 Sult1a1
293027 Tmub2
71358628 Gria4
79591593 S100b
293034 Tcf25
72472747 Ankrd34c
293036 Pttg1
293037 Ptcd2
72472750 Fam78b
72472751 C030034I22Rik
72472752 Fam19a4
73636019 Lmod2
71063735 B4galt1
72472761 C230009H10Rik
72472763 Tmem86b
72472764 Cdh9
73636029 Vwa5b2
72472766 Cort
73636031 Ddn
73636032 Gm261
73636033 Fam19a2
73636034 Hrh3
72472771 Cage1
73636036 Sox14
72472773 Nrep
72472774 Dnah9
72472775 Tnks
72472776 Mcu
72472777 D430041D05Rik
72472778 D830029A09Rik
77371767 Fam214a
72472780 Esyt3
79591629 Cdh4
227534 Tcea1
72472783 Daf2
424 Fgf12
80517329 Hcrtr2
72472786 Dchs1
227539 Mlx
72472788 Dusp4
79591637 Grp
72472790 Fam124a
79591639 Gpr101
72472792 Epha3
72472793 Epha6
72472794 Epha8
72472797 Erbb4
72472798 Ern2
72472799 Fbn2
72472800 Fgd3
72472801 Fibcd1
72472802 Slc6a11
72472803 Galnt13
72472804 Galnt14
72472805 Gfra2
72472806 Gnai1
72472807 Gnal
72472808 Gpc2
72128745 Kcnq5
72104170 Usp20
72128747 Ankrd34b
72128748 Cux2
80517357 Cks2
72128750 Fbxw7
72128751 Zdhhc2
72128752 Zfp385b
72128753 Vstm2b
72128754 Slc22a23
72128755 Ttll7
79591669 Gad2
73636086 Fam69c
79591671 Gfap
72104184 Fabp7
73636089 Rasd2
73636090 B4galnt3
74881275 Steap3
73636092 Col23a1
73636093 Col24a1
73636094 Clic5
73636095 Pcdh17
73636096 LOC433436
121089 Vldlr
70744322 Pamr1
70744323 Stxbp6
73636100 Mas1
70744325 Osbpl6
70744326 Lppr5
72128775 Cemip
72128776 Raver2
79591689 Ube3a
72128778 Akap12
121099 Pgf
112649261 Med23
74641112 Cables2
79591699 Tyr
72128788 Apbb2
72128789 Arhgap25
72128790 Atp10a
79591703 Slc18a2
79591705 App
75080751 Rfwd2
71923998 Itpk1
73718047 Lman2
73718048 Agpat4
73718049 Elmo3
73718050 Tex40
73718051 Defb1
73718052 BC031181
73718053 Chn1
73718054 Dnajc12
71063847 Adamtsl5
71924008 Utp23
74272476 Chl1
73718058 Ccr1
79591723 Grm1
70805896 Lrrtm1
79591731 Lhx1
73592542 Zbtb24
73636150 Tmem215
73636151 Fam184b
74750264 Syt13
73636153 Iqsec3
73636154 Tmtc1
74881744 Nceh1
74881341 Doc2g
79556656 Arntl
74750263 Dab1
75749771 Cd164
73636164 Bnc2
70744391 Dnaja4
74881352 Adamtsl2
74881353 Abcb10
77371789 Cab39l
74881364 Tmem74
2446 Ogn
74881366 Il31ra
71924056 Gpr123
75749739 Crtac1
73718055 Alox8
77887842 Traip
71924067 Ano6
112204135 Vdr
77371755 Mme
70805906 Rtn4
112650122 Ebpl
77371759 Osbpl9
77887858 Psg16
77371763 Ndufv2
77887860 Sh3bgrl2
77371765 Nav1
75749751 Grik1
71924089 Asic4
71924090 Dab1
77371771 Apbb3
74047715 Lrp8
77371773 Hbb-b2
77371775 C1qa
74818624 Gemin4
77887874 Nnat
77414123 Tm2d3
77887876 Gjc1
77887878 Ano6
77371783 Slc6a20b
77887880 Tmc6
77371785 Atp5h
77887882 Tdrd3
72128919 Lingo2
77887884 Csdc2
75749773 Deptor
77887886 Gldc
77887888 Pip5k1b
71587898 Tmem35
77371797 Ctss
77371799 Postn
77371803 Aspa
77371805 Rmnd1
77371807 Ampd3
77371813 Whrn
77371815 Kcnn2
77371817 Kcns3
74882764 Ptcd1
77371821 Sstr2
71924145 Tmem255b
77371829 Alk
71670479 Grid2ip
77371831 Bcas1
71924155 Thsd7a
71836746 Scd1
77371839 Grn
77371841 Cnp
70744514 Abhd3
70744515 Kctd1
70744516 Stk32c
71924165 Vgf
70744518 Sdcbp
70744519 Klf10
205 Ccnd2
1038 Tac1
77371851 Grk5
75079789 Hmgcr
112196048 Med8
77371857 Gng7
77371859 Zfp280c
77371861 Klhl36
77371863 Kcne3
71924184 Gm1399
77371865 Rora
77371867 Rfx1
74881503 Pptc7
71064032 Bok
2470 Pcsk1
74881514 Slc35e2
74881515 Acacb
71924204 Pde10a
74881517 Apc
76085742 Nlk
76085743 Tpbg
76085744 Pbx3
76085745 Fdps
76085746 Vav3
71064051 Lzts3
76085748 Ltbp4
76085749 Sphkap
71924214 Phactr4
71924215 Ppm1l
73925716 Lancl3
71064061 Col9a1
1109 Brwd1
74881536 Atg12
71924225 Sash1
74957910 Usp13
71924235 Scube2
72079884 Foxp2
74881549 Atp2a2
71924238 Slc24a2
71924239 Unc5d
71064080 Rfk
74957912 Vdac1
71064082 Scn3b
112646675 Oprd1
70805934 Loxl1
74881559 Atp6v0c
74881560 Atp8a1
71924249 Utp14b
67936005 Rnf10
1115 Wnt8a
77371825 Ptprz1
74586665 Glipr1
74586666 Nkd1
74586667 Gpd1
71924269 Elmod1
1117 Wrb
72079921 Dclk3
71924279 Fam167a
72079931 Abat
76115722 Cebpd
71924290 Mb21d2
71924291 B2m
79583813 Fam114a2
71924301 Col25a1
74957923 0610010F05Rik
293462 Fbxo18
293463 Anapc16
293465 Rpf2
293466 Cdc27
74882831 Rpl21
293468 LOC66376
293469 Paf1
293471 Rad51
293472 Stac2
655120 Arf3
293474 Ms4a6b
77371835 Vip
79591683 Slit2
71924331 Scrg1
77414162 Ddx19b
2493 Pomc
71064176 Ppp1r9a
76115731 Klhl4
71924341 Sgtb
73931382 Frmd6
71924343 Tnmd
73931384 Cd109
75038484 Casc1
73994666 Mpp6
71924353 Clmp
71064195 Col11a1
1317 Taf1d
72080006 Tal1
112197858 Creb3l1
73521802 Bhlhe41
71924363 Decr2
71924364 Rnf220
73521805 Rab3c
73521806 Rai14
74957933 Gkn3
73521808 Stard8
73521809 Tle4
73521810 Tmsb10
73521811 Tshz3
73931412 Npr3
73931413 Pdcd4
73931414 Rnf19a
71924375 Acaa2
73931416 Trim16
73931417 Flrt2
73521818 Ptrf
73931419 LOC235953
73521820 Robo1
73931421 Frmpd4
73931422 LOC380720
73521823 Slc1a2
73931424 LOC432928
71924385 Adamts2
73931426 Syndig1
73931427 Gata3
73521828 4833414E09Rik
71924389 Tcn2
112196265 Sigmar1
72080044 Phf6
71924399 Uqcrfs1
71924400 Fhod3
71924402 Gabra4
72080054 Inpp4b
73994668 Garnl3
71924412 Greb1
72080065 Gng12
74881733 Atrx
75774664 Arhgap27
75774665 Zfp932
75774666 Hpd
75774667 Stxbp2
75774668 Fam65a
75774669 Trhde
75774670 Ubl4
75774671 Tmem246
75774672 Ptk2
75774673 Mtfp1
75774674 Hspbp1
74581424 Kcnb2
71670484 Lgi1
75774677 Arhgdia
71670486 Loxl2
71670487 Lrp4
75774680 Clip4
71924441 LOC235580
75774682 Atpif1
75774683 Nucb2
72129244 Mmp16
75774685 Pomp
75774686 Nrbp2
75774687 Rps15
72129248 Neto1
71064289 Bcl2l12
72129250 Ralyl
71924451 Cntnap3
72129252 Dpf3
72129253 Spink8
72129254 Efcab3
72129255 Hs6st2
76115730 Itgb1
112646890 Nr2f2
74988269 Asxl3
74988271 Car9
74988272 Dicer1
74988273 Dnah12
74988274 Gm22
74988275 Alpk2
112643861 Sec61a2
72080123 Nap1l5
74988285 Ascl5
74881790 Bad
71924480 Mrap2
71924481 LOC270764
72129283 Ufm1
72080133 Myo1b
72080134 Ndnf
74988296 Plcz1
72129289 Tshz1
72129290 Lipa
71924491 LOC381355
71924492 LOC545810
72129293 Lrrn1
72129294 Tshz2
72129295 Oxr1
72080144 Eps15
72080145 Rtn4r
74988306 Prdx6b
112204566 Cdkn1b
71924185 Ntng1
71924504 LOC433088
1156 Ascl2
71015194 Vill
72080155 Sdc2
71015196 Tmed3
293662 Slc7a6
293663 Prpsap2
293664 C1qtnf5
293665 Fndc4
293666 Uck1
74881827 Bap1
293668 Serpinb8
74988325 Rtl1
74357546 Slc8a3
74357547 Id4
74357548 Fgd5
74357549 Peg10
74357550 Kctd6
72080175 Trnp1
73718576 Btg1
293682 Atp6v0b
73930841 Dexi
74357559 Pou6f1
74357560 Rps15a
72080185 2310003H01Rik
74357562 Moxd1
293691 Slco3a1
293692 Aqp3
74357565 Mum1l1
293694 Zfand3
74357567 Nell1
74357568 Kcnj4
74357569 Ptk2b
74357570 Tpd52l1
74357571 Rgs5
74357572 Vat1
74357573 Tspan18
74357574 Mapk11
74357575 Cryab
74357576 Smoc2
74357577 Gdap10
74988362 Ndufaf2
74581428 Mrc1
74357580 Megf11
74357581 Pdp1
74357582 Teddm3
74357583 Sv2c
74357584 Epn3
74881878 Pkd2l1
77869838 Pgf
73997144 Mup5
73997145 Ngp
73997146 Osbpl5
73997147 Pam
73997148 Pitpnm2
73997149 Pkp2
73997150 Grb14
73997151 Rcn2
73997152 Spata13
73997153 Tspan12
74881890 R3hdm4
73931621 Lct
73931622 Svip
73931623 Arhgef26
73931624 Peak1
73931625 Chst11
73931626 Clmn
73931627 Creg1
73931628 D030063F01Rik
73931629 Rgs7bp
73931630 D8Ertd82e
73931631 Itgb8
73931632 Zfp804b
73931633 Dusp5
73931634 Gm626
73931635 Acsl5
73931636 LOC433258
73931637 Lrrtm3
73931638 Mgat5
73931640 Nova1
73931641 Pde5a
73931642 Pgbd5
73931643 Rab37
73931644 Slc22a3
77340480 Kcng1
74881927 Ppapdc1a
74881516 Actb
70560343 Chrm2
76085741 Kank4
79567760 Tbck
74881937 Arap2
79567762 Arpp21
71670677 Npnt
71670678 Npr1
71670679 Ntrk1
356489 Pigq
71670681 Pald1
71670682 Pclo
71670683 Pde7b
71670684 Cdk14
71670685 Phospho1
71670686 Inpp5j
71670687 Plekhg1
71670688 Prkg2
71670691 Tox
71670692 Zim1
74988453 Sec14l1
71670694 Pvr
74988455 Arl4c
71670696 Slc2a6
71670697 Steap2
74988458 Itgbl1
74988459 Rnaset2b
74988460 Nkx2-1
74988461 Tmem109
71670702 Ttll4
77340488 Gm12429
71670706 Txnrd3
76085747 Smarca2
71670708 Unc5c
71670709 Vangl1
71670710 Zyx
71670711 Tmem163
71670712 Zfp618
71670713 2900026A02Rik
71670714 Xlr4c
71836831 Zfand2b
71670716 Gnal
77340490 LOC433740
71670719 Rasal2
71670720 Trav3-3
71670721 Ankrd50
71670722 Kctd8
79567811 Gpr88
71670724 Ttc6
71670725 Gm196
71670726 Drc1
71670727 Rps6ka3
71670728 Serpina9
73817932 Ehbp1l1
71670730 Slc9a9
75147767 Adam33
71670733 Tas2r144
71670734 Ust
70613967 Enpp2
71670736 Zfp462
71015377 Ctsz
71670738 Pcdh20
71670739 Midn
71670740 Lrp1b
71670741 Sntb1
71670742 Slc10a4
71670743 Evi5
71670744 LOC381557
70613977 Cyp46a1
70613978 Chst15
71249743 Brinp2
71015388 Psrc1
74988509 9530068E07Rik
77280421 Emp1
74819259 Copg2
70613989 Etv5
70613990 Rgs12
74988519 Atg3
71836839 Rnf144b
71587918 Slc17a8
77280424 Plxna2
73929897 Mast4
77340500 Tob2
74988539 Eif1ax
1195 E2f4
74988549 Gm2a
2561 Sfn
74988552 Psmb2
67779875 Nde1
1197 Hook3
73817944 Tdrd3
74988562 Taldo1
2563 Sfrp1
74425518 Dynlt3
71836847 Cyp39a1
74988572 Lysmd2
74425520 Pip5k1b
74988582 Ttc25
74425521 LOC381765
2567 St3gal3
74724530 Paqr8
74988591 Gramd3
248328 Tbl3
74425523 Rps21
71015350 Mgst3
67853369 Kcnj14
1205 Gatm
539 Grm3
73635972 Lhfpl3
74988611 Kbtbd3
74988614 Mgat2
74988615 Numa1
74988616 Klk6
74881548 Atp1a4
79591633 Trhr
83167 Tm2d3
2573 Slc12a3
74988626 Rab12
71020388 Wwc1
248335 Paqr4
74988636 Rassf4
74988637 Selplg
74882147 Cntn6
112196711 Terf2ip
71020391 Tbc1d14
74988656 Scube3
74988657 Sel1l3
70612725 Tspan15
74882166 Cops4
74988667 Adcyap1r1
74988669 Cacna1e
74988670 Calcrl
70560277 S100a10
74988680 Chrnd
71924311 Igfbp4
74988690 Gabra2
74882195 Creg2
74882196 Cyp4v3
79568022 Cyp26b1
2153 Dnajb12
79568024 Lhx2
112647321 Gdnf
79568026 Cdh8
79568028 Efr3a
79568030 Mtap
74750019 Pcdh8
74988710 Grin2b
74988711 Ttyh3
71064288 Stk38l
74882217 Tmx4
74425543 Fasn
71020402 Cyb561
70612727 Crip2
79568049 Dpp4
74988722 Bsdc1
74882227 Fnip2
79568055 Snapc2
79568057 Ren2
74988733 En2
74988734 Nfic
74988735 Sf3b2
74988736 Sox8
74988737 Tcea2
74988738 Ubtf
1227 Mdk
74988740 Slc25a39
74988741 Kansl3
74988742 Cep72
74988743 Plk5
72080025 Pcsk2
74988745 Cpne3
74988746 Txnrd1
74988747 Vegfa
74988748 E030007A22Rik
74988749 Grip1
74988750 Armc2
74988751 LOC214238
74988752 5730409E04Rik
74988753 Gm973
74988754 LOC380889
74988755 Lonrf2
74988756 LOC381742
74425550 Decr1
74988758 LOC545352
74988759 Fndc3b
74988760 Acyp1
74988761 BC005561
74988762 Klhl9
74988763 Gpd1l
74988764 Hist1h1a
74988765 Hs3st2
74988766 Lamc2
74988767 Gm1335
74988768 Glul
71836880 Dbi
71836881 Gprc5b
74425554 Ldhd
1235 Nbr1
1237 Ndrg2
83158 Otud6b
71836887 Pak3
74586669 Ctxn1
83161 Rtn3
70616004 Abcd2
67779872 Cnbd2
67779873 Copg1
1243 Nr1d1
67779877 Ctsb
73907494 St8sia1
73907495 Sap30l
73907496 Gpr116
73907497 Chrm1
73907498 Fzd9
73907499 Grin3a
74581444 Pappa
73907501 Scn5a
73907502 Trpm6
73907503 Prpf38b
73907504 LOC620538
73907505 Ostf1
77413682 Prkcsh
77413684 Smpd1
77413686 Ptpn2
77413688 Vldlr
77413690 Stat5a
74425567 Clgn
76135740 Nfxl1
77413694 Tbl3
76135744 Slc39a5
73931404 Flrt3
72007563 Sema3f
76135748 P2ry14
77413702 Pcp2
77413704 Dtna
2159 Reep5
73817421 Matn2
73817422 Galnt9
73817423 Ascl1
73817424 Zcchc12
73817425 Gpr12
73817927 Arl4d
1251 Papola
73817428 Bmp5
71020430 2610028E06Rik
73817430 Capn2
73817431 Col12a1
73817432 Cpne7
73817433 Map2k6
73817434 Nefm
73817435 Ppp3ca
73817436 Ptprv
73817437 Rgs20
73817438 Rnasel
73817439 Syt9
73817440 Tnnc1
73817441 Spata2l
73817442 Arl4d
2619 Slc39a3
73817444 Dlg2
74581446 Magix
1255 Pcnt
112647536 Dkk4
75038432 Kcna2
71587931 Gpr115
76135795 Msrb2
76135799 Reps2
71015800 Fam107b
71015801 Zfp521
71015802 Elmo2
76135804 Mmel1
71015805 Eml3
71015806 Itgb1bp1
76135807 Rasgrp4
67870080 Gtf2f1
71015809 Gpr18
71015810 Resp18
67870083 Rnaseh2c
67870084 Chchd3
67870085 Cox7b
71015814 Homer2
76135815 Pknox2
71015816 1110008P14Rik
71015817 Pdia3
71015818 Trib1
76135819 Aco1
76135823 Matk
75551462 Gria2
73636121 Glra2
76135828 Tmem159
76135829 Doc2a
76135830 Deptor
74882459 Ddr2
112205214 Nr1h3
67870113 Pir
71020443 Ebf4
67755429 Gipc1
75144616 LOC544975
75144617 Nme1
75144618 Tspan2
77280341 F3
75144621 Uts2b
75144622 F630102L10Rik*
75144623 C130018E23Rik*
75144624 Sptb
79556609 Syt10
67870133 Uqcr11
2633 Sssca1
75144634 A330043P19Rik*
1890 Wnt8b
2635 Stat1
75144644 Pitpnc1
75144645 A730096I18Rik*
73817931 Tspyl2
75084969 Plcb1
74988601 Mpv17l2
67881549 Abcb6
75144656 Synpo2
74882515 Dos
74743255 Myo10
74743256 Ndufb8
74743257 Nrg1
74743258 Pacrg
74743259 Pcdhb13
74882525 Snx33
74882527 Elovl1
72283803 C230071H18Rik
74743270 Pcdhb19
74743271 Pcdhb2
74743272 Plk2
77332735 Prkar2a
74743282 Psmd11
74743283 Ptpn4
75144693 P2ry2
1892 Wnt9b
121098 Gabpb1
74882555 Enpp6
74743293 Ptpn5
2645 Stx1a
75988482 Ica1
74882565 F13a1
74743303 Ptprk
72079959 Efnb2
2711 Usp21
73616023 St3gal1
71924259 Kcns2
74882584 Fa2h
112649476 Dvl2
75988508 Slc38a1
74882595 Galnt6
199391 F2rl1
70231303 Mpped1
75988524 Timp2
75988526 Tmod1
75259440 Haghl
75259441 Mrps28
75259443 Fam3c
74882616 Hspb6
75988537 9430020K01Rik
73615803 Ntng2
74882626 Itga11
73512203 Etv1
112647750 Slc35a4
71924321 Ncald
74882636 Itga5
654925 Gabarapl1
654928 Dpysl3
654929 Fam20c
74882646 Kcnj12
75988567 Pak7
75988568 Podn
72007751 Erp29
79912542 Thra
79912544 Dlx6os1
74882657 Mfn2
75988578 Map3k19
79912548 Nrsn2
79912552 Stub1
79912554 Fos
79912556 Chrm3
77332080 Mxra7
72081545 Ticam1
77332082 Ncs1
77332086 Nmbr
77332088 1190002N15Rik
77332090 Nsun7
79908799 Foxb1
77332092 Spsb1
74882685 Hecw1
77332094 Siglech
77332096 Vwc2
77866848 Gucy1a3
75988610 Ddx26b
75988611 LOC434300
77332100 Anxa6
77332102 B4gat1
74882697 Pgrmc2
74882698 Ppp2r2c
72104186 Cdhr1
75988621 Rock2
75988622 Dgkb
75988632 Nrxn1
75988633 Tagln3
75988635 Sorcs3
75988636 Rasl11a
74882717 Prkdc
74882718 Psmd13
544709 Cpne5
79912608 Kiss1
79912613 Pcp4
75988646 D10Bwg1379e
77280540 Adcy8
74882686 Pfdn4
72119581 Cadps
544712 Cntnap2
71924388 Ly6h
72079987 Tbc1d9
75988666 Ltk
74800827 Egr4
75038402 Pnoc
67936203 BC005624
73931383 Alg2
75038412 Tnfaip8
74882765 Ptk7
74957898 Ubap1
73931385 Cspg5
67936207 Itm2c
77414110 Ak1
75038431 Drd3
74882784 Ptprr
67870433 Trem2
67870434 Inpp1
74882787 Ptprs
74882788 Purg
77414121 Zfp189
75038442 Rarb
75038443 St18
77414125 Arf1
74882798 Rfc5
74800882 Sorcs1
74800883 Uaca
75081001 Oxtr
74882808 Npvf
67936212 Tspyl4
74882810 Rpl15
2719 Wdr31
74800893 Psme2
75038463 Amotl1
75038464 Camk4
77414145 Gprc5a
67870466 Pfkm
77414147 Mlx
67870468 Rin1
77414149 Slc29a4
67936006 Gng2
74800903 Selk
74800904 Ptdss1
67936009 R74862
67936010 Pmm1
67936011 Hbb-b1
655116 Ugt8a
67870478 Slc50a1
67870479 Prkcsh
74882832 Sacm1l
1253 Pcdha10
74800914 Phyh
74800915 Nqo1
77414164 Sc5d
75038485 Cbfa2t3
75081007 Pde11a
74800925 Rnh1
70634149 Tesc
112647967 Il17rd
77414177 Hiatl1
70814342 Galnt10
77340458 Soga1
70634119 Fbxo21
77340460 B230362M20Rik
77340462 BC053994
77340464 Hbb
77340466 Hexb
77340468 Hpse
77340470 Gm1088
2697 Trps1
77340472 Kpnb1
74800953 Stard7
77340474 C1qc
77340476 Egln1
77340478 Tmem150c
32576 Mapk3
77340482 Plxnd1
2699 Tulp4
77340484 3100002H09Rik
73817925 Kcnab3
77340486 Gm6753
32583 Pim2
73817928 Gem
73817929 Slc7a3
73817930 Gyg
112205643 Cd6
77340492 Chd6
73817933 Arl16
73817934 Eef1a1
73817935 Ube2k
77340496 Ncoa7
199398 Pitx1
77340498 Fbxo44
74750042 Krt73
73931406 Lrrc55
77340502 Uqcrh
67870467 Nfs1
73521807 Slc8a1
130911 Kcnn4
130912 Kdelr1
130913 Rarg
74882915 Scd3
74882916 Sf3a2
73931409 Glrx
73931410 Ier5
74882926 Slc12a2
77340528 Scn1a
74750043 Zkscan16
73931411 Lats2
77280574 Nos1ap
108 Aqp4
74882936 Slc2a1
74882937 Slitrk1
74882938 Slitrk4
1343 Cstb
74882940 Tecta
73817983 Rufy3
73817984 Nt5dc2
73817985 Ssx2ip
73817986 Exoc6
73817987 Ssbp4
73817988 Ppp1r17
73521814 Prkcg
74882950 Tm4sf1
72007561 Hap1
72007562 Manba
73931415 Stam
72007564 Gpr171
74988456 Hebp1
74047902 St6galnac6
73521817 Prkg1
77280580 Cldn11
73931418 Gm4887
67862431 Usmg5
71064219 Dact2
74511770 Gpr50
74047400 Zdbf2
73931420 LOC329302
73521821 Sgsm1
74724680 Gpr4
74047410 Atp11c
67809267 Clic1
73521822 Slc18a3
74819332 Tro
73931423 Gm17746
73521824 Strn
112204995 Grb7
73931425 LOC433402
655307 Psg16
67936204 Eef2
73616034 Msi2
75147763 Scml4
67936208 Pcbp4
74047441 Egflam
74047442 Chml
67936211 Rpgr
74047444 Gda
75041444 Il11ra2
2725 Wnt10b
79591365 Slc6a3
74047905 Btrc
72081395 Gsn
72081396 Sptssa
112648182 Nkain4
112197625 Eno2
72081406 Car12
```

In [11]:

```
files_list = glob(os.path.join(searchpath, '*.tif'))
```

# Code Explanation, part 2¶

Random forest classification of points either inside or outside of the specified cortical area. Cortical areas are taken from the CCF. Then a cortical mask is generated using AllenSDK software and applied through the same gridding and projection process of gene expression data. Then a subset of datapoints is pulled from dilating that cortical mask, which are fed into a random forest. Number of test points total and number of test points inside the cortical area of interest are returned, as well as a visual display of points selected. The random forest is run with 100 decision trees and 100 test points, from which feature relative importance is calculated based on reduction in Gini impurity based on the gene expression pattern.

## Parameters¶

index : interger

```
Index of cortical area of interest. Relevant sub areas will also be selected.
```

name : string

```
Name of cortical area/image to be saved. Convention used was name of cortical area.
```

dilation : interger

```
Number of iterations to dilate the cortical area map by to generate dataset outside 
of desired region. 

Default is 30 iterations
```

threshold: float

```
Threshold of variable importance when generating top importance variables. Variable 
importances sum to 1.

Default is 0.00 variable importance
```

## Returns¶

'# of classifications of coordinates'

```
Total number of points pulled from each gene expression .tif image
```

'# of points inside cortical area'

```
Number of points inside the cortical area of interest
```

'maximum importance'

```
Gene with highest variable importance and its variable importance
```

'number of genes'

```
Number of genes above threshold of variable importance (default is .005 importance)
```

'Points selected'

```
Displays coordinates in the CCF. Left image is all points selected, right image is 
classification of points. Orange region is "inside" cortical region, pink region is 
"outside" cortical region.
```

'Model accuracy'

```
Confusion matrix of random forest model on 100 test coordinates. Used to ensure the 
model is doing a sufficient job of classifying points as inside or outside of 
cortical region. Also included is the variable importance histogram
```

Gene Printouts

```
Returns gene expression map of top 10 genes in terms of variable importance. Images 
are underneath gene's file name, as well as variable importance as calculated by the Gini Index.
```

In [12]:

```
def reference(coordinates, ref_image):
    values = ref_image[coordinates[:,0],coordinates[:,1]]
    print(values.shape[0], '# of classifications of coordinates')
    points_inside = (values/2).astype(int)
    print(sum(points_inside), '# of points inside cortical area')
    return values

def points(image,coordinates): 
    coordinates = coordinates.astype(int)
    size = len(coordinates)
    dataset = np.arange(size)
    x = coordinates[:,1]
    y = coordinates[:,0]
    dataset = image[y,x]
    return dataset

def machinelearning(output,references, estimators, threshold, files):
    #output and references from data pooling, number of estimators for random forest
    #threshold for variable importance
    Xtrain, Xtest, ytrain, ytest = train_test_split(output, references, test_size = 100) # splitting data
    clf = RandomForestClassifier(n_estimators = estimators, random_state=0, n_jobs = -1)
    clf.fit(Xtrain, ytrain)
    ypredict = clf.predict(Xtest)
    nonzeros, importance = outputs(clf, threshold,ypredict,ytest, files)
    return importance,nonzeros

def outputs(model, value,ypredict,ytest,files):
    importance = model.feature_importances_
    idmax = np.argmax(importance)
    topcandidate = files[idmax]
    print('maximum importance:', topcandidate, max(importance))
    nonzeros = np.where(importance> value)
    threshold = importance[nonzeros]
    print('number of genes:', threshold.shape)
    fig = plt.figure(figsize=(20,20))
    ax1 = fig.add_subplot(221)
    mat = confusion_matrix(ytest, ypredict)
    sns.heatmap(mat.T, square = True, annot = True, cmap='gray', fmt = 'd', cbar = False)
    plt.xlabel('true label')
    plt.ylabel('predicted label')
    plt.suptitle('model accuracy and variable importances', fontsize= 18)
    ax2 = fig.add_subplot(222) 
    ax2.grid(False)
    ax2 = plt.hist(threshold,log=True, color='gray') 
    plt.show()
    return nonzeros, threshold
    
def sorting(importance, nonzeros):
    terms = importance.shape[0]
    furtherexploring = np.zeros([terms,2])
    indexing = nonzeros[0]
    for x in np.arange(len(indexing)):
        furtherexploring[x,1] = importance[x]
        furtherexploring[x,0] = indexing[x]
    dataframe = pd.DataFrame(data=furtherexploring, columns=['gene','importance'])
    sortedvalues = dataframe.sort_values('importance',ascending=False)
    return sortedvalues

def topgenes(number, importance, nonzeros,coordinates, files):
    #number of desired genes returned, variable importances,
    #nonzero terms as pulled from the threshold set in output return, refimage to compare border
    sortedvalues = sorting(importance,nonzeros)
    sortedvalues = sortedvalues.set_index(np.arange(len(nonzeros[0])))
    if number > len(nonzeros[0]):
        number = len(nonzeros[0])
    top = sortedvalues.loc[(np.arange(number)),'gene']
    topvalues = sortedvalues.loc[(np.arange(number)),'importance']
    for x in np.arange(number):
        name = files[ top[x].astype(int) ]
        image = tiff.imread(name)
        image[cortextif==1] = np.nan 
        image[isocortexmask_2d==0] = np.nan
        fig = plt.figure(figsize=(13,13))
        fig.suptitle(name, fontsize=22)
        fig.text(.5,.9,topvalues[x],fontsize =15) # relative importance value
        ax1 = fig.add_subplot(111)
        ax1.grid(False)
        ax1.imshow(image, interpolation='nearest', cmap = 'magma', clim=(0,np.nanmax(image)))
    sortedvalues = sortedvalues.head(number)
    topten = sortedvalues.head(10)
    return sortedvalues, topten

def pooldata( ref_image, coordinates, files):
    # ref_image: reference image for inside/outside v1. coordinates: what points to us
    coordinates = coordinates.astype(int)
    references = reference(coordinates, ref_image)
    counter = np.array(0)
    length = (len(files))
    totalpoints = (coordinates.shape[0])
    output = np.zeros([totalpoints, length])
    for name in files:
        image = tiff.imread(name)
        output[:,counter] = points(image,coordinates)
        counter = counter + 1   
    return output, references

def displaytarget(finalreferenceimage, coordinates):
    blank = np.zeros_like(finalreferenceimage)
    blank[cortextif == 1] = 2
    ref = coordinates.astype(int)
    blank[(ref[:,0],ref[:,1])]= 1
    target = np.copy(finalreferenceimage)
    target[cortextif == 1] = 3
    target = target + blank*2
    blank[blank == 0] = np.nan
    target[isocortexmask_2d ==0 ] = np.nan
    fig = plt.figure(figsize=(20,20))
    fig.suptitle('points selected', fontsize=25)
    ax1 = fig.add_subplot(221)
    ax1.imshow(blank, interpolation='nearest', cmap ='gray', clim=(0,np.nanmax(blank)))
    ax1.grid(False)
    ax2 = fig.add_subplot(222)
    ax2.imshow(target, interpolation='nearest', cmap = 'gray', clim=(0,np.nanmax(target)))
    ax2.grid(False)
    
def coordinatesfromimage(refimage, isocortexmask_2d):
    refimage = apply_mask(refimage,isocortexmask_2d)
    point = np.where(refimage > 0)
    a = []
    a.append((point[0]))
    a.append((point[1]))
    a = np.array(a)
    a = np.swapaxes(a,0,1)
    return a

def referenceimage(name, iterations):
    ref = tiff.imread( name )
    ref_dilate = scipy.ndimage.morphology.binary_dilation(ref, iterations=iterations)
    reference_image = ref+ ref_dilate
    return reference_image

def main(index, name, dilation=30, threshold=0.00, genes = 10, trees = 100, files = files_list):
    name = name + '.tif'
    image_main_single(index,name)
    reference_image = referenceimage(name,dilation)
    coordinates = coordinatesfromimage(reference_image, isocortexmask_2d)
    output, references = pooldata( reference_image, coordinates, files)
    displaytarget(reference_image,coordinates)
    importance, nonzeros = machinelearning(output,references,trees, threshold,files)
    sortedvalues, topten = topgenes(genes,importance, nonzeros,coordinates,files)
    return sortedvalues
```

## Find Genes to Mark Desired Region¶

rerun subsequent cell as many times as desired with new indicies for new regions, new degrees of dilation

In [13]:

```
index = 329
name = "primary_somatosensory_barrel"
sortedvalues = main(index, name)
```

```
reference already created
(89100L, '# of classifications of coordinates')
(51670, '# of points inside cortical area')
('maximum importance:', 'E:/genemaps/coronal\\Nov_69120620.tif', 0.020046104684140423)
('number of genes:', (4344L,))
```
