# Supplementary material for: Identification of genetic markers for cortical areas using a Random Forest classification routine and the Allen Mouse Brain Atlas
Source: PLoS One. 2019 Sep 4;14(9):e0212898. doi: 10.1371/journal.pone.0212898 (PMC6726226; doi:10.1371/journal.pone.0212898)
Supplement: S4 Fig — Jupyter notebook code for generation and analysis of horizontal projections, html version. (HTML) [file pone.0212898.s004.html]

Flatmap\_Projection


## *Only modify "system specific modifications" and "find genes to mark specific region" box*¶

- Download attached files and point via system specific modifications to: CCF\_borders\_10um.tif, top\_view\_paths\_10.h5, gridAnnotation.mhd via "system specific modifications" cell
- Preallocate a folder for storing gene expression top view images, designated as searchpath

  - MAKE FOLDER PRIOR TO RUNNING CELL

In [1]:

```
import os
import numpy as np
import nrrd
import scipy.ndimage
import matplotlib.pyplot as plt
%matplotlib inline
from glob import glob
from sklearn.ensemble import RandomForestClassifier
from sklearn.model_selection import train_test_split
import tifffile as tiff
from sklearn.metrics import confusion_matrix
import seaborn as sns; sns.set()
import pandas as pd
import h5py
import SimpleITK as sitk 
import urllib, urllib2
import json
import zipfile
import scipy.stats as stats

from allensdk.core.mouse_connectivity_cache import MouseConnectivityCache
mcc = MouseConnectivityCache(manifest_file='connectivity/manifest.json', resolution=10)
from allensdk.api.queries.ontologies_api import OntologiesApi
from allensdk.core.structure_tree import StructureTree
from allensdk.core.reference_space import ReferenceSpace
from allensdk.api.queries.mouse_connectivity_api import MouseConnectivityApi
from allensdk.config.manifest import Manifest
```

#### System specific modifications¶

In [23]:

```
# File Locations
## CCF_borders_10um.tif LOCATION: 
cortextif_path = r"flatmapmask.tif"

## paths view file location LOCATION:
flatmap_view_file = r"dorsal_flatmap_paths_10.h5"

## gridAnnotation.mhd LOCATION:
annot_200_img = sitk.ReadImage(r"gridAnnotation.mhd")

# Where images will be saved to
## Make a folder for gene maps to be directed to prior to running this cell
searchpath = r'topviews/'
```

##### Making the reference space, as taken from AllenSDK¶

In [3]:

```
# the annotation download writes a file, so we will need somwhere to put it
annotation_dir = 'annotation'
Manifest.safe_mkdir(annotation_dir)

annotation_path = os.path.join(annotation_dir, 'annotation.nrrd')

# this is a string which contains the name of the latest ccf version
annotation_version = MouseConnectivityApi.CCF_VERSION_DEFAULT

mcapi = MouseConnectivityApi()
mcapi.download_annotation_volume(annotation_version, 10, annotation_path)

annotation, meta = nrrd.read(annotation_path)

oapi = OntologiesApi()
structure_graph = oapi.get_structures_with_sets([1])  # 1 is the id of the adult mouse structure graph

# This removes some unused fields returned by the query
structure_graph = StructureTree.clean_structures(structure_graph)  

tree = StructureTree(structure_graph)

# build a reference space from a StructureTree and annotation volume, the third argument is 
# the resolution of the space in microns
rsp = ReferenceSpace(tree, annotation, [10, 10, 10])
```

```
2019-02-03 17:36:38,025 allensdk.api.api.retrieve_file_over_http INFO     Downloading URL: http://download.alleninstitute.org/informatics-archive/current-release/mouse_ccf/annotation/ccf_2017/annotation_10.nrrd
```

## Isocortex mask¶

Make Isocortex mask in 2 dims and 3 dims

In [4]:

```
cortex = tree.descendant_ids([315])[0]
annot_200 = sitk.GetArrayFromImage(annot_200_img)
annot_200 = np.swapaxes(annot_200, 0, 2)

isocortex_200_mask = np.zeros_like(annot_200, dtype=np.uint8)
for sid in cortex:
    isocortex_200_mask[annot_200 == sid] = 1
```

# Code Explanation, part 1¶

Mapping of gene expression data from the mouse brain atlas coronal data to common coordinate frame using projections along paths to edge of cortex, and subsequent 'uncurling' of cortex to a horizontal plane as viewed from the top of the brain. This code will pull data from the Allen server and create tif images of each experiment, storing the images in the path that the notebook is stored in. If images are already generated (with proper nomenclature), they will not be recreated. Images are named as "genename\_experimentnumber\_flat.tif". No external variables are inputted. No variables are explicitly returned. Images are saved to searchpath. Names of mice are printed. Dash is printed after name of mouse if flat map view image already exists in folder.

Once images are generated, the specific mice identification can also be printed out using the view\_mouse function.

In [122]:

```
#global files
files_list = glob(os.path.join(searchpath, '*.tif'))
cortextif = tiff.imread(cortextif_path)
```

In [104]:

```
def project(data, view, paths,  output):
    for index, path_id in enumerate(view.flat):
        path = paths[path_id, :]
        path = path[path != 0] # paths are a zero-padded ragged array

        output.flat[index] = np.amax(data.flat[path])

    return output

def create_flatmap_top_view_projection(data):
    dims_10 = (1320, 800, 1140)
    dims_200 = (67, 41, 58)
    f = h5py.File(flatmap_view_file, 'r')
    view_paths = f['paths'].value
    view_lookup = f['view lookup'].value
    
    path_200 = np.array(np.unravel_index(view_paths, dims_10))
    path_20 = np.ravel_multi_index((path_200 / 20).astype(int), dims_200)

    view_size = view_lookup.shape
    paths_size = path_20.shape
    
    out_view = np.zeros(view_size, dtype=data.dtype)
    out_view = project(data, view_lookup, path_20,  out_view)

    return out_view

def create_flatmap(section_id, gene_name, mask):
    filename = os.path.normpath(searchpath + gene_name + "_" + str(section_id) + ".tif")
    if os.path.isfile(filename):
        print "_"
    else:
        data = get_section_image(section_id)
        data = apply_mask(data, mask) # to apply isocortex mask
        output = create_flatmap_top_view_projection(data)
        output = output.astype(float)
        tiff.imsave(filename, output)
        
def get_section_image(section_id):
    grid_download_template = "http://api.brain-map.org/grid_data/download/{:s}?include=density"

    grid_download_url = grid_download_template.format(str(section_id))
    urllib.urlretrieve(grid_download_url, "griddata.zip")

    with zipfile.ZipFile("griddata.zip", "r") as zip_ref:
        zip_ref.extractall("griddata")

    img = sitk.ReadImage("griddata/density.mhd")
    arr = sitk.GetArrayFromImage(img)
    arr = np.swapaxes(arr, 0, 2)
    return arr
        
def flatmaps_main():
    f = h5py.File(flatmap_view_file, 'r')
    rma_url = ("http://api.brain-map.org/api/v2/data/query.json?criteria=" +
               "model::SectionDataSet,rma::criteria,[failed$eqFalse]," +
               "plane_of_section[name$eqcoronal],products[name$eq'Mouse%20Brain']," +
               "treatments[name$eqISH],rma::include,genes," +
               "rma::options[only$eq'genes.acronym,id'][num_rows$eqall]")
    
    response = urllib2.urlopen(rma_url).read()
    data = json.loads(response)
    sections = {v["id"]: v["genes"][0]["acronym"] for v in data["msg"]}

    for section_id in sections:
        print sections[section_id].replace('*', '_')
        create_flatmap(section_id, sections[section_id].replace('*', '_'), isocortex_200_mask)
        
def reference_flatmap_top_view_projection(data):
    dims_10 = (1320, 800, 1140)
    
    f = h5py.File(flatmap_view_file, 'r')
    view_paths = f['paths'].value
    view_lookup = f['view lookup'].value
    
    path_200 = np.array(np.unravel_index(view_paths, dims_10))
    path_same = np.ravel_multi_index((path_200).astype(int), dims_10)
    
    view_size = view_lookup.shape
    paths_size = path_200.shape
    
    out_view = np.zeros(view_size, dtype=data.dtype)
    out_view = project(data, view_lookup, path_same,  out_view)

    return out_view
        
def create_flatmap_single(data, name):
    output = reference_flatmap_top_view_projection(data)
    output = output.astype(float)
    tiff.imsave(name,output)
        
def flat_image_main_single(index,name):
    filename = os.path.normpath(name) 
    if os.path.isfile(filename):
        print "reference already created"
    else:
        data = rsp.make_structure_mask([index])
        create_flatmap_single(data, name)
        
def apply_mask(griddata, mask):
    griddata[mask == 0] = -1
    return griddata
```

## Generate Flatmap Projections¶

In [106]:

```
name = 'isocortex_flatmap.tif'
flat_image_main_single(315,name)
isocortexmask_2d_flat = tiff.imread('isocortex_flatmap.tif')
isocortexmask_2d_flat[isocortexmask_2d_flat==-1] = np.nan
```

```
reference already created
```

In [100]:

```
flatmaps_main()
```

```
Inpp5a
_
Snap47
_
Nvl
_
Gtf3c2
_
Acadl
_
Baiap3
_
S100a6
_
Clock
_
Mageh1
_
Sncg
_
Hnrnph2
Nnat
```

```
---------------------------------------------------------------------------
KeyboardInterrupt                         Traceback (most recent call last)
<ipython-input-100-f8c9c894ef69> in <module>()
----> 1 flatmaps_main()

<ipython-input-92-9c2855c77fbb> in flatmaps_main()
     65     for section_id in sections:
     66         print sections[section_id].replace('*', '_')
---> 67         create_flatmap(section_id, sections[section_id].replace('*', '_'), isocortex_200_mask)
     68 
     69 def reference_flatmap_top_view_projection(data):

<ipython-input-92-9c2855c77fbb> in create_flatmap(section_id, gene_name, mask)
     33         data = get_section_image(section_id)
     34         data = apply_mask(data, mask) # to apply isocortex mask
---> 35         output = create_flatmap_top_view_projection(data)
     36         output = output.astype(float)
     37         tiff.imsave(filename, output)

<ipython-input-92-9c2855c77fbb> in create_flatmap_top_view_projection(data)
     12     dims_200 = (67, 41, 58)
     13     f = h5py.File(flatmap_view_file, 'r')
---> 14     view_paths = f['paths'].value
     15     view_lookup = f['view lookup'].value
     16 

h5py/_objects.pyx in h5py._objects.with_phil.wrapper()

h5py/_objects.pyx in h5py._objects.with_phil.wrapper()

/Users/njweed/anaconda2/lib/python2.7/site-packages/h5py/_hl/dataset.pyc in value(self)
    248         DeprecationWarning("dataset.value has been deprecated. "
    249             "Use dataset[()] instead.")
--> 250         return self[()]
    251 
    252     @property

h5py/_objects.pyx in h5py._objects.with_phil.wrapper()

h5py/_objects.pyx in h5py._objects.with_phil.wrapper()

/Users/njweed/anaconda2/lib/python2.7/site-packages/h5py/_hl/dataset.pyc in __getitem__(self, args)
    494         mspace = h5s.create_simple(mshape)
    495         fspace = selection.id
--> 496         self.id.read(mspace, fspace, arr, mtype, dxpl=self._dxpl)
    497 
    498         # Patch up the output for NumPy

KeyboardInterrupt:
```

# Code Explanation, part 2¶

Random forest classification of points either inside or outside of the specified cortical area. Cortical areas are taken from the CCF. Then a cortical mask is generated using AllenSDK software and applied through the same gridding and projection process of gene expression data. Then a subset of datapoints is pulled from dilating that cortical mask, which are fed into a random forest. Number of test points total and number of test points inside the cortical area of interest are returned, as well as a visual display of points selected. The random forest is run with 100 decision trees and 100 test points, from which feature relative importance is calculated based on reduction in Gini impurity based on the gene expression pattern.

## Parameters¶

index : interger

```
Index of cortical area of interest. Relevant sub areas will also be selected.
```

name : string

```
Name of cortical area/image to be saved. Convention used was name of cortical area.
```

dilation : interger

```
Number of iterations to dilate the cortical area map by to generate dataset outside 
of desired region. 

Default is 30 iterations
```

threshold: float

```
Threshold of variable importance when generating top importance variables. Variable 
importances sum to 1.

Default is 0.00 variable importance
```

## Returns¶

'# of classifications of coordinates'

```
Total number of points pulled from each gene expression .tif image
```

'# of points inside cortical area'

```
Number of points inside the cortical area of interest
```

'maximum importance'

```
Gene with highest variable importance and its variable importance
```

'number of genes'

```
Number of genes above threshold of variable importance (default is .005 importance)
```

'Points selected'

```
Displays coordinates in the CCF. Left image is all points selected, right image is 
classification of points. Orange region is "inside" cortical region, pink region is 
"outside" cortical region.
```

'Model accuracy'

```
Confusion matrix of random forest model on 100 test coordinates. Used to ensure the 
model is doing a sufficient job of classifying points as inside or outside of 
cortical region. Also included is the variable importance histogram
```

Gene Printouts

```
Returns gene expression map of top 10 genes in terms of variable importance. Images 
are underneath gene's file name, as well as variable importance as calculated by the Gini Index.
```

In [125]:

```
def reference(coordinates, ref_image):
    values = ref_image[coordinates[:,0],coordinates[:,1]]
    print(values.shape[0], '# of classifications of coordinates')
    points_inside = (values/2).astype(int)
    print(sum(points_inside), '# of points inside cortical area')
    return values

def points(image,coordinates): 
    coordinates = coordinates.astype(int)
    size = len(coordinates)
    dataset = np.arange(size)
    x = coordinates[:,1]
    y = coordinates[:,0]
    dataset = image[y,x]
    return dataset

def machinelearning(output,references, estimators, threshold, files):
    #output and references from data pooling, number of estimators for random forest
    #threshold for variable importance
    Xtrain, Xtest, ytrain, ytest = train_test_split(output, references, test_size = 100) # splitting data
    clf = RandomForestClassifier(n_estimators = estimators, random_state=0, n_jobs = -1)
    clf.fit(Xtrain, ytrain)
    ypredict = clf.predict(Xtest)
    nonzeros, importance = outputs(clf, threshold,ypredict,ytest, files)
    return importance,nonzeros

def outputs(model, value,ypredict,ytest,files):
    importance = model.feature_importances_
    idmax = np.argmax(importance)
    topcandidate = files[idmax]
    print('maximum importance:', topcandidate, max(importance))
    nonzeros = np.where(importance> value)
    threshold = importance[nonzeros]
    print('number of genes:', threshold.shape)
    fig = plt.figure(figsize=(20,20))
    ax1 = fig.add_subplot(221)
    mat = confusion_matrix(ytest, ypredict)
    sns.heatmap(mat.T, square = True, annot = True, cmap='gray', fmt = 'd', cbar = False)
    plt.xlabel('true label')
    plt.ylabel('predicted label')
    plt.suptitle('model accuracy and variable importances', fontsize= 18)
    ax2 = fig.add_subplot(222) 
    ax2.grid(False)
    ax2 = plt.hist(threshold,log=True, color='gray') 
    plt.show()
    return nonzeros, threshold
    
def sorting(importance, nonzeros):
    terms = importance.shape[0]
    furtherexploring = np.zeros([terms,2])
    indexing = nonzeros[0]
    for x in np.arange(len(indexing)):
        furtherexploring[x,1] = importance[x]
        furtherexploring[x,0] = indexing[x]
    dataframe = pd.DataFrame(data=furtherexploring, columns=['gene','importance'])
    sortedvalues = dataframe.sort_values('importance',ascending=False)
    return sortedvalues

def topgenes_flat(number, importance, nonzeros,coordinates, files, isocortexmask_2d):
    #number of desired genes returned, variable importances,
    #nonzero terms as pulled from the threshold set in output return, refimage to compare border
    sortedvalues = sorting(importance,nonzeros)
    sortedvalues = sortedvalues.set_index(np.arange(len(nonzeros[0])))
    if number > len(nonzeros[0]):
        number = len(nonzeros[0])
    top = sortedvalues.loc[(np.arange(number)),'gene']
    topvalues = sortedvalues.loc[(np.arange(number)),'importance']
    for x in np.arange(number):
        name = files[ top[x].astype(int) ]
        image = tiff.imread(name)
        image[cortextif==1] = np.nan 
        image[isocortexmask_2d==0] = np.nan
        image[image==-1] = np.nan
        fig = plt.figure(figsize=(13,13))
        fig.suptitle(name, fontsize=22)
        fig.text(.5,.9,topvalues[x],fontsize =15) # relative importance value
        ax1 = fig.add_subplot(111)
        ax1.grid(False)
        ax1.imshow(image, interpolation='nearest', cmap = 'magma', clim=(0,np.nanmax(image)))
    sortedvalues = sortedvalues.head(number)
    topten = sortedvalues.head(10)
    return sortedvalues, topten

def pooldata( ref_image, coordinates, files):
    # ref_image: reference image for inside/outside v1. coordinates: what points to us
    coordinates = coordinates.astype(int)
    references = reference(coordinates, ref_image)
    counter = np.array(0)
    length = (len(files))
    totalpoints = (coordinates.shape[0])
    output = np.zeros([totalpoints, length])
    for name in files:
        image = tiff.imread(name)
        output[:,counter] = points(image,coordinates)
        counter = counter + 1   
    return output, references

def displaytarget_flat(finalreferenceimage, coordinates,isocortexmask_2d):
    blank = np.zeros_like(finalreferenceimage)
    blank[cortextif == 1] = 2
    ref = coordinates.astype(int)
    blank[(ref[:,0],ref[:,1])]= 1
    target = np.copy(finalreferenceimage)
    target[cortextif == 1] = 3
    target = target + blank*2
    target[isocortexmask_2d ==0 ] = np.nan
    fig = plt.figure(figsize=(20,20))
    fig.suptitle('points selected', fontsize=25)
    ax1 = fig.add_subplot(221)
    ax1.imshow(blank, interpolation='nearest', cmap ='gray', clim=(0,np.nanmax(blank)))
    ax1.grid(False)
    ax2 = fig.add_subplot(222)
    ax2.imshow(target, interpolation='nearest', cmap = 'gray', clim=(0,np.nanmax(target)))
    ax2.grid(False)
    
def coordinatesfromimage(refimage, isocortexmask_2d):
    refimage = apply_mask(refimage,isocortexmask_2d)
    point = np.where(refimage > 0)
    a = []
    a.append((point[0]))
    a.append((point[1]))
    a = np.array(a)
    a = np.swapaxes(a,0,1)
    return a

def referenceimage(name, iterations):
    ref = tiff.imread( name )
    ref_dilate = scipy.ndimage.morphology.binary_dilation(ref, iterations=iterations)
    reference_image = ref+ ref_dilate
    return reference_image

def main_flatmap(index, name,isocortexmask_2d = isocortexmask_2d_flat,dilation=30, threshold=0.00, genes = 10, trees = 100, files = files_list):
    name = name + '_flat.tif'
    flat_image_main_single(index,name)
    reference_image = referenceimage(name,dilation)
    coordinates = coordinatesfromimage(reference_image, isocortexmask_2d)
    output, references = pooldata(reference_image, coordinates, files)
    displaytarget_flat(reference_image,coordinates, isocortexmask_2d)
    importance, nonzeros = machinelearning(output,references,trees, threshold,files)
    sortedvalues, topten = topgenes_flat(genes,importance, nonzeros,coordinates,files, isocortexmask_2d)
    return sortedvalues
```

## Run Cells¶

In [126]:

```
name = 'frontal_pole'
main_flatmap(184, name, isocortexmask_2d = isocortexmask_2d_flat)
```

```
reference already created
(76852, '# of classifications of coordinates')
(38404, '# of points inside cortical area')
('maximum importance:', 'topviews/Serpinf1_2559.tif', 0.25903380897279615)
('number of genes:', (14,))
```

Out[126]:

|  | gene | importance |
| --- | --- | --- |
| 0 | 11.0 | 0.259034 |
| 1 | 7.0 | 0.085184 |
| 2 | 0.0 | 0.077512 |
| 3 | 13.0 | 0.076847 |
| 4 | 5.0 | 0.070924 |
| 5 | 1.0 | 0.070862 |
| 6 | 6.0 | 0.063586 |
| 7 | 12.0 | 0.057570 |
| 8 | 8.0 | 0.056659 |
| 9 | 3.0 | 0.046214 |
